# In Silico Decoding of Nonsteroidal Mineralocorticoid Receptor Antagonist Binding: Implications for Drug Design

**DOI:** 10.1021/acs.jcim.6c00183

**Published:** 2026-08-15

**Authors:** Felipe Pérez-Gordillo, Laureano E. Carpio, Diego Alvarez de la Rosa, Mercedes Martín-Martínez

**Affiliations:** † Instituto de Química Médica (IQM-CSIC), Juan de la Cierva 6, Madrid 28006, Spain; ‡ MolDrug AI Systems SL, Parque Tecnológico de Valencia, Valencia 46980, Spain; § 16749 Universidad de La Laguna, La Laguna 382000, Spain

## Abstract

The mineralocorticoid receptor (MR), a member of the steroid hormone nuclear receptor subfamily, mediates the effects of aldosterone and glucocorticoids. Initially characterized in epithelial tissues as a key regulator of blood pressure, MR was later found to be widely expressed across multiple tissues, where it mediates processes such as inflammation, oxidative stress, and fibrosis. Accordingly, MR antagonists (MRAs) have important clinical applications beyond their diuretic effect, forming a cornerstone in the treatment of chronic heart failure and showing promise in conditions like diabetic nephropathy. Nonsteroidal MRAs typically show fewer off-target effects than their steroidal counterparts, although the risk of hyperkalemia—especially in patients with renal impairment—remains a concern. A detailed understanding of MRA-binding modes is therefore crucial for the development of improved therapeutics. To this end, we conducted an extensive computational study integrating induced fit docking (IFD) and molecular dynamics (MD) simulations across a comprehensive set of MRAs. This approach highlights key interactions responsible for binding affinity and offers insights into the molecular basis of selectivity and antagonism. Moreover, MD simulations revealed a potential link between the ligand interaction with Trp806 and coregulator recruitment. These findings provide a valuable foundation for in silico rational design of next-generation MRAs with enhanced safety and efficacy profiles.

## Graphical abstract

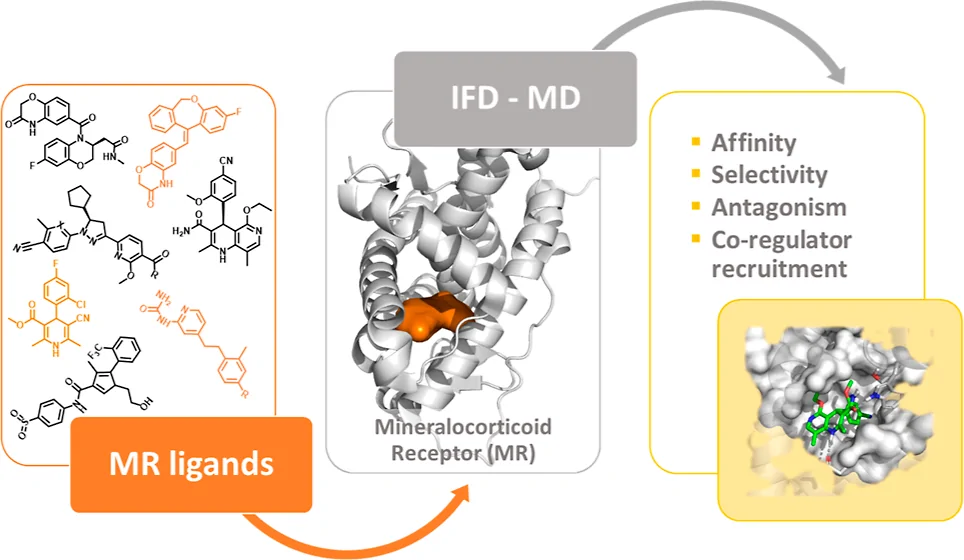

## 1 Introduction

The mineralocorticoid receptor (MR) is a member of the 3-oxosteroid hormone nuclear receptor subfamily (NR3C), together with the receptors for glucocorticoids (GR), progesterone (PR), and androgens (AR).^1^ Both aldosterone and glucocorticoids bind MR with high affinity, activating the receptor, which acts as a transcription factor, that selectively regulates gene expression in a tissue- and ligand-specific way.^2^ In certain cell types, MR selectivity for aldosterone over glucocorticoids is determined primarily by 11β-hydroxysteroid dehydrogenase type 2 (HSD2), which inactivates glucocorticoids and thereby allows aldosterone preferential access to the receptor.^3^ Selective aldosterone/MR activity is responsible for increased Na^+^ reabsorption and K^+^ and H^+^ excretion in electrically tight epithelia, such as the kidney distal tubule and collecting duct or the distal colon.^2,4^ Renal aldosterone/MR is thus essential for electrolyte homeostasis and plays a central role in blood pressure regulation. Consequently, MR antagonists (MRAs) have been used as K^+^-sparing diuretics in the treatment of hypertension.^5,6^ In the past two decades, the use of MRAs has significantly expanded due to their beneficial effects in treating heart failure, independent of their effect on lowering blood pressure.^5,6^ The protective effect of MRAs mainly arises from the involvement of the receptor in promoting inflammation, oxidative stress, and fibrosis.^7,8^ This has greatly expanded the potential of MR as a therapeutic target, with new applications in the treatment of diabetic nephropathy and other conditions.^9^ Additionally, MR signaling in the central nervous system, where it regulates stress responses, memory, learning, and neuronal differentiation, has expanded the potential therapeutic applications of MRAs to include stress-related psychiatric disorders.^10^

The first generation of MRAs, represented by spironolactone, is still used in clinical settings, but it is limited by off-target effects—particularly, cross-modulation of receptors within the NR3C subfamily—as well as potential deleterious effects such as the induction of hyperkalemia, which in turn is a concern in patients with renal insufficiency. These limitations have driven the search for newer MRAs with improved safety and efficacy.^6,9^ A second generation of MRAs became available with eplerenone, which showed much higher selectivity for MR over PR and AR, eliminating sexual side effects. However, this came at the cost of lower MR affinity and reduced efficacy in lowering blood pressure compared to spironolactone. Finerenone and esaxerenone emerged as third-generation MRAs with fewer adverse effects than earlier steroidal antagonists.^6,9^ However, there are still issues such as certain risk of hyperkalemia, in particular, in patients with concomitant renal disease.^11^ This poses a significant challenge, especially in the treatment of conditions like diabetic kidney disease (DKD), which is associated with a higher risk of cardiovascular events. Therefore, the development of novel antagonists that can selectively inhibit the detrimental effects of MR in nonepithelial cells, while preserving its physiological function in the kidney, is of great interest.

The pharmacological relevance of MR has motivated extensive efforts to discover new ligands, leading to the identification of diverse compound families with different scaffolds.^12,13^ Structural studies have revealed the 3D conformation of several derivatives bound to the ligand-binding domain (LBD) of MR, providing valuable insights into the structural requirements for ligand interaction. In general, agonist binding to MR, in contrast to antagonist binding, involves an extensive network of hydrogen bonds, similar to other steroid nuclear receptors and their physiological ligands.^2^ Agonist binding induces a rotation of helix 12, which occludes the hydrophobic binding pocket, while a conformational change affecting helices 3, 5, and 11 forms a hydrophobic cleft on the surface of the LBD, facilitating the recruitment of transcriptional coregulators harboring a LxxLL motif^14–16^ to AF2. In addition, ligand binding induces changes in the LBD that initiate receptor dimerization, a process that also involves the DNA-binding domain (DBD). Recently, it has been shown that MR adopts a diversity of ligand- and DNA-induced quaternary structures, including tetramers and even higher-order oligomers, with the oligomer size tracking the binding of agonists or antagonists.^17^ However, the allosteric transitions that link ligand binding to the rearrangement of the LBD surface, interdomain communications, coregulator recruitment, and/or oligomerization are poorly understood. In addition, the binding modes of many compound families remain uncharacterized. Even when crystal structures are available, they often fail to capture the full range of substituents within a given family or to adequately explain the molecular determinants of receptor selectivity and functional activity. To address these limitations, we conducted a thorough study combining induced fit docking (IFD) and molecular dynamics (MD) simulations to explore the binding of each compound family in detail, with particular attention to the influence of substituents on binding affinity and selectivity against other steroid receptors (GR, AR, PR). We also performed a comparative analysis across compounds’ families to identify shared and divergent structural features. This comprehensive understanding of key structural features in current MR ligands will provide the foundation for the rational design of safer, more selective therapeutic agents.

## 2 Methods

### 2.1 Data Set Assembly and Curation

To enable a comprehensive analysis of MRAs, a data set of over 700 compounds was assembled from patents and peer-reviewed publications identified through targeted searches in SciFinder. The data set was designed to provide a broad coverage of chemically diverse MR ligand series reported in the literature.^63^ Compound selection involved manual curation of the retrieved sources, retaining structures with reported bioactivity and clear structural definition. Redundant entries appearing across multiple publications or patents were removed after structural comparison, ensuring a nonduplicated set of compounds representative of the different chemotypes.

To organize the data set, compounds were grouped into clusters based on structural similarity. This process combined literature-based classification, where compounds are often presented as series within individual patents or publications, with manual curation to ensure chemical consistency across the data set. Within each cluster, representative compounds were selected for further analysis, prioritizing (i) high-affinity ligands and (ii) compounds for which small structural modifications were associated with significant changes in activity or selectivity. This selection strategy enabled the identification of structure activity relationship (SAR) trends and key interaction features relevant to MR binding.

### 2.2 Rigid Docking Studies

The MR ligand binding domain was obtained from the PDB structure of code 2A3I^16^ and processed with the Protein Preparation Wizard tool within the Schrödinger suite (Protein Preparation Wizard 2015–4; Epik version 2.4, S., LLC, New York, NY, 2015; Impact version 5.9, Schrödinger, LLC, New York, NY, 2015; Prime version 3.2, Schrödinger, LLC, New York, NY, 2015).^18^ The ligands were built in Maestro 2015–4 (Maestro, Schrödinger, LLC, New York, NY, 2015) and prepared using the LigPrep application in the Schrödinger suite of programs.^19^

Docking grids were generated by centering the grid box on the cocrystallized ligand present in 2A3I. Rigid docking was carried out using Glide within the Schrödinger suite of programs (Glide, Schrödinger Suite 2022–1; Schrödinger, LLC, New York, NY, USA)^19,20^ with default parameters. In this protocol, the van der Waals radii of ligand atoms were scaled by a factor of 0.8 to allow limited tolerance for close contacts, while keeping the protein backbone and side chains rigid. Full ligand flexibility was allowed during docking. Docked poses were ranked using the GlideScore scoring function and visual inspection.

### 2.3 Induced Fit Docking Studies

The MR LBDs were obtained from the PBD structure codes 2AA2,^14^ 2A3I,^16^ 3VHV,^21^ 3WFF, 3WFG,^22^ 4PF3,^23^ 5HCV,^24^ 5L7E, and 5L7G^25^ and processed according to the method indicated in the Rigid Docking section. The ligands were built and prepared as indicated in the Rigid Docking section. IFD calculations were performed following the Schrödinger IFD protocol (2015–4).^26,27^ The docking grid was defined as a cubic box centered on the cocrystallized ligand. Side chains within 5 Å from the ligand were allowed to move during the simulation. The procedure involved an initial Glide docking step in which the van der Waals radii (rdW) of both protein and ligand were scaled by 0.5 to minimize steric clashes, followed by side chain optimization of residues within 5 Å of the ligand using Prime (Schrödinger, LLC, New York, NY, 2015).^28,29^ Subsequently, ligands were redocked with Glide using the default rdW radii into the new MR LBD conformation. Poses were ranked by IFD score and selected based on top poses and visual inspection. Visualization and figure generation were carried out with the PyMOL Molecular Graphic System v2.3.3 (Schrödinger Inc., München, Germany).

### 2.4 Molecular Dynamics Studies

The top ranked IFD poses for **6**,**12**, **20**, **23**, **26**, **35**, **37**, **39**, **46**, **48,** and **53** were selected for further analysis of their binding modes through triplicate MD simulations using Amber 20 with the FF19SB force field.^30^ This approach accounts for the full flexibility of the protein. Each MR LBD-ligand complex was solvated in an octahedral box using TIP3P^31^ water molecules, with each site at least 10 Å from any complex atom, and periodic boundary conditions were applied. Energy minimization was performed for 30000 steps using the steepest descent algorithm, starting with a positional restraint weight of 5.0 kcal mol^–1^ A^–2^, which was gradually reduced and removed during the last 10000 steps. The system was then equilibrated over 125 ps with an initial positional restraint weight of 5.0 kcal mol^–1^ A^–2^ and a 1.0 fs integration time step. Water molecules were equilibrated for 100 ps while the solutes remained restrained. The restraints were progressively reduced and completely removed during the last 25 ps. Temperature was maintained at 300 K using Langevin dynamics. After equilibration, a 1000 ns production simulation was carried out under the *NPT* ensemble, with 2.0 fs integration time step, employing the SHAKE algorithm to constrain bonds involving hydrogen. Electrostatic interactions were calculated using the particle mesh Ewald method. Visualization and figure generation were performed with the PyMOL Molecular Graphic System v2.3.3 (Schrödinger Inc., München, Germany).

### 2.5 Data Analysis and Visualization

Trajectory analyses were performed using CPPTRAJ from the AMBER software suite. Prior to analysis, trajectories were autoimaged using the protein as an anchor to account for periodic boundary conditions. Root-mean-square deviation (RMSD) values were calculated using the first frame of the production trajectory as reference.

Protein RMSD was computed by aligning all trajectory frames to the reference structure using the Cα atoms of the protein backbone. Ligand RMSD was subsequently calculated without additional fitting, preserving the protein-based alignment to evaluate ligand stability relative to the bound protein conformation.

Total system energies were extracted from the simulations. For each system, three independent molecular dynamics replicas were analyzed. RMSD and energy values were averaged over the production phase of each replica, and inter-replica variability was assessed by comparing replica-averaged values. Data processing and visualization were performed using in-house Python scripts (version 3.9.4), and results were represented using box-and-whisker plots.

## 3 Results and Discussion

To gain a comprehensive understanding of the binding modes of different MRAs, we compiled a data set of over 700 compounds from patents and publications. This data set provided the foundation for our analysis aimed at identifying key structural features and patterns associated with MR binding. The first step involved clustering compounds based on structural similarity. Within each cluster, we selected high-affinity ligands and molecules in which minor modifications led to significant changes in affinity or selectivity for further study. We then applied an Induced Fit Docking (IFD) protocol to analyze their binding to MR. This approach allows flexibility of side chains within a 5 Å radius from the ligand while keeping a rigid protein backbone. IFD is particularly suitable in this context, given the broad chemical diversity and variety of scaffolds represented in the ligand data set. For comparison, rigid docking was also performed for the DHP family to assess whether this simpler approach could generate poses consistent with the known structure-activity relationship within this family, as discussed below. In addition, as an initial step, we evaluated whether the use of a single MR crystal structure could provide a comprehensive representation of ligand binding across the entire compound set. To this end, the structure with PDB code 2A3I (MR bound to the steroid corticosterone, **2**)^16^ was selected as the initial docking template. As expected, this approach did not generate suitable binding poses for all compounds. Therefore, to account for partial protein backbone flexibility, we performed IFD with additional MR LBD structures cocrystallized with diverse ligands, including the steroid aldosterone (**1**, PDB code 2AA2)^14^ and a range of synthetic derivatives bearing a benzoxazinone ring such as in compounds **3**–**7** ^21–24^ or sulfonamide compounds **8** and **9** ^25^ (Figure 1, Supporting Information Table S1). Although a recent approach analyzed steroidal antagonist binding to MR LBD without truncation,^32^ previous molecular docking studies using MR structures with PDB codes 2ABI ^33^ and 2A3I^34,35^ suggested that excluding helix H12 avoids steric interference when docking bulky antagonists. Accordingly, we performed a preliminary docking exploration with a subset of compounds, including several with known 3D structures in complex with MR, both in the presence and absence of MR H12. IFD including H12 produced fewer poses and successfully replicated known ligand-binding modes. Hence, subsequent IFD studies were conducted with H12 included, unless otherwise indicated. Top-ranking poses were visually inspected for complementarity with the binding pocket, with particular emphasis on their potential to form hydrogen bonds with at least one of the residues known to play a critical role in MR ligand binding: Asn770, Gln776, Ser810, Arg817, and Thr945. Additionally, the poses were evaluated for their ability to explain known structure-activity relationships (SARs). For clarity, our results are organized by structurally related compound families.

**Figure 1:**
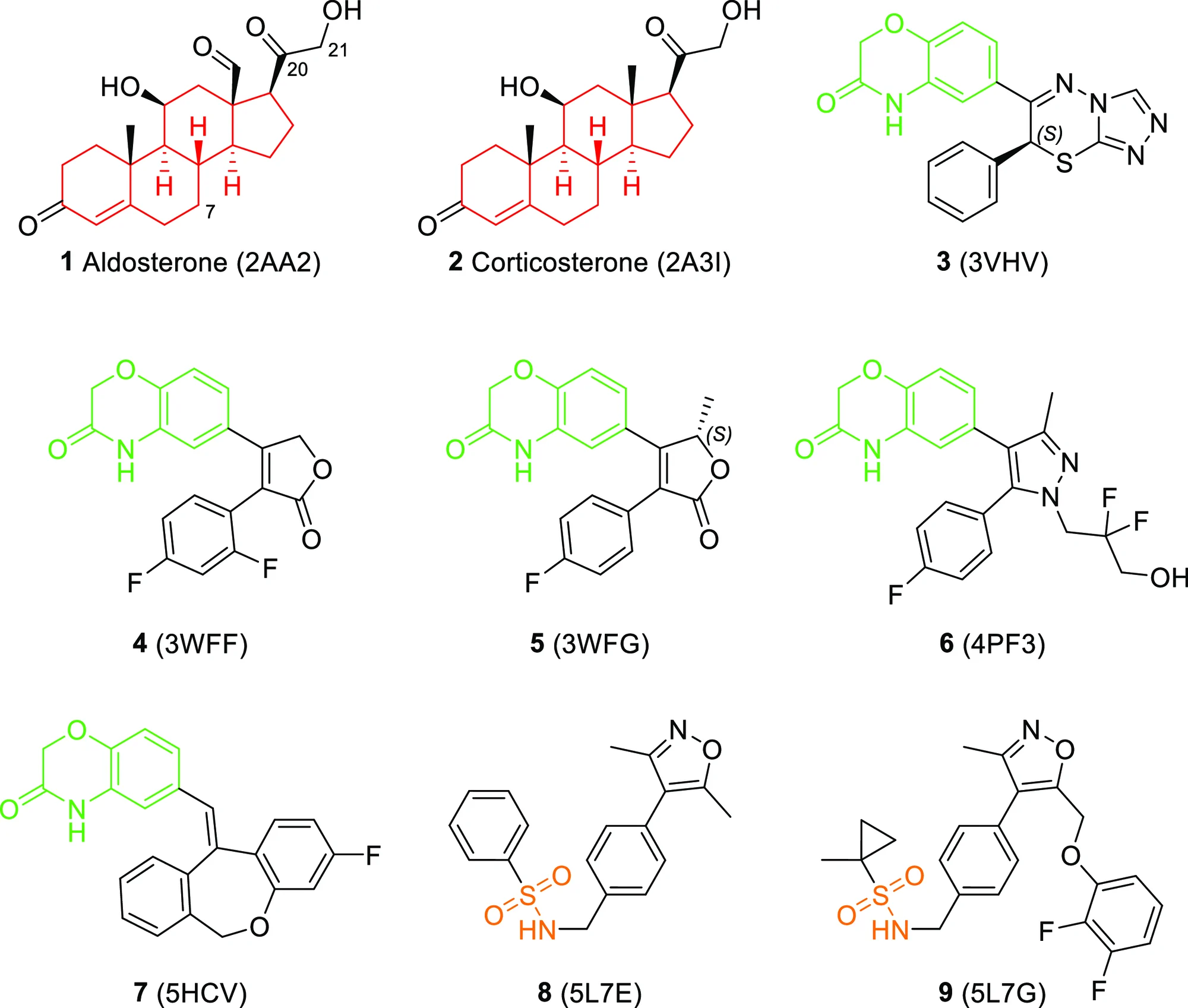
Ligands present in the X-ray MR structures used as templates for IFD studies. In parenthesis the PDB code.^14,16,21–25^

### 3.1 1,4-Dihydropyridines as MR Ligands

The 1,4-Dihydropyridine (DHP) ring has proven to be an effective scaffold in the search for MR ligands, leading to a wide array of DHP derivatives. The relevance of this scaffold has prompted previous molecular docking studies.^34–36^ Here, we expanded upon these studies by examining a broader set of DHPs, offering deeper insights into the role of the DHP substituents in modulating MR binding affinity and receptor selectivity. As shown in Table 1, most compounds were assayed as racemic mixtures. However, in cases where enantiomers have been isolated, those with the 4R stereochemistry exhibit higher affinity. Accordingly, unless otherwise indicated, the IFD results presented here refer to the 4R enantiomer.

**Table 1:**
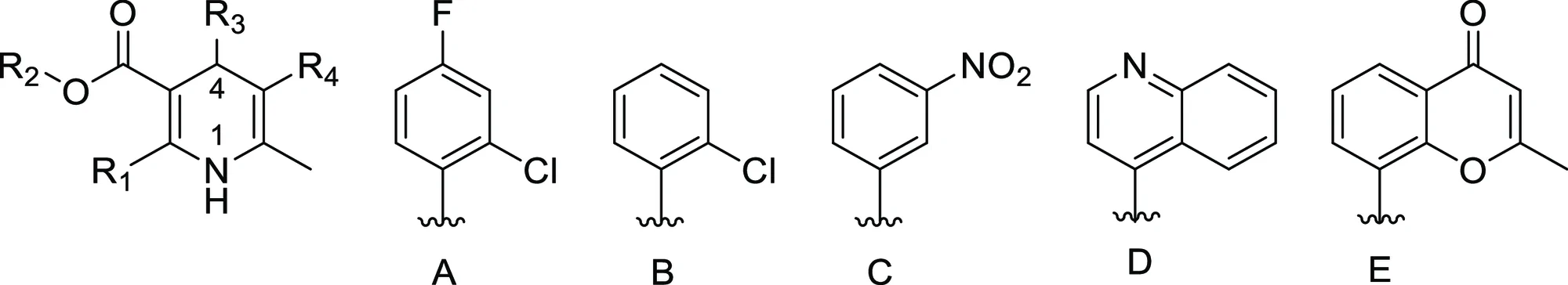

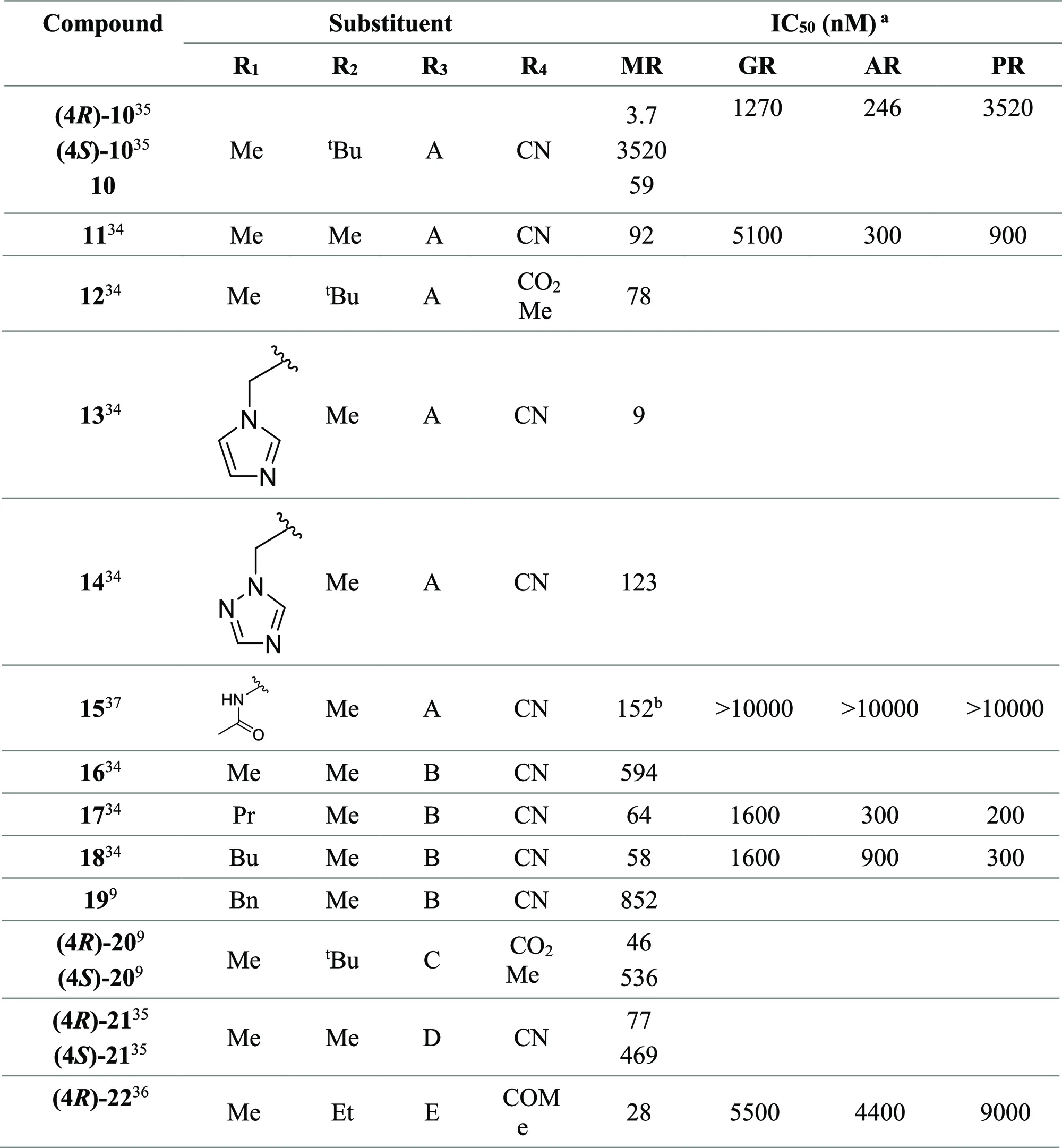
Binding Affinity of Selected 1,4-DHPs for MR and Other Steroid Receptors^a^ ^,^ ^b^

a Unless otherwise indicated, data refer to racemic mixtures.

b *K*_i_ value.

Our IFD studies with the selected DHPs revealed a binding mode consistent with previous studies.^33–35^ Briefly, the aryl group at position four of the DHP ring occupies the region of the steroid A ring and is surrounded by hydrophobic residues Leu769, Leu772, Leu814, Met852, and Phe829, with the latter participating in a T-shaped stacking interaction (Figure 2A). In addition, the 1,4-DHP scaffold shows partial overlap with the corticosterone C ring (**2**, Figure 1). Regarding polar interactions, the DHP amine group forms a hydrogen bond with the side chain of Asn770, consistent with SAR studies that highlighted the importance of this functional group.^34,36^ Additionally, the substituent at position 5 (R_4_, Table 1) may establish a hydrogen bond with Ser810, further contributing to binding stability.

**Figure 2:**
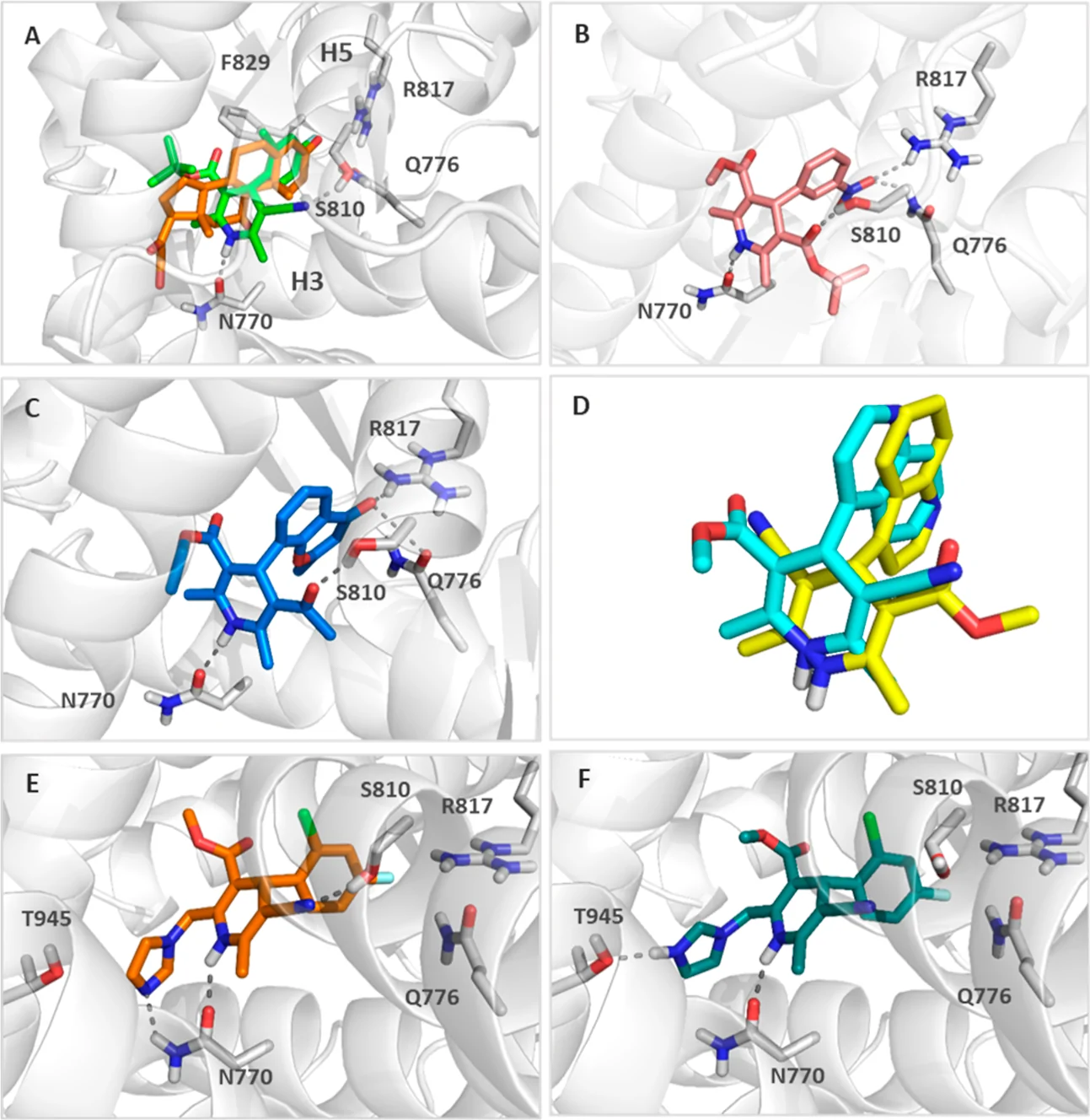
IFD studies on DHP derivatives. Representative IFD poses of MR bound to: (A) DHP **10** (green), superimposed to the MR X-ray structure bound to corticosterone **2** (orange, PDB code 2A3I); (B) DHP **20**; (C) DHP **22**. (D) Superimposition of (4*R*)-**21** (cyan) and (4*S*)-**21** (yellow). (E) DHP **13**, unprotonated imidazole and (F) DHP **13**, protonated imidazole. Hydrogen bonds for DHPs are depicted as gray dotted lines. For clarity, only polar hydrogens are shown.

To assess whether rigid docking could provide suitable binding poses, we carried out Glide docking using the Schrödinger suite of programs. In this protocol, a softened potential is applied by scaling the ligand van der Waals radii to 0.8, allowing a limited tolerance for close contacts while maintaining an overall rigid docking framework for the protein. As representative DHPs, compounds **10**, **12**, **13**, **20**, **21,** and **22** (all with 4R stereochemistry) were selected, and we used the same template as in the IFD studies (PDB code 2A3I). Docking poses were obtained exclusively for **10** and **13**, with only **13** yielding a plausible binding mode consistent with the known structure-activity relationships within this family. Notably, **13** has a small R_2_ substituent (methyl) and a monocycle group at position 4 (R_3_), suggesting that, even with softened van der Waals radii, rigid docking fails to accommodate bulkier substituents, despite these being present in known MR ligands. Comparison with IFD results reveals a conformational adjustment of Met845 that allows the accommodation of the *tert*-butyl ester group. Therefore, given the limitations observed for rigid docking, the IFD approach was used for the remaining compound families.

Following the characterization of DHPs’ general binding mode, we investigated the effect of different substituents at position 4 (Table 1, R^3^). Among them, the 2-chloro-4-fluor-phenyl moiety has been extensively explored in the context of MR biological studies, as exemplified by derivatives **10**–**15**. Computational analyses reveal that the fluorine atom on this aryl ring overlaps with the steroid A-ring CO group, which participates in a bifurcated hydrogen bond with MR Arg817 and Gln776 (Figure 2A). Therefore, the fluorine can act as the proton acceptor, potentially explaining the increased affinity of derivatives fluorinated at position four (see compounds **11** vs **16**, Table 1). IFD studies further showed that the incorporation of a 3-nitrophenyl group, as in DHP **20** (mebudipine), led to a slight displacement of the aromatic ring, enabling the formation of two hydrogen bonds between the NO_2_ moiety and the Arg817 and Gln776 residues (Figure 2B). Regarding derivatives with bicyclic scaffolds, quinolone **21** and chromenone **22**, only the latter established a bifurcated hydrogen bond with Arg817 or Gln776 through the chromenone CO group (Figure 2C). The binding mode of **22** is in agreement with a previous docking study performed in the absence of MR helix H12 that was supported by mutagenesis data.^33^

An interesting question concerns the higher affinity of the DHP 4R enantiomers and the wide range of affinity differences between enantiomers, spanning 1 to 3 orders of magnitude depending on the substituents, as observed in compounds **10, 20,** and **21** (Table 1). While these enantioselectivity trends are well documented at the experimental level, as far as we know, the structural basis underlying these differences had not been previously rationalized. To address this, we analyzed the binding modes of both enantiomers using IFD studies. These studies revealed that the ring at position 4 of both enantiomers adopts similar orientation, mimicking the spatial disposition of the steroid A ring. This overlap arises from a swap between the CN and CO_2_Me groups (Figure 2D). However, a 180° flip is prevented for bulkier esters such as CO_2_^*t*^Bu in **10** and **20**. Therefore, the resulting decrease in affinity between enantiomers may be attributed to a reduced complementarity of these groups within the exchanged binding pockets.

Regarding the R^4^ substituent, both the nitrile group in **10** and the methyl ester in **12** are suitable groups (Table 1). In agreement, our IFD analysis showed a good superimposition between the binding poses of these two derivatives, both being able to establish hydrogen bonds with Ser810.

Analysis of the substituent at position 2 of 1,4-DHP showed that it fits within a pocket surrounded by Leu766, Phe941, and Val954. Increasing chain length from methyl (**16**) to propyl (**17**) and butyl (**18**) resulted in enhanced affinity, likely due to increased hydrophobic interactions. In contrast, a bulkier moiety such as CH_2_-Ph of **19** reduces affinity, probably due to steric clashes within the binding pocket. These substituents overlap with aldosterone (**1**, Figure 1) C20 and C21 but, unlike aldosterone, cannot establish hydrogen bonds with Asn770 or Thr945. Thus, our IFD studies suggest that introducing polar groups at this position could improve interactions with MR, as observed with DHP **13**, which forms an additional hydrogen bond with Asn770 compared to compound **11** (Figure 2E).^34,37^ However, derivatives **14** and **15** showed a slight decrease in affinity. Notably, the imidazole-containing compound **13** displayed nearly 14-fold higher affinity than the triazole-containing **14**. Our IFD indicated that the protonated imidazole NH group can form a H-bond with Thr945, mimicking the steroid 20–ketone interaction (Figure 2F). Since the equivalent conjugate acid is not favored for either 1,2,4-triazol (**14**) or tetrazol rings, this protonation might play a role in the binding of **13** and its higher affinity. Supporting this, previous IFD studies of derivative **15**, which also displayed lower activity than **13**, showed the absence of a hydrogen bond with Thr945.^34^ Overall, these IFD studies highlight a delicate balance between the size and polarity of substituents at position 2 and their influence on affinity, emphasizing the potential interest of introducing hydrogen-bond donors capable of interacting with Thr945.

A key feature of DHP derivatives is their selectivity for MR over other steroid receptors (Table 1). Consistent with prior studies,^33,35^ this selectivity can be attributed, at least in part, to the ability of most DHPs to form hydrogen bonds with Ser810 and their proximity to Ala773. Notably, both Ser810 and Ala773 are unique to MR, corresponding to Met or Gly, respectively, in GR, AR, and PR.^38,39^

Regarding the agonist or antagonist nature of MR ligands, our observations align with earlier findings, indicating that MR antagonists weaken the characteristic hydrogen-bond network typically associated with agonist binding. This weakening has been linked to a passive mechanism of antagonism, similar to that of spironolactone.^16,40,41^ In particular, DHPs form a single hydrogen bond with Asn770 and, in general, none with Thr945. In contrast, in this pocket, the agonist, aldosterone, forms two hydrogen bonds with Asn770 and two with Thr945 (PDB code 2AA2).^14^

It has also been proposed that certain DHPs may act as bulky antagonists due to steric hindrance from substituents at positions 5 and 6 of the 1,4-DHP ring, potentially preventing helix H12 from adopting the required disposition for coactivator recruitment.^33,35^ To explore this hypothesis, and better delineate the binding site, we performed molecular dynamics simulations of MR in complex with 1,4-DHP **12** and **20**, both bearing bulky substituents at R_2_ (Table 1). Each complex was solvated with water molecules (TIP3P) and subjected to a 1000 ns simulation using the Amber software, with the FF19SB force field for the protein and GAFF for the ligand. The simulation was conducted at 300 K using the Langevin thermostat, and three independent replicas were performed for each compound to assess reproducibility. Across all MD systems, protein and ligand RMSD and total energy exhibited comparable distributions among replicas (Supporting Information, Figures S1 and S2), indicating consistent sampling and supporting the robustness of the simulations. Analysis of the MD simulations revealed a slight displacement of the ligands within the binding pocket compared to their IFD pose, with the greatest variation observed for the ^*t*^Bu group (Figure 3A,B). Regarding hydrogen bonds, both derivatives retain one with Asn770 throughout the 1000 ns simulation, with **20** maintaining it over 99% of the time and **12** ranging from 63% to 95%, depending on the replica. However, only compound **12** consistently maintained a hydrogen bond with Ser810 in over 79% of the trajectory. Therefore, despite exhibiting similar binding modes, variations in phenyl ring substituents lead to subtle yet meaningful differences in the hydrogen-bond pattern.

**Figure 3:**
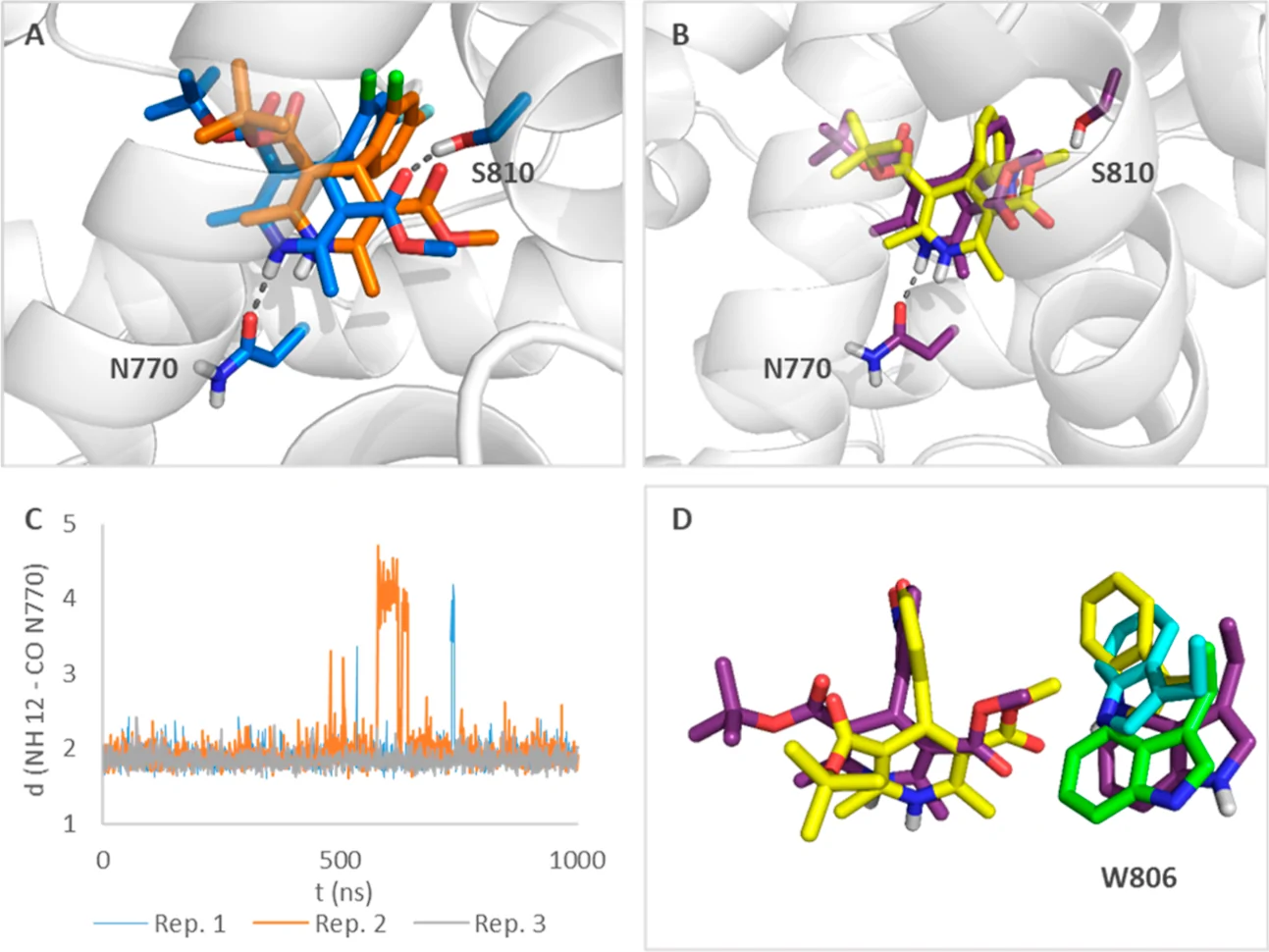
MD studies on DHP derivatives. (A) Superimposition of a 1000 ns frame of **12** (blue) and the corresponding IFD pose prior to MD (orange). (B) Superimposition of a 1000 ns frame of **20** (purple) and the corresponding IFD pose prior to MD (yellow). (C) Distance along the MD of DHP **12** NH proton and the MR Asn770 side chain CO. (D) Superimposition of MR complex with **20** prior to MD (yellow) and a 1000 ns frame (purple); MR complex with aldosterone (2AA2, green, only Trp806 shown) and with esaxerenone (6L88, cyan, only Trp806 is shown). For clarity, only polar hydrogens are shown. Hydrogen bonds are depicted as dotted gray lines and correspond to the 1000 ns MD frames.

Previous IFD studies suggested that a shift in the side chain of Trp806 could create steric clashes with helix H12.^33,35^ Interestingly, the resolution of the MR structure in complex with esaxerenone (PDB code 6L88),^42^ the only known MR structure showing a displacement of helix H12, revealed a different orientation of Trp806 side chain. A close analysis of our IFD results showed that compound **20** positions Trp806 similar to that seen in the esaxerenone-bound structure 6L88 (Figure 3D), whereas in complex with **12**, Trp806 adopts a conformation consistent with the other MR structures, such as 2AA2. However, during the 1000 ns of the MD simulations with derivative **20**, the dihedral angles of Trp806 side chain shift toward those observed in 2AA2 (Figure 3D). This finding suggests that the alternative Trp806 orientation seen in IFD studies may result from limitations of docking approaches in accounting for protein backbone flexibility. These observations underscore the importance of complementing docking with MD simulations to capture more realistic conformational dynamics.

Building on the 1,4-DHP scaffold, a series of heterobicyclic analogues have been developed, most notably finerenone, **23** (Figure 4A), which has received clinical approval under the trade name Kerendia. It is worth noting that its design was guided by docking studies.^36^ Our IFD studies showed a finerenone binding mode consistent with a previously proposed docking model involving MR without helix H12.^36^ Briefly, the naphthyridine ring overlaps with the C and D rings of aldosterone (**1**, Figure 1), while the phenyl ring superimposes with the A ring, and the nitrile group mimics the 3-oxo group of steroids, interacting with Gln776 and Arg817 (Figure 4B). Furthermore, the naphthyridine NH and 3-CONH_2_ groups form hydrogen bonds with residues Asn770 and Ser810, respectively.

**Figure 4:**
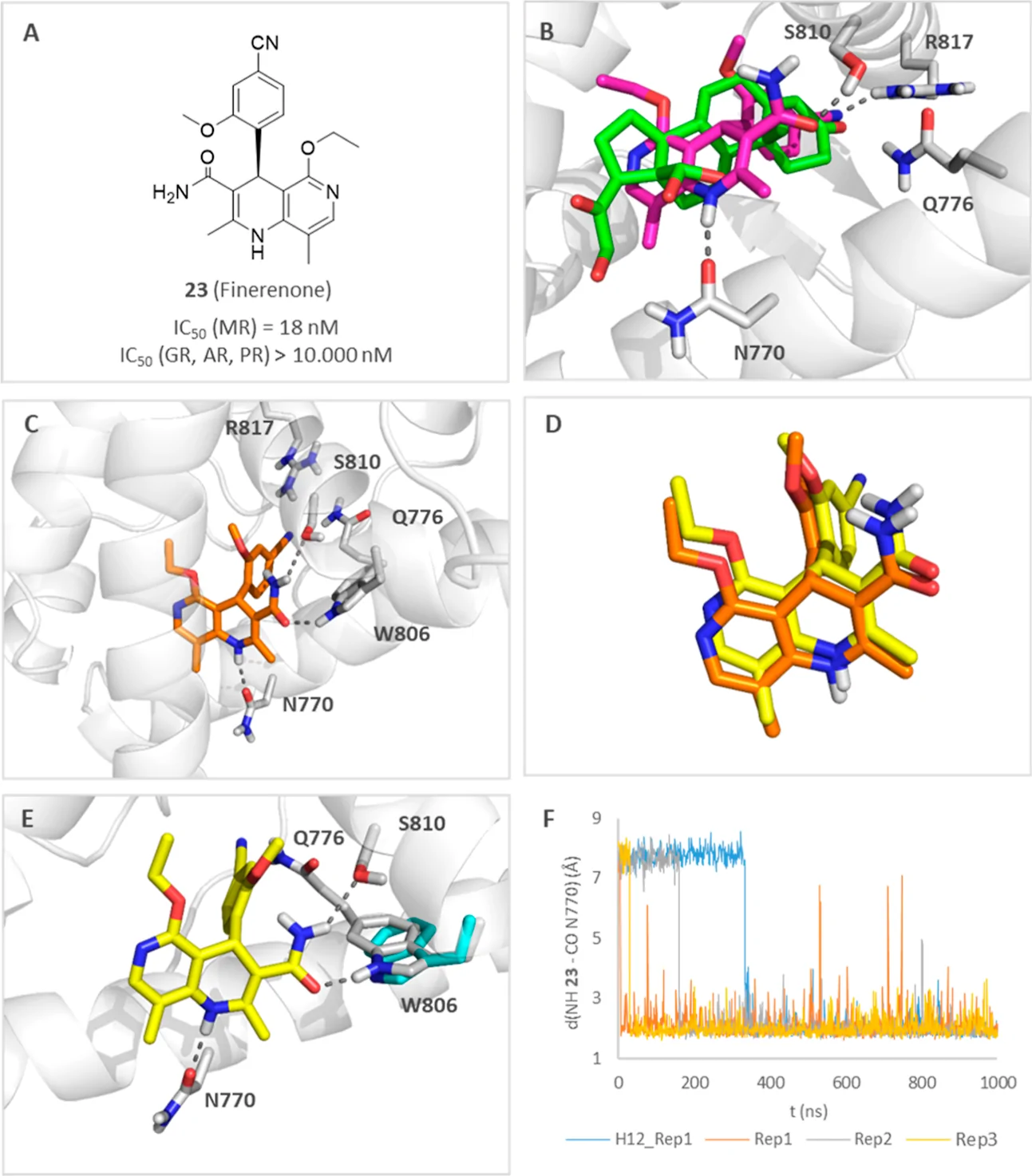
IFD and MD studies on finerenone. (A) Structure of finerenone. (B) Superimposition of the IFD complex MR bound to finerenone **23** (pink), with the X-ray structures of aldosterone bound to MR (green, PDB code 2AA2). (C) Representative 1000 ns MD frame of **23** (orange) in the presence of MR helix H12. (D) Superimposition of the representative 1000 ns MD frame of compound **23** simulation with MR H12 (orange) and without H12 (yellow). (E) Representative 1000 ns MD frame of **23** without MR H12 (yellow), superimposed on the X-ray structure of MR bound to esaxerenone (cyan, PDB 6L88, only Trp806 shown). (F). Distance along the MD simulation between **23** CONH_2_ oxygen and the Trp806 side chain NH, with MR H12 (blue line) and without H12 (orange, gray, and yellow lines). Hydrogen bonds for derivative **23** are depicted as gray dotted lines. For clarity, only polar hydrogens are shown.

To further characterize the binding of finerenone to MR, we conducted three independent 1000 ns MD replicas following the same protocol used for DHPs. Consistent structural and energetic behavior was observed across replicas (Supporting Information Figures S1 and S2), supporting the robustness of the simulations. The simulations revealed only subtle movements of the ligand within the binding pocket. The hydrogen bond with Asn770 was consistently maintained throughout the simulations, while CONH_2_ remained within 2.5 Å of Gln776 and Ser810 in over 63% and less than 40% of the frames, respectively (Figure 4C). As observed in the MD studies with DHP **12** and **20**, compound **23** does not appear to interfere with helix H12 during the 1000 ns simulation, although this time scale may be insufficient to detect potential helix H12 movements. To explore this further, we carried out additional MD studies with helix H12 removed prior to the simulation. Compound **23** maintained its localization and conformation within the binding pocket across all three replicas, regardless of the presence or absence of helix H12 (Figure 4D). The simulations without helix H12 indicated that the ligand can establish hydrogen bonds with Asn770 and Gln776 in over 85% and 50% of the frames, respectively. Most notably, the dihedral angle of Trp806 changed, allowing its indole NH to form a hydrogen bond with the oxygen of the finerenone amide side chain, in over 80% of the simulation. This interaction, absent in the initial structure, appeared after 10–300 ns of the simulation and mirrors the Trp806 conformation observed in the esaxerenone-bound MR structure (PDB: 6L88), the only known structure where helix H12 adopts an antagonist-like conformation (Figure 4E,F). In contrast, in the other known MR structures such as 2AA2, Trp806 forms a hydrogen bond with Gln967 on helix H12, likely stabilizing the packing of H12 against helices H3, H5, and H11. Interestingly, in one of the MD replicas including helix H12, both finerenone **23** and DHP **20** formed a hydrogen bond with Trp806 for over 50% of the simulation time, although in the case of DHP **20**, this interaction was lost and Trp806 reverted to the conformation seen in 2AA2 (Figure 3D). Altogether, these MD simulations raise an interesting point, suggesting that finerenone’s ability to establish a hydrogen bond with Trp806 may weaken the packing of helix H12, potentially destabilizing the AF-2 groove responsible for coregulator recognition. This mechanism may also apply to other DHP derivatives, contributing to their antagonist activity. It is interesting noting that, although studies are limited, different coregulator recruitment profiles have already been reported for MR ligands, including eplerenone, spironolactone, finerenone (**23**), and balcinrenone (**28**).^43,44^ Interestingly, **23** exhibits stronger inhibition of coregulator binding than eplerenone, whereas **28**, unable to form a hydrogen bond with Trp806, shows weaker inhibition than eplerenone.

### 3.2 MR Ligands with a 1,4-Benzoxazinon-3-one Ring and Related Families

The 1,4-benzoxazin-3-one moiety has been thoroughly explored in the search of MR ligands, and to date, the majority of X-ray ligand-bound structures correspond to this family, with PDB codes 5MWP (**24**),^45^ 3VHV (**3**),^21^ 3WFF (**4**), 3WFG (**5**),^22^ 4PF3 (**6**),^23^ 5HCV (**7**),^24^ 6GEV, 6GG8, and 6GGG.^46^ These ligands, e.g., **5** and **6** (Figure 1, Table 2), share common motifs, such as a benzoxazinone and phenyl ring, attached to two adjacent positions of a central heterocycle. These 3D structures have provided insights into the binding mode of this family. This includes the presence of a hydrogen bond between the cis-amide moiety of the benzoxazinone ring and MR residues Asn770 and Thr945, with the positioning of the phenyl ring within a hydrophobic pocket defined by Met845, Met852 and Leu938, as well as the involvement of the central heterocycle in hydrogen bonds with residues Gln776 and Arg817, either directly or mediated by water molecules (Figure 5). Within this family, the most well-known member is balcinrenone (**24**), currently undergoing clinical evaluation in phases IIb and III.^47^ Unlike the analogues described above, this molecule lacks a cyclic spacer but maintains the three-bond distance between benzoxazinone and the phenyl ring. Accordingly, its 3D structure bound to MR (PDB code 5MWP) revealed a good superimposition with the X-ray structures of other benzoxazinone derivatives.

**Table 2:**
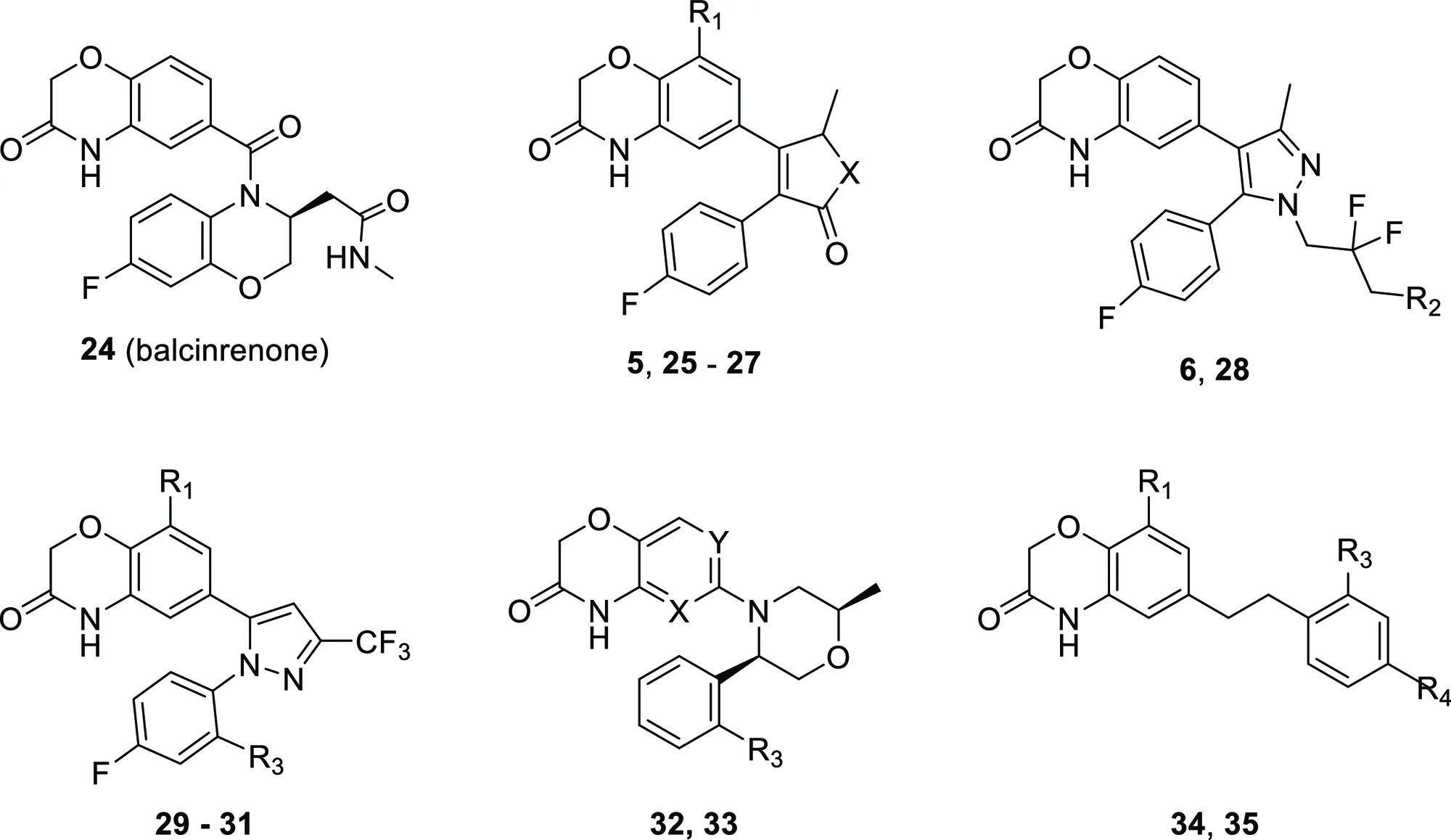
Binding Affinity of Benzoxazinone Derivatives for Steroid Receptors MR, AR, PR, and GR

compd	R_1_	R_2_	R_3_	R_4_	X	Y	IC_50_ (nM)^a^			
							MR	AR	PR	GR
5 ^22^	H		-	-	O	-	120	>10000	740	3800
6 ^23^	-	OH	-	-	-	-	51	>10000	>10000	2400
24 ^45^	-	-	-	-	-	-	370	>10000	>10000	4000
25 ^22^	H	-	-	-	N–Et	-	33	>10000	770	1600
26 ^22^	H	-	-	-	NH	-	94	>10000	5600	9100
27 ^22^	Me	-	-	-	NH	-	43	>10000	>10000	4900
28 ^23^	-	Me	-	-	-	-	5.8	>10000	4000	540
29 ^21^	H	-	Me	-	-	-	41	>10000	1900	1800
30 ^21^	H	-	H	-	-	-	260			
31 ^21^	Me	-	Me	-	-	-	21	>10000	2400	470
32 ^48^	-	-	F	-	N	CH	33.4			
33 ^48^	-	-	F	-	CH	N	53.9			
34 ^49^	Me	-	Me	OAc	-	-	1.3			210
35^2^ ^24^	H	-	F	F	-	-	20			

**Figure 5:**
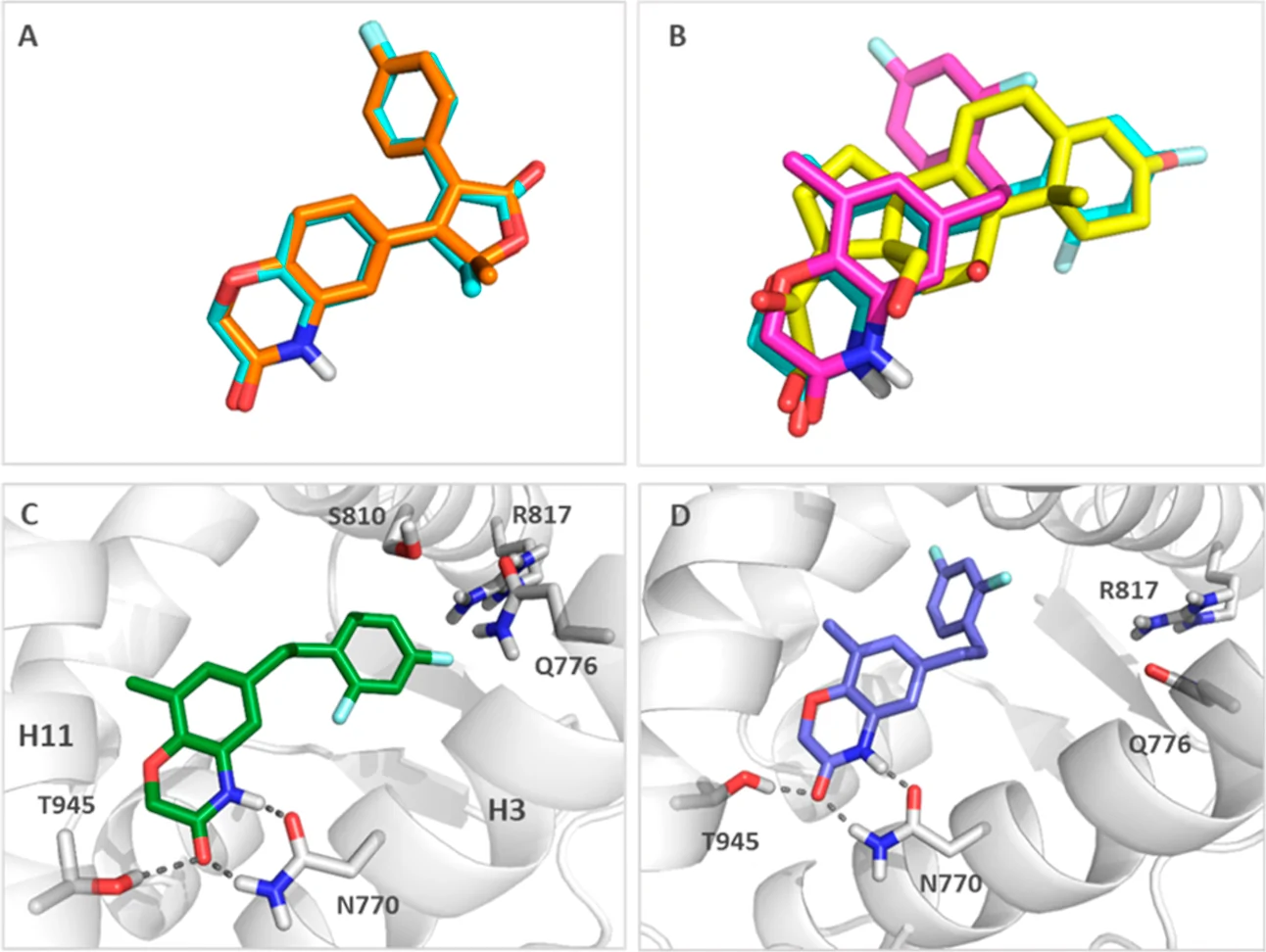
IFD studies on benzoxazinone derivatives. (A) Superimposition of one IFD pose of **5** (cyan) and its disposition in the X-ray structure (orange, PDB code 3WFG). (B) Superimposition of the two IFD poses identified for derivative **35** (cyan, starting with the 2A3I structure), **35** (magenta, starting with the 3WFG structure), and aldosterone (**1**, yellow, PDB 2AA2). (C) Representative IFD pose of MR—compound **35** (green) complex using 2A3I as a template. (D) Representative IFD pose of MR—compound **35** (blue) complex using 3WFG as a template. Hydrogen bonds are depicted as gray dotted lines. For clarity, only polar hydrogens are shown.

While this compound family has been extensively studied, several questions remain unanswered. For instance, it is not known whether derivatives with linear linkers adopt a similar binding mode or what role Ser810, a residue unique to MR among steroid receptors, plays in ligand interaction. Notably, most available X-ray structures for this family, except 5MWP and 6GG8, incorporate the S810L mutation. Furthermore, most of these structures, especially the mutated ones, contain water molecules that are crucial for establishing a hydrogen-bond network between the ligands and MR residues Gln776/Arg817. This network has been implicated in enhancing the agonist properties of several ligands.^23^ To address these questions, we generated models based on PDB structures 3WFG and 4PF3 in which Leu810 was reverted to the WT Ser810, with or without the inclusion of water molecules. For the IFD studies, we selected a variety of benzoxazinone derivatives, including those with cyclic spacers containing furane (**5**), pyrrol (**25**–**27**), pyrazol (**6**, **28**–**31**), and morpholine (**32**, **33**), as well as linear spacers such as ethane (**34**, **35**) (Table 2). Among these, two derivatives **5** and **6** have experimentally determined MR-bound 3D structures available (PDB codes 3WFG and 4PF3, respectively) and were used as control to verify the accuracy of the IFD predictions.

The IFD studies consistently showed that the top-ranked poses, regardless of the nature of the linker, situated the benzoxazinone ring similar to its arrangement in the known X-ray structures. Therefore, the cis-lactam moiety is involved in hydrogen bonds with Asn770/Thr945. Notably, the predicted binding mode of **5** and **6** provided by our IFD studies closely matched their experimentally determined 3D structures in complex with MR (PDB codes 3WFG and 4PF3, respectively), thereby validating the IFD protocol used in our study (Figure 5A).

It is worth noting that our IFD studies revealed two alternative binding poses for derivatives featuring linear linkers such as **34** and **35**, depending on the MR structure used as the docking template (Figure 5B). When simulations were based on the structure of PDB code 3WFG, the phenyl ring adopts a disposition similar to that observed for the derivatives with cyclic linkers. In contrast, when the 2A3I structure was used as a template, the phenyl ring occupies a different pocket, overlapping with the A ring of aldosterone (PDB ID 2AA2) (Figure 5B). Regardless of the binding mode adopted by the phenyl ring, the cis-amide moiety consistently forms a bidentate hydrogen bond with Asn770 and an additional interaction with Thr945 (Figure 5C,D).

To evaluate the most plausible binding mode, MD studies were carried out starting from the two identified IFD poses for compound **35**. In all MD systems, protein and ligand RMSDs and total energies were comparable across replicas (Supporting Information, Figures S3 and S4), indicating consistent sampling. For simulations initiated from the 2A3I-based pose, which displayed an unexpected orientation of the phenyl ring (Figure 5C), all three independent 1000 ns MD replicas demonstrated that the MR–compound **35** complex remains stable over time. Only minor deviations from the initial IFD pose were observed for the linear spacer, while the aromatic rings exhibited a high degree of superimposition throughout the trajectory (Figure 6A). Furthermore, hydrogen-bonding distances between the benzoxazinone cis-amide and the Asn770 side chain CO and NH_2_ were maintained for more than 95% and 70% of the simulation, respectively (Figure 6B, Supporting Information Figure S5A,B). In this binding mode, the acetyl or fluorine substituent at position 4 of the phenyl ring is oriented toward Gln776 and Arg817, enabling it to mimic the interaction pattern of the 3-oxo group of aldosterone (Figure 6C). On the other hand, MD studies initiated from the 3WFG-derived pose also showed a stable bidentate interaction between the cis-amide moiety of **35** and the Asn770 side chain (Supporting Information Figure S5C–E). However, the MR–**35** complexes remained stable in only two of the three 1000 ns replicas. In the remaining replica, a conformational rearrangement occurs at approximately 700 ns, involving a change in the linker dihedral angle leading to a phenyl ring orientation similar to that observed in the 2A3I-derived pose (Figure 6D, Supporting Information Figures S4D and S5F). Interestingly, although the initial IFD poses generated using either the 2A3I or 3WFG templates indicated the presence of a hydrogen bond between compound **35** and Thr945, this interaction is not maintained during the MD trajectory. Instead, a change of Thr945 side-chain dihedral angle is observed, leading to the formation of a hydrogen bond with the backbone carbonyl of Phe941 for almost the entire trajectory (98%) (Supporting Information Figure S5G,H). Notably, a similar interaction has been reported for progesterone binding, where the authors proposed a potential competition of this hydrogen bond either with the backbone carbonyl of Cys942 or with an intermolecular hydrogen bond involving the ligand.^14^

**Figure 6:**
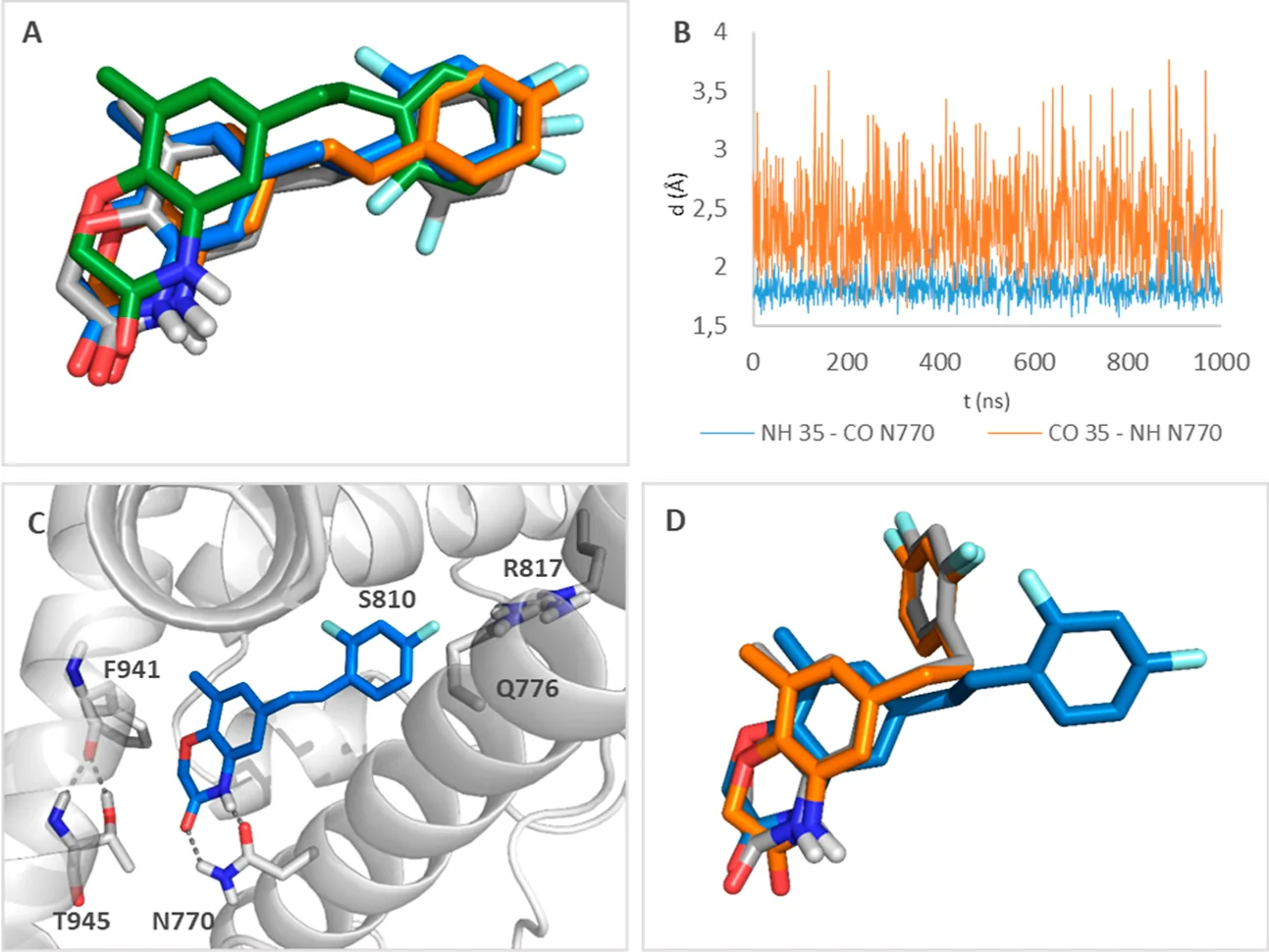
MD studies on benzoxazinone derivatives. (A) Superimposition of MR—compound **35** complexes: the initial IFD pose (green) and the final 1000 ns frames from the three MD replicas (blue, orange, gray). (B) Time evolution of the distances, for one representative replica, between the cis-amide moiety of compound **35** and Asp770 side chain during the MD of the MR—**35** complex, initiated from the 2A3I-derived pose. (C) Representative 1000 ns frame of the MR—**35** complex obtained from MD simulations initiated from the 2A3I pose. (D) Superimposition of the final 1000 ns frames of the MR—**35** complexes from the three MD replicas initiated from the 3WFG pose (orange, gray, and blue). Hydrogen bonds are depicted as gray dotted lines. For clarity, only polar hydrogens are shown.

Although neither IFD nor MD simulations enable an unambiguous assignment of the preferred binding mode of linear benzoxazinones, it is tempting to consider the conformation derived from 2A3I as more stable, as one of the replicas initiated from 3WFG evolved toward this arrangement during the MD simulation. Alternatively, it also raises the possibility that derivatives with a linear spacer, owing to their greater flexibility compared with cyclic linkers, may adopt these two binding modes within the MR binding pocket.

Regarding Ser810, as stated above, we performed IFD studies on derivatives **6** and **26** using the 3WFG MR model in which the Leu810 residue was reverted to the wild-type Ser. These simulations revealed binding modes consistent with those previously described. In the case of derivative **6**, IFD studies suggest the formation of a hydrogen bond between the compound **6** hydroxyl group and Ser810 (Figure 7A). However, subsequent MD simulations did not support the persistence of this interaction, as the Ser810 hydroxyl group preferentially establishes a hydrogen bond with the Trp806 backbone carbonyl for over 75% of the trajectory (Figure 7B,C). This interaction mimics the hydrogen bond observed in the crystal structures of PDB codes 2A3I and 5MWP. In contrast, hydrogen bonds between compound **6** and the Asn770 side chain carbonyl and amino groups were maintained for more than 95% and 65% of the simulation time, respectively, while the hydrogen bond with Gln776 was observed for over 40% of the trajectory (Supporting Information Figure S6A–C). It is worth pointing out that the hydrogen bond with Thr945 observed in the X-ray structure is present in less than 10% of the simulation time. Instead, similar to what was observed for the complex with compound **35**, a hydrogen bond between Thr945 and the backbone carbonyl of Phe941 is present in around 88% of the simulation time due to a change in the Thr945 dihedral angle (Figure 7C). The consistency of the protein and ligand RMSD values, together with stable total energy profiles across the replicas, supports the reproducibility of the MD simulations (Supporting Information Figures S3,S4)

**Figure 7:**
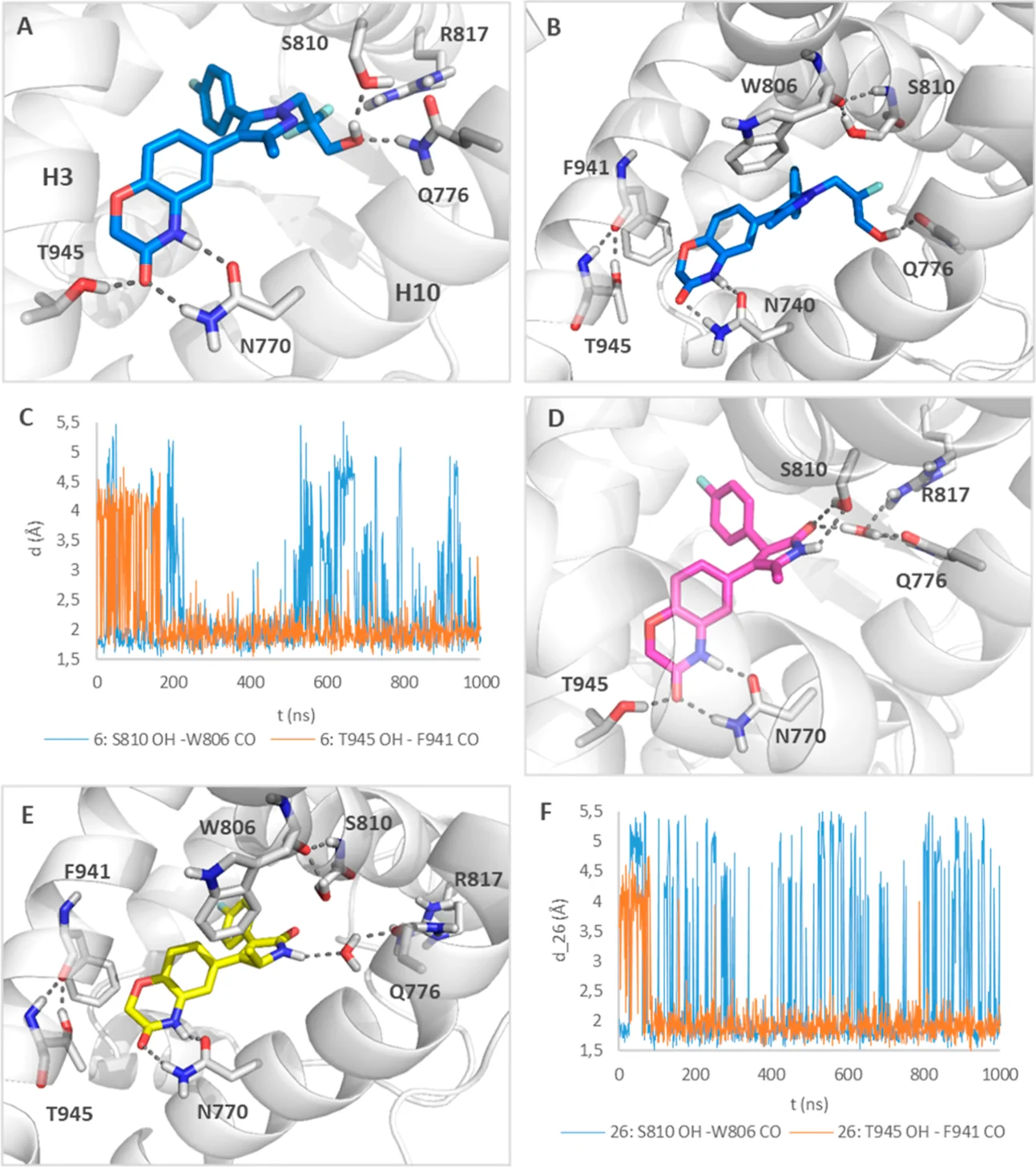
IFD and MD studies on benzoxazinone derivatives 6 and 26. (A) Representative IFD pose of the MR—compound **6** complex. (B) Representative 1000 ns frame of the MR—compound **6** complex obtained from MD simulations. (C) Time evolution of the distances between the hydroxyl hydrogen of Ser810 and the backbone carbonyl oxygen of Trp806 and that between the hydroxyl hydrogen of Thr945 and the backbone carbonyl oxygen of Phe941 during the representative MD replicas of the MR—compound **6** complex. (D) Representative IFD pose of the MR—compound **26** complex. (E) Representative 1000 ns frame of the MR—compound **26** complex obtained from MD studies. (F) Time evolution of the distances between the hydroxyl hydrogen of Ser810 and the backbone carbonyl oxygen of Trp806 and that between the hydroxyl hydrogen of Thr945 and the backbone carbonyl oxygen of Phe941 during the representative MD replicas of the MR—compound **26** complex. Hydrogen bonds are depicted as gray dotted lines. For clarity, only polar hydrogens are shown.

As for derivatives **26** and **27**, our IFD studies suggested that the amino group of their pyrrolidone ring facilitates the formation of an additional hydrogen bond with Ser810 (Figure 7D). However, similar to what was observed in the MD studies of the MR—**6** complex, the simulations of the complex with **26** do not support the stability of this hydrogen bond (Figure 7E). Instead, the hydroxyl group of Ser810 preferentially forms a hydrogen bond with the backbone carbonyl group of Trp806 in more than 70% of the simulation time (Figure 7F and Supporting Information Figure S6D). Furthermore, as observed for the complex with compounds **6** and **35**, the hydrogen bond with Thr945 is not maintained during the simulation and is replaced by an interaction between the Thr945 side chain and the backbone carbonyl of Phe941. This rearrangement is associated with a change in the Thr945 dihedral angle and is present in more than 92% of the simulation (Figure 7F). In contrast, MD studies reveal a stable bidentate hydrogen bond between the cis-amide group of **26** and the Asn770 side chain (Supporting Information S6E,F). The protein and ligand RMSD behavior, together with a stable total energy across replicas, supports the robustness of the MD simulations (Supporting Information Figures S3 and S4)

It is worth noting that the ligand conformation and orientation adopted by derivatives **6, 26,** and **35** after 1000 ns of MD simulations remain highly similar to their respective starting IFD poses. The main differences arise from the hydrogen-bonding pattern, which MD studies reveal is not maintained throughout the trajectories. In particular, the hydrogen bond between compounds **6** and **26** and Ser810 is not persistent, and the Ser810 side chain preferentially interacts with the Trp806 backbone carbonyl, in agreement with the interactions observed in the X-ray structures 2A3I and 5MWP. Similarly, the hydrogen bond observed in the IFD poses between compounds **6**, **26,** and **35** and Thr945 is not retained during the MD simulations and is replaced by an interaction between the Thr945 side chain and the backbone carbonyl of Phe941. It is interesting to note that X-ray structures of MR in complex with several ligands also present a hydrogen bond between the ligand and Thr945; examples include the structures of PDB codes 2A3I, 4PF3, and 3WFG. However, MD simulations indicate that for compounds **6**, **26,** and **35**, this interaction is not stable in solution and that there is a conformational rearrangement that favors a more persistent interaction with the backbone carbonyl of Phe941. This discrepancy reflects the importance of MD simulations in assessing the dynamic stability of ligand–receptor interactions.

Regarding selectivity, it is worth noting that the methyl group on the five-membered heterocyclic ring of compounds **5**, **6**, and **25–28** may contribute to selectivity due to its close proximity to Ala773, a residue unique to MR. Finally, the phenyl ring of these derivatives fits into the same binding pocket as the thioester group of spironolactone (PDB code 3VHU), a cavity that has been suggested to contribute to MR selectivity due to its larger size compared to PR, AR, and GR.^21^

Alongside, there is a series of derivatives structurally related to the benzoxaninone family, such as derivative **36** (Figure 8A), characterized by the presence of a benzoxazinthione ring and a linear sulfonamide group as a linker.^50^ A comparison between our IFD studies for these two families revealed only a partial superimposition of the benzoxazinthione and benzoxazinone rings (Figure 8B). Like the benzoxazinone ring, benzoxazinthione is able to form a hydrogen bond with Asn770 (Figure 8C). Interestingly, despite having a linear spacer similar to that of **34**, derivative **36** positions the thiophene ring occupying the phenyl ring-binding pocket observed for the benzoxazinones with a cyclic spacer (Figure 8B). Furthermore, the sulfonamide group of **36** participates in hydrogen bonds mediated by a water molecule with Gln776 and Arg817 (Figure 8C). On the other hand, IFD studies carried out after reverting the S810L mutation to the native Ser revealed the formation of two hydrogen bonds between the sulfonamide group and Ser810, one of which is mediated by a water molecule (Figure 8C). The selectivity of compound **36** for MR is then likely due to both the thiophene group situated in the selectivity-associated pocket, analogous to the thioacetyl group of spironolactone (PDB code 3VHU), and its capacity to establish hydrogen bonds with Ser810 through the sulfonamide group.

**Figure 8:**
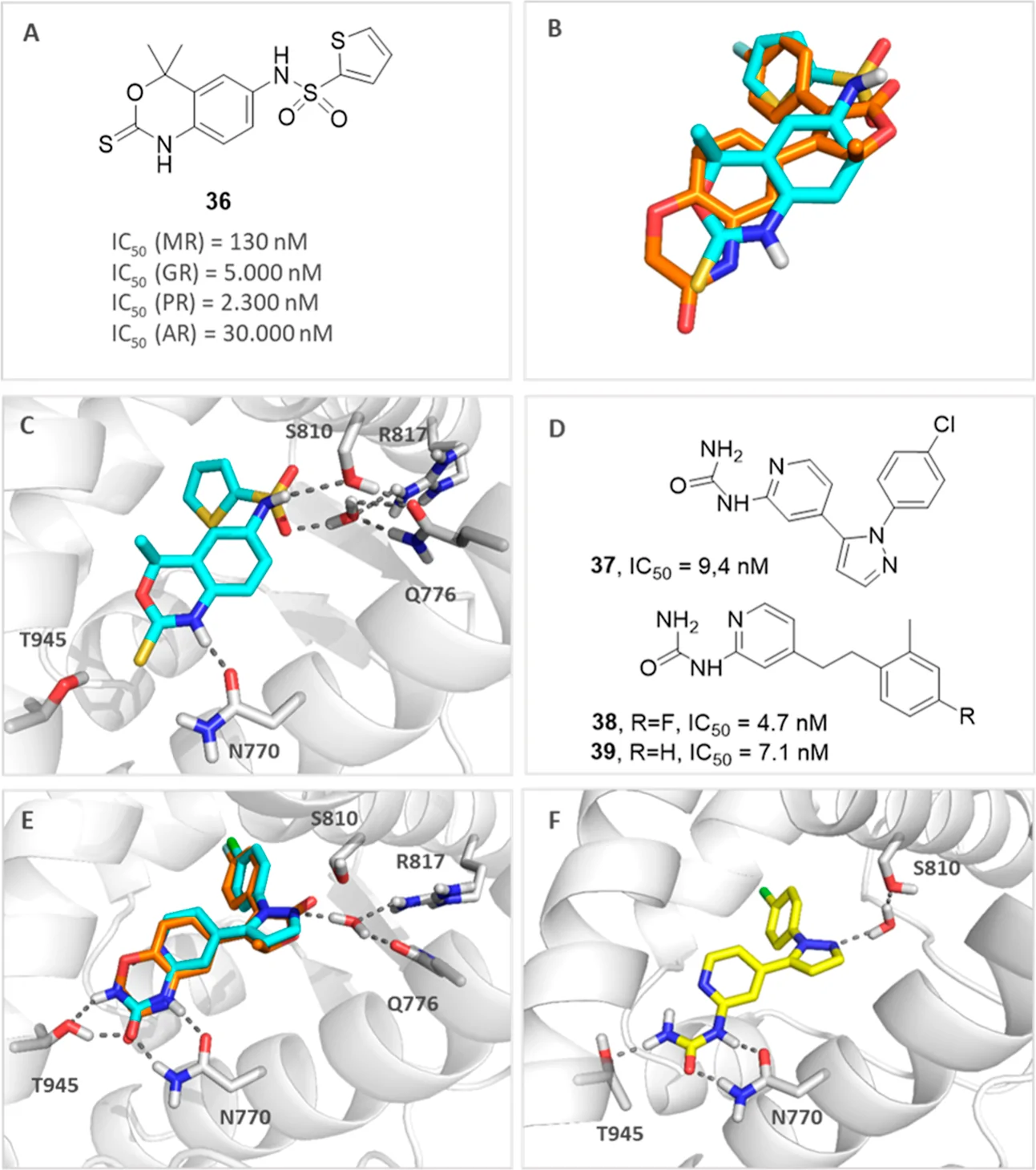
IFD and MD studies on benzoxazinthione and pyridyl urea derivatives. (A) Structure and affinity data of compound **36**. (B) Superimposition of a representative IFD pose of derivative **36** (cyan) with benzoxazinone **5** (orange, PDB code 3WFG). (C) Representative IFP pose of the complex between derivative **36** (cyan) and MR. (D) Structure and affinity data of compounds **37**–**39**. (E) Pyridyl urea **37** (cyan) superimposed to benzoxazinone **5** (orange, PDB code 3WFG). (F) Representative 1000 ns frame of the MR—compound **37** (yellow) complex. Hydrogen bonds for derivatives **36** and **37** are depicted as gray dotted lines. For clarity, only polar hydrogens are shown.

Considering the observed selectivity of benizoxazinone and benzoxazinthione derivatives for MR over other steroid receptors, we further evaluated whether IFD could capture these selectivity differences. As several compounds displayed pronounced experimental selectivity for MR over AR, this receptor provided a stringent test for the ability of IFD to discriminate receptor selectivity. Two AR structures (PDB codes 1T5Z and 3B66) were selected. Given the high structural similarity among steroid hormone receptor ligand-binding domains, assessing selectivity using docking-based approaches remains challenging. Consistently, IFD of compounds **6**, **10**, **15**, **23**, **26,** and **36** identified plausible AR binding poses within the top-ranked solutions, particularly for 1T5Z, involving residues analogous to those observed in MR binding (Supporting Information Table S1, Figure S7). These results suggest that, in this system, IFD alone is insufficient to reliably discriminate subtle energetic and structural differences from docking scores alone. Nevertheless, receptor-specific features, such as nonconserved residues like Ser810, or the presence of a larger subpocket in MR, as discussed above, can still support the rational design of selective ligands.

Our IFD studies also enabled the comparison of pyridyl urea derivatives^51^ (Figure 8D) and the benzoxazinone family, revealing that both types of compounds share a similar binding mode. For instance, the pyridyl urea moiety in compounds **37** shows good superimposition with the benzoxazinone cis-amide group of **5** (Figure 8E). This binding mode is further supported by MD studies, in which the disposition of compound **37** after 1000 ns remains almost identical to the starting IFD pose across three independent replicas (Supporting Information Figure S8A). The reproducibility of these simulations is supported by the comparable distribution of protein and ligand RMSD values, as well as energy, across the three replicas (Supporting Information Figures S9 and S10). Throughout the simulations, a stable bidentate interaction between the urea NHCO group and Asn770 side chain is preserved, while the urea NH_2_ group can participate in an additional hydrogen bond with Thr945 (Figure 8F and Supporting Information Figure S8B–D). Moreover, analysis of the final snapshots at 1000 ns from each replica reveals the formation of a water-mediated hydrogen bond between the compound **37** pyrazol nitrogen and Ser810 (Figure 8F). This interaction, not present in our initial IFD pose, arises from a conformational change in Ser810 χ1 dihedral angle (Supporting Information Figure S8E). Analysis of this dihedral angle along the trajectory showed that it fluctuates between values similar to those of the initial IFD pose (around 170°, −170°) and those observed at 1000 ns (around 60°), with the latter being populated in more than 70% of the trajectory. Considering the role of Ser810 in MR selectivity, this rearrangement may contribute to ligand discrimination against other hormone receptors.

Interestingly, in the case of the pyrazole-containing compound **37**, the phenyl ring overlaps with that of benzoxazinone derivatives, such as **5**, consistent with the presence of a cyclic linker in both series. In this binding mode, the cyclic linker is positioned near Gln776/Arg817, forming water-mediated hydrogen bonds. By contrast, derivatives bearing an ethylene linker (**38** and **39**) orient the phenyl ring toward Gln776 and Arg817, regardless of the IFD template used (2A3I or 3WFG) (Figure 9A,B). This behavior differs from our IFD findings for derivative **35**, in which the phenyl ring binding pocket depends upon the template. In contrast to the phenyl ring, the orientation of the urea moiety in compounds **38** and **39** depends on the template (Figure 9A,B). When the 3WFG structure is used, the urea group shows a good superimposition with the cis-amide moiety of benzoxazinones (Figure 9A), whereas a flipped urea conformation is observed when the 2A3I structure is employed (Figure 9B). Subsequent MD simulations on the MR—compound **39** complex indicate that the latter conformation is unstable, while the former remains stable across three independent 1000 ns replicas, with minimal deviation of the ligand from the initial IFD pose (Supporting Information Figure S8F). These results highlight the importance of sampling multiple receptor conformations in IFD studies, complemented by subsequent MD simulations. Consistent with the derivatives containing cyclic spacers, the bidentate hydrogen bond between the urea and Asn770 is maintained throughout the 1000 ns trajectory along with a hydrogen bond between the urea NH_2_ and Thr945 in more than 80% of the 1000 ns simulation (Figure 9C,D and Supporting Information Figure S8G,H). The robustness of the MD simulations is supported by the comparable distribution of protein and ligand RMSD values, as well as energy, across the three replicas (Supporting Information Figures S9 and S10)

**Figure 9:**
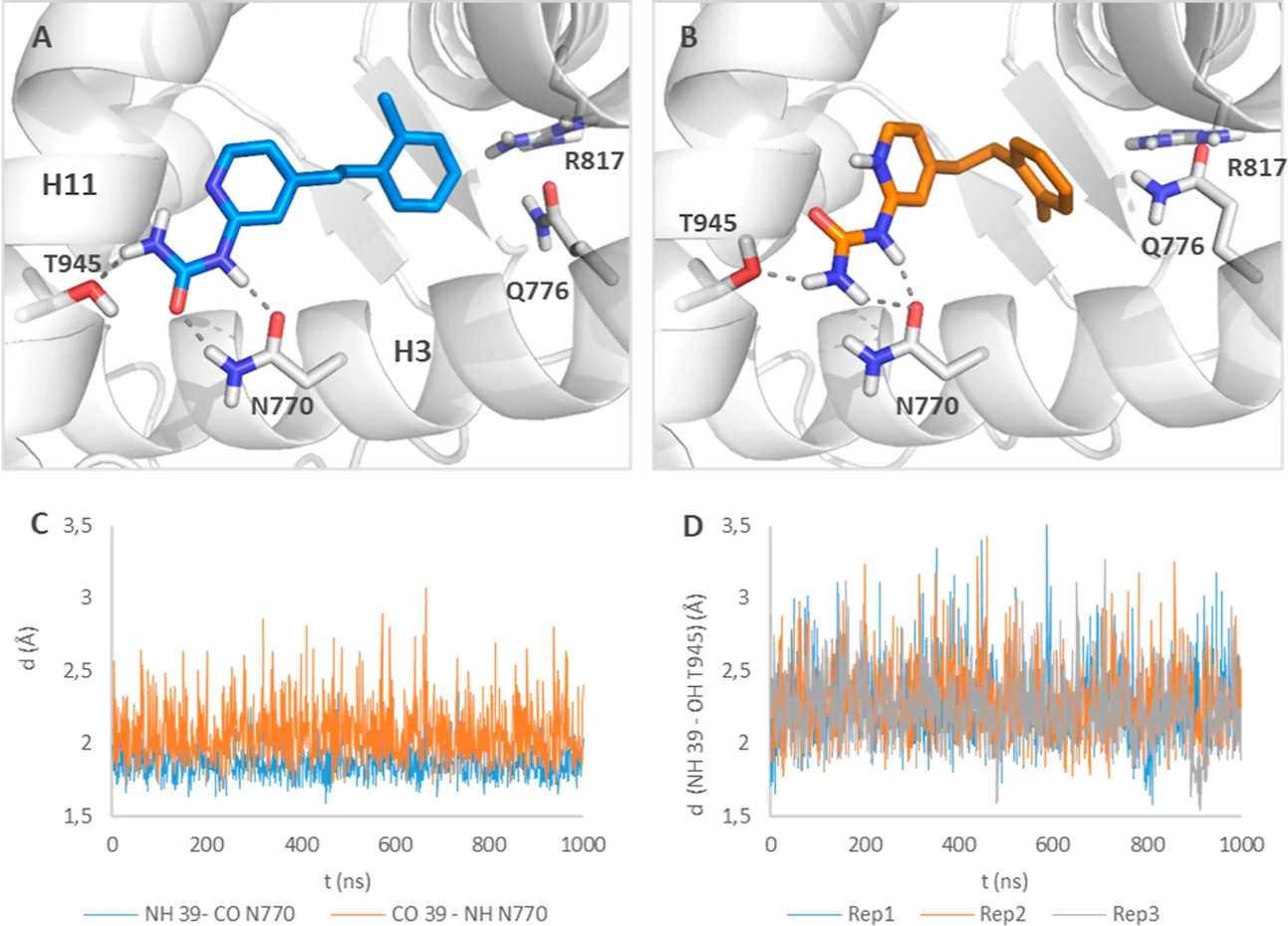
IFD and MD of derivative 39. (A) Representative IFD pose of the MR—**39** complex using 3WFG as the template. (B) Representative IFD pose of the MR—**39** complex using 2A3I as the template. (C) Time evolution of the distances between oxygen and proton of the CONH moiety of compound **39** and the proton and oxygen of N770 CONH_2_, respectively, during a representative MD replica initiated from the 3WFG-derived pose. (D) Time evolution of the distances between the compound **39** NH_2_ proton and the hydroxyl oxygen of Thr945 during the three MD replicas initiated from the 3WFG-derived pose. Hydrogen bonds are depicted as gray dotted lines. For clarity, only polar hydrogens are shown.

It is worth highlighting that pyridyl urea-containing compounds generally show higher affinity than benzoxazinone derivatives. MD simulations reveal that, in contrast to benzoxazinones **6**, **26,** and **35**, pyridyl urea-containing compounds **37** and **39** maintain a hydrogen bond with Thr945 throughout the simulation, which might contribute to their enhanced affinity. This suggests that an effective MR antagonist can be achieved with a molecule containing two aromatic rings connected by a three-bond spacer, which ensures the correct orientation of the groups involved in polar interactions.

### 3.3 MR Ligands with a Dihydrodibenzo­[b,e]­oxepine Tricycle

Among MR ligands, there exists an extensive family characterized by the presence of a fused tricycle motif.^12^ Of particular interest are those compounds with a dihydrodibenzooxepine moiety connected to another aromatic ring via a two-bond spacer.^52–56^ One such compound is derivative **7** (Figure 1, Table 3), which incorporates a benzoxazinone ring. The X-ray structure of **7** in complex with MR (PDB code 5HCV)^24^ shows that the benzoxazinone ring adopts a disposition similar to that observed in the previously discussed benzoxazinone family. Additionally, in this structure, the fluorinated phenyl ring of the tricycle is positioned close to Arg817 and Gln776, while the other aromatic ring occupies the cavity associated with selectivity. Based on that structure (PDB code 5HCV), IFD studies were carried out with a series of **7**-related analogues that maintain the tricyclic scaffold but replace the benzoxazinone ring with either a benzimidazole or indole moiety (Table 3). In agreement with Granberg et al.,^46^ our studies revealed a binding mode similar to that of **7** (PDB code 5HCV),^24^ with the most significant variations observed in the bicyclic ring. Nevertheless, there is a partial overlap of the benzimidazole or indole moieties of compounds **40**–**45** and the benzoxazinone ring of **7** (Figure 10A). These bicyclic rings establish hydrogen bonds with the MR residues Asn770 and Thr945 either via the R_2_ substituent or through the heterocyclic heteroatoms (Figure 10B). For the benzimidazole derivatives, the nitrile or amide moiety at R_2_ can form additional hydrogen bonds with the side chains of Asn770 and Thr945. In contrast, for indole derivatives **43**–**45**, the carbonyl at position 2 of the indole is not at an adequate distance for hydrogen bonding. The lack of these interactions likely explains the lower MR affinity of the indolone derivatives (**43**–**45**), which is 1 to 2 orders of magnitude below that of benzimidazole derivatives (**40**–**42**) (Table 3).

**Table 3:**
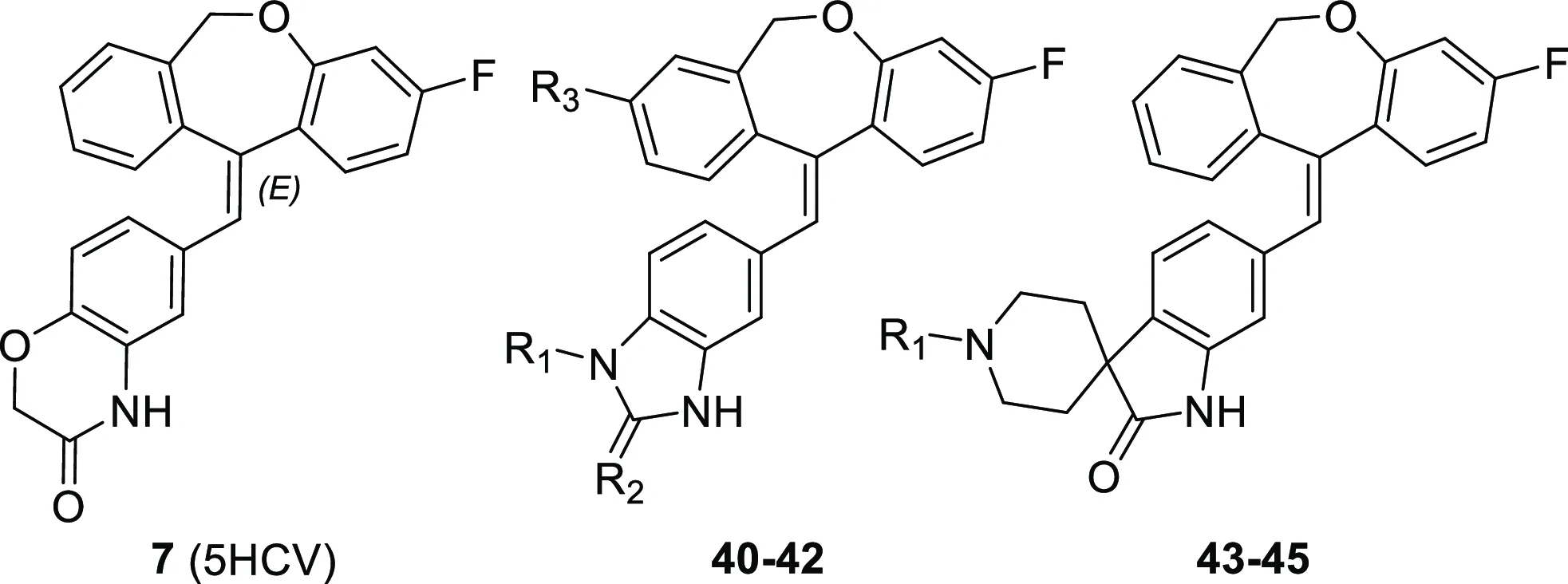

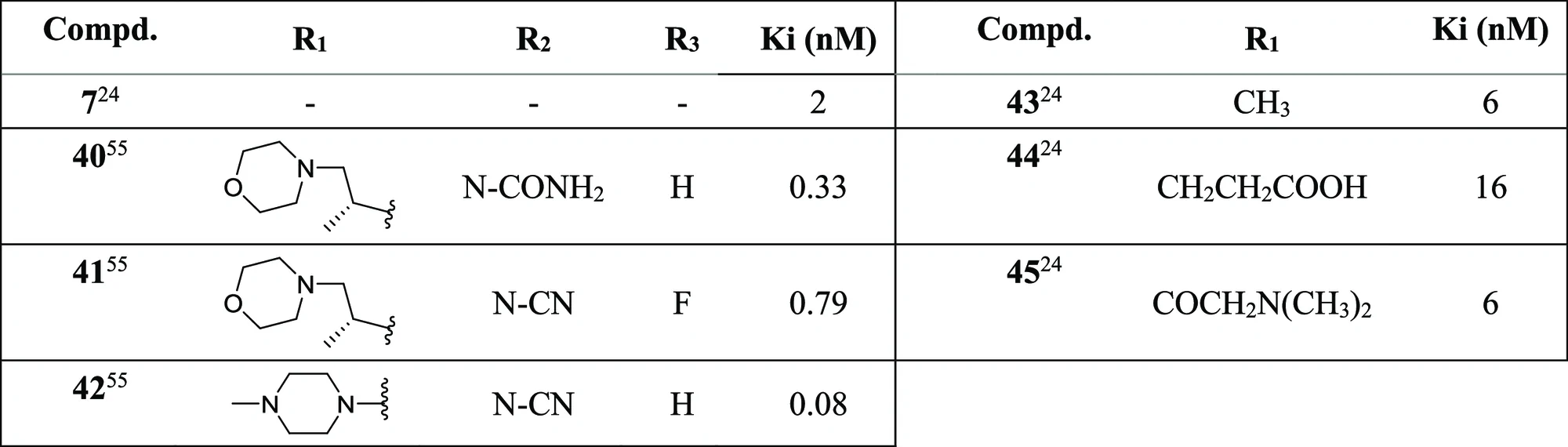
Binding Affinity for MR of a Selection of Dihydrodibenzo­[b,e]­oxepine-Containing Derivatives

**Figure 10:**
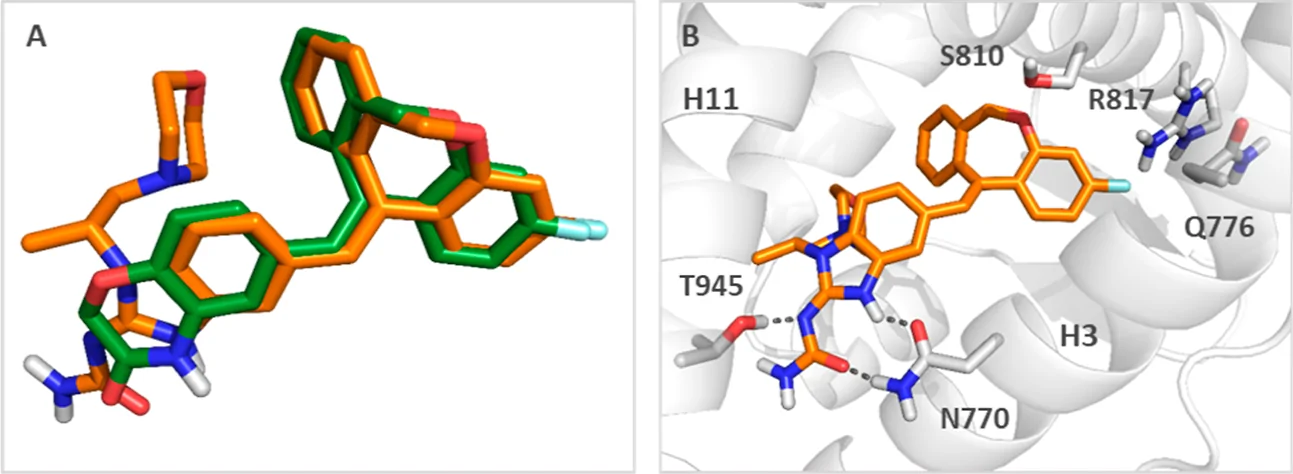
IFD studies on dihydrodibenzooxepine derivatives. (A) Superimposition of a representative IFD pose of derivative **40** (orange) with benzoxazinone **7** (green, PDB code 5HCV). (B) Representative IFP pose of the complex between derivative **40** (orange) and MR. Hydrogen bonds are depicted as gray dotted lines. For clarity, only polar hydrogens are shown.

### 3.4 Aryl Sulfonamide MR Ligands

A series of MR ligands containing a sulfonamide group has been identified (Table 4). Among them, apararenone (**46**) is worth mentioning, which progressed to phase II clinical trials before its development was discontinued in 2021.^57–60^ Although these derivatives include a benzoxazinone ring, the presence of a phenyl substituent on the cis-amide nitrogen likely prevents them from binding to MR in the same manner as previously discussed for benzoxazinones (**24–35**).

**Table 4:**
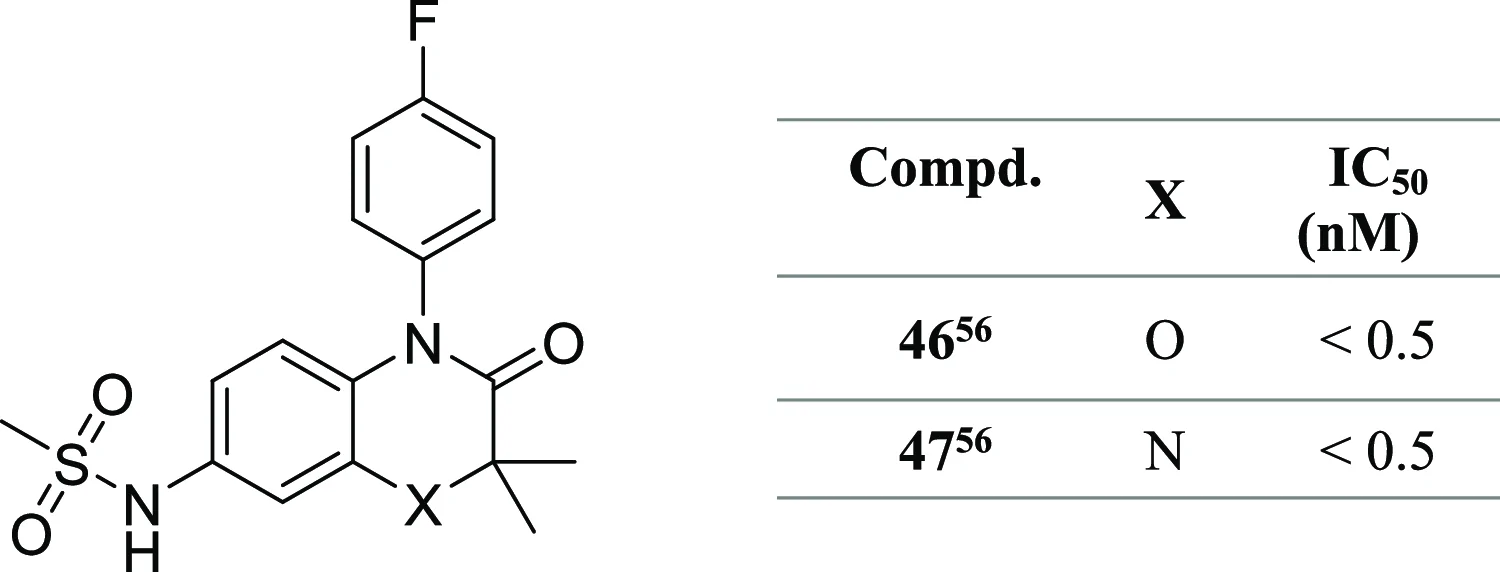
MR Binding Affinity of Sulfonamide Derivatives

Our IFD studies with derivatives **46** and **47** revealed that the sulfonamide group forms a hydrogen bond with MR residues Asn770 and Thr945 (Figure 11A). In contrast, in the benzoxazinone derivatives, these interactions are established through the cis-amide group (Figure 5C,D). Interestingly, the sulfonamide and the cis-amide moieties of these compounds overlap within the MR binding pocket (Figure 11B). Furthermore, the benzoxazinone ring of **46** partially overlaps with the central ring of derivative **5**, directing its carbonyl group toward Gln776 and Arg817 and forming water-mediated hydrogen bonds with these residues (Figure 11A,B). Additionally, the 4-fluorophenyl group occupies the same cavity as the corresponding group in the benzoxazinone derivatives, such as **5** (Figure 11B). To gain further insights into the binding mode of this family, molecular dynamics simulations were performed for the MR—**46** complex. The results indicate that the proposed binding pose remains stable across three 1000 ns replicas, consistently maintaining the bidentate hydrogen bonds with Asn770 (Supporting Information Figure S11). In contrast, the hydrogen bond with Thr945 is disrupted, due to a change in its side-chain dihedral angle, which enables the formation of a hydrogen bond with the backbone carbonyl of Phe941 in more than 95% of the trajectory (Figure 11C,D), similar to what was observed for compound **35**. The reproducibility and robustness of MD simulations of **46** are supported by the consistency of protein and ligand RMDS values, as well as total energy across the independent replicas (Supporting Information Figures S12 and S13)

**Figure 11:**
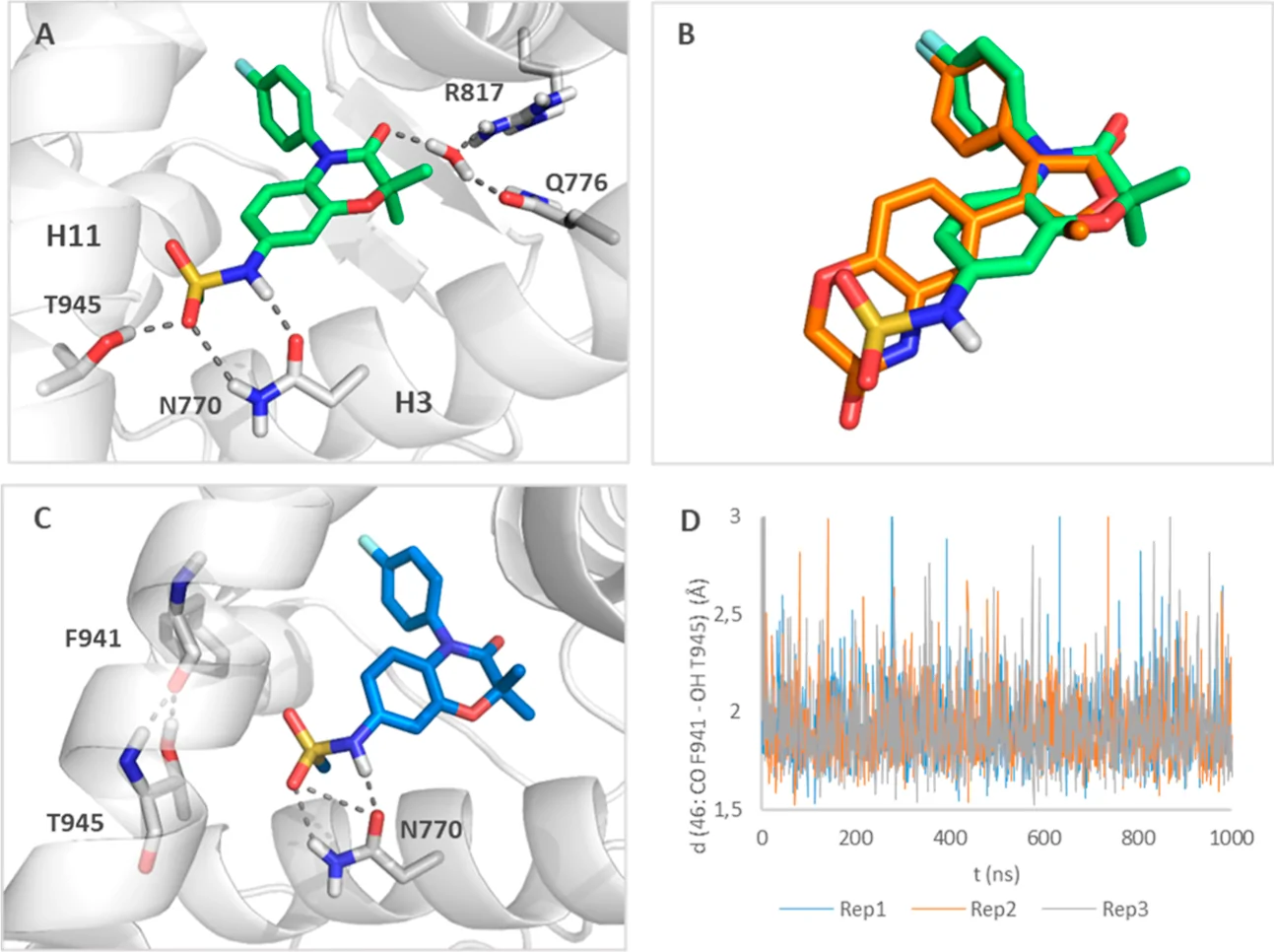
IFD and MD studies on sulfonamide-containing derivative **46**. (A) Representative IFP pose of the complex between derivative **46** (green) and MR. (B) Superimposition of a representative IFD pose of the complexes between MR and derivative **46** (green) and the one with benzoxazinone **5** (orange, PDB code 3WFG). (C) Representative 1000 ns frame of the MR—compound **46** complex from the MD simulations. (D) Time evolution of the distances between the hydroxyl hydrogen of Thr945 and the backbone carbonyl oxygen of Phe941 along the MD simulations of the MR—**46** complex. Hydrogen bonds are depicted as gray dotted lines. For clarity, only polar hydrogens are shown.

There are no available X-ray structures of MR complexes with ligands similar to **46** or **47**. However, there are some structures of compounds containing a sulfonamide group, such as **8 and 9** (PDB codes 5L7E and 5L7G, respectively,^25^ Figure 1). A comparison between these crystal structures and our IFD models for **46** or **47** revealed a good superimposition of the sulfonamide moiety (Figure 12A), which participates in polar interactions with Asn770 and Thr945. In addition, the 4-fluorophenyl ring of **46** adopts a similar orientation to that of the 2,3-fluorophenyl of **9** (PDB code 5L7G).

**Figure 12:**
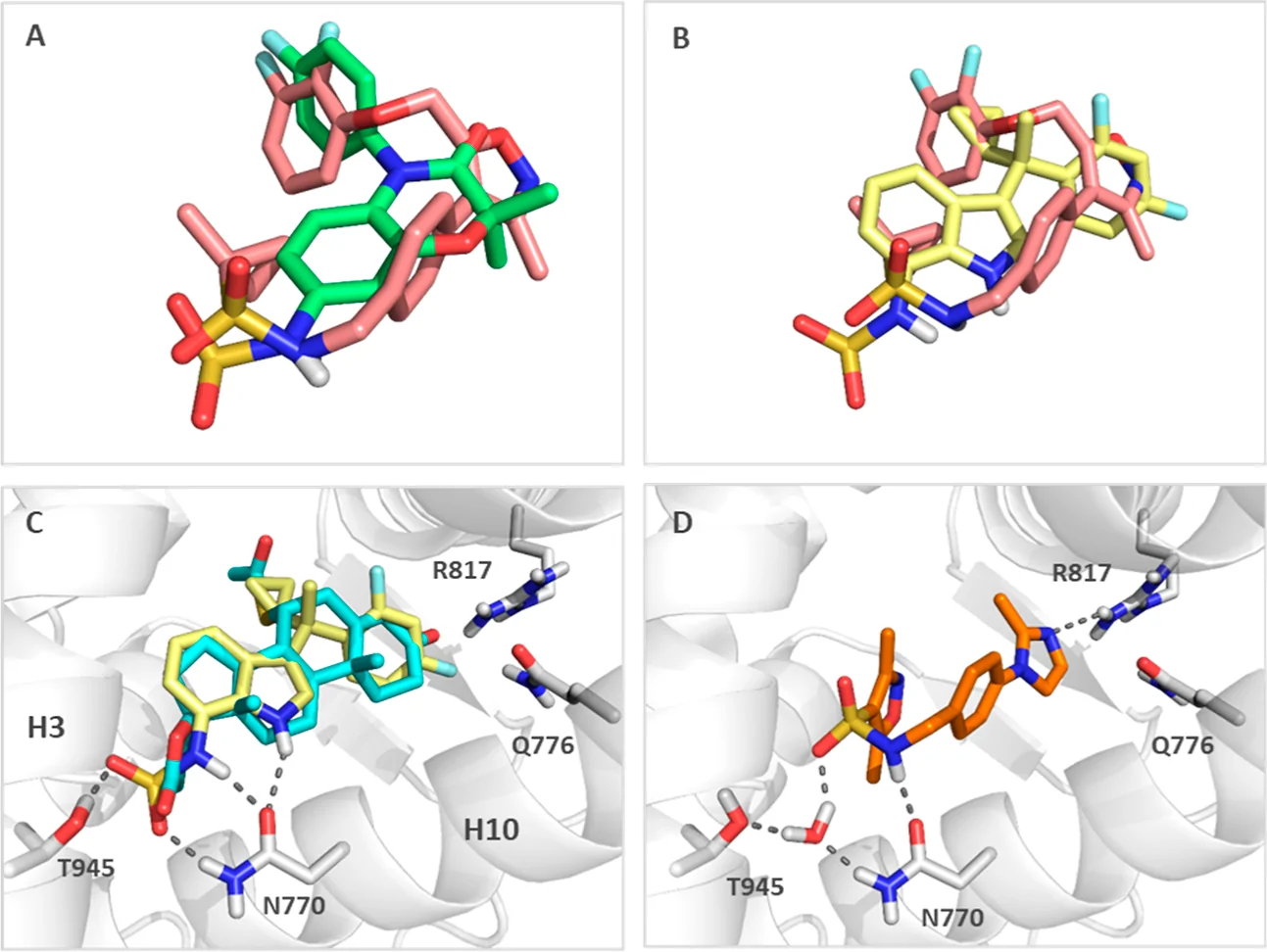
IFD studies on sulfonamide-containing derivatives. (A) Superimposition of the IFD pose of **46** (green) and **9** (pink, PDB code 5L7G). (B) Superimposition of the IFD pose of **49** (yellow) and **9** (pink, PDB code 5L7G). (C) Superimposition of the IFD pose of **49** (yellow) and spironolactone (cyan, PDB code 3VHU). (D) Representative IFP pose of the complex between derivative **50** (orange) and MR. Hydrogen bonds are depicted as gray dotted lines. For clarity, only polar hydrogens are shown.

The consistent disposition of the sulfonamide moiety across these compounds prompted us to explore whether other molecules containing sulfone or sulfonamide functional groups might exhibit a similar MR binding mode. To this end, we compared the IFD poses of a set of such derivatives (Table 5), including esaxerenone (**48**), a drug approved for clinical use in Japan in 2018.^58–62^ For these studies, we incorporated the MR structure bound to ligand **9** (PDB code 5L7G) in addition to the previously used models.

**Table 5:**
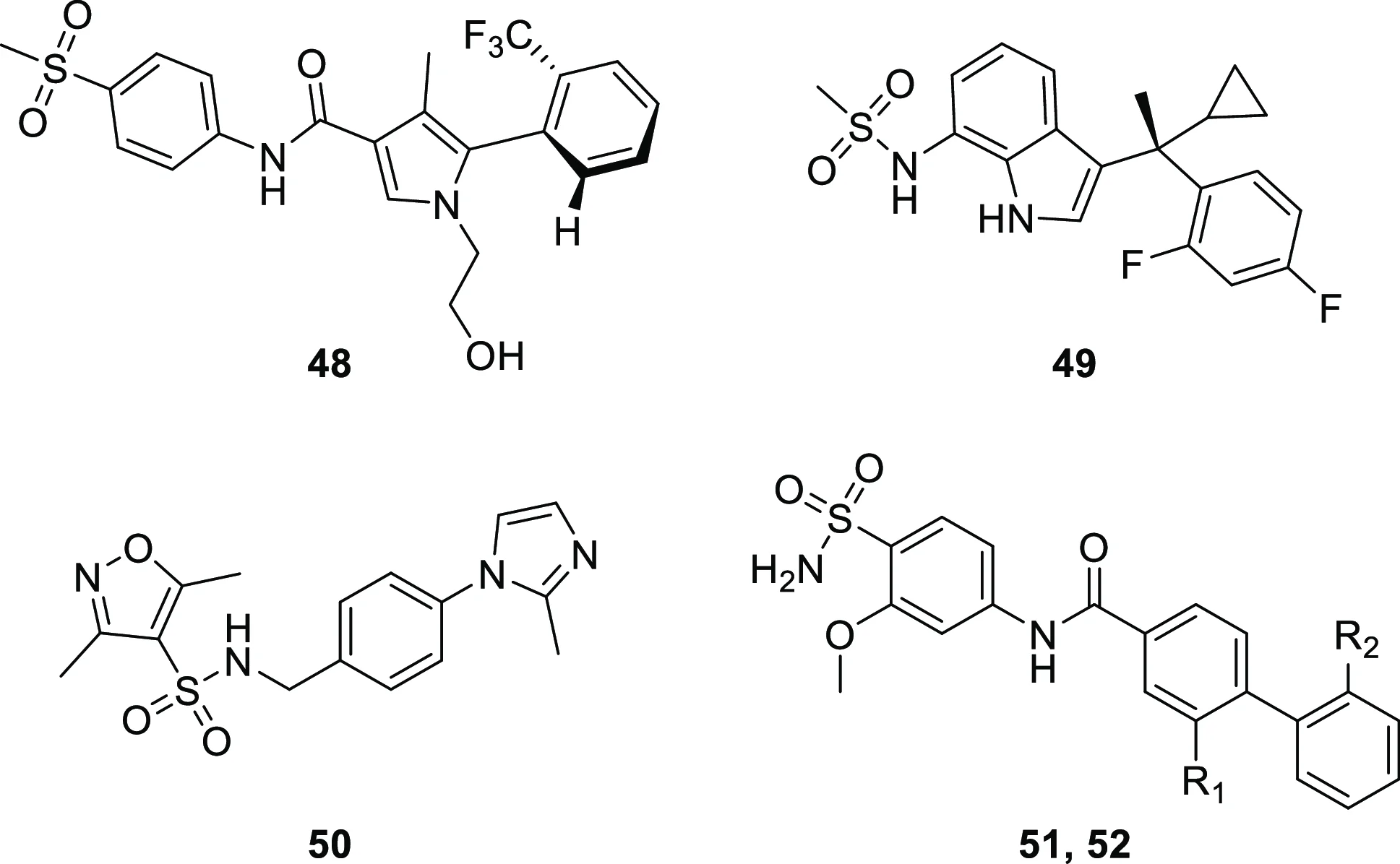

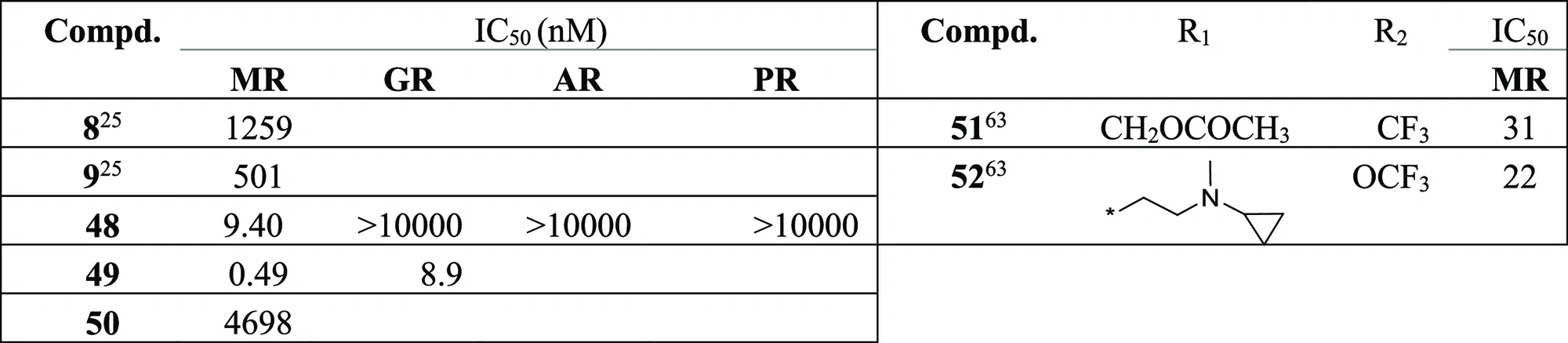
Chemical Structure and MR Binding Affinity of Selected Sulfones and Sulfonamide Derivatives

IFD studies revealed that only compounds **49** and **50** adopted binding poses in which the sulfonamide group superimposed with those of the cocrystallized ligands **8** and **9** (Figure 12B) and likewise participated in hydrogen bonds with Asn770 and Thr945 (Figure 12C). It is worth noting that in the case of derivative **50**, the hydrogen bond with Thr945 is mediated by a water molecule (Figure 12D), whereas in **49,** the indole amino group also participates in an additional hydrogen bond with Asn770 (Figure 12C), consistent with the binding mode previously proposed for **49** by Lotesta et al.^24^ Furthermore, these compounds possess functional groups that enable interactions with residues Gln776 and Arg817, in particular the imidazole ring of derivative **50** or the 4-phenyl fluorine atom in **49**. The orientation of the phenyl ring in **49** resembles that of 4-fluorophenyl observed in certain 1,4-DHPs (**10**-**15**). Interestingly, the cyclopropyl ring of **49** occupies the same selectivity-associated pocket as the C7 side chain of eplerenone. The close structural overlap of derivative **49** with spironolactone (Figure 12C), along with the hydrogen-bond network and the strong complementarity within the binding pocket, may explain its high binding affinity.

The 3D structure of esaxerenone, **48** (Table 5), bound to MR, resolved in 2020 (PDB code 6L88), was the first to demonstrate a displacement of helix H12.^42^ Notably, **48** exhibits a distinct binding mode compared to previously reported ligands. In our IFD studies, we deliberately opted not to use the 6L88 structure as a docking template, aiming instead to assess whether prior MR structures would predict a binding pose consistent with that observed in the experimental study. Interestingly, the top-ranked IFD pose of **48** using as template the 2A3I structure shows significant overlap, albeit with a certain displacement, with the experimentally determined structure 6L88, with a particular strong alignment of the sulfonamide groups (Figure 13A). While the orientation and conformation of esaxerenone were similar in both the X-ray and IFD studies, the IFD studies do not account for peptide backbone flexibility and were therefore unable to reproduce the structural rearrangements observed in several MR secondary elements, particularly the shift in helix H12. On the other hand, derivatives **51** and **52**, which share structural features with esaxerenone (**48**), exhibited similar binding modes in the IFD studies (Figure 13B). A comparison of the sulfonamide disposition in these compounds with that of previously analyzed derivatives **8**, **9, 48**, and **50** (Figure 1, Table 5) revealed different orientations. While the sulfonamide group of **48** analogues is oriented toward Asn770, in the other compounds, it is directed toward Arg817.

**Figure 13:**
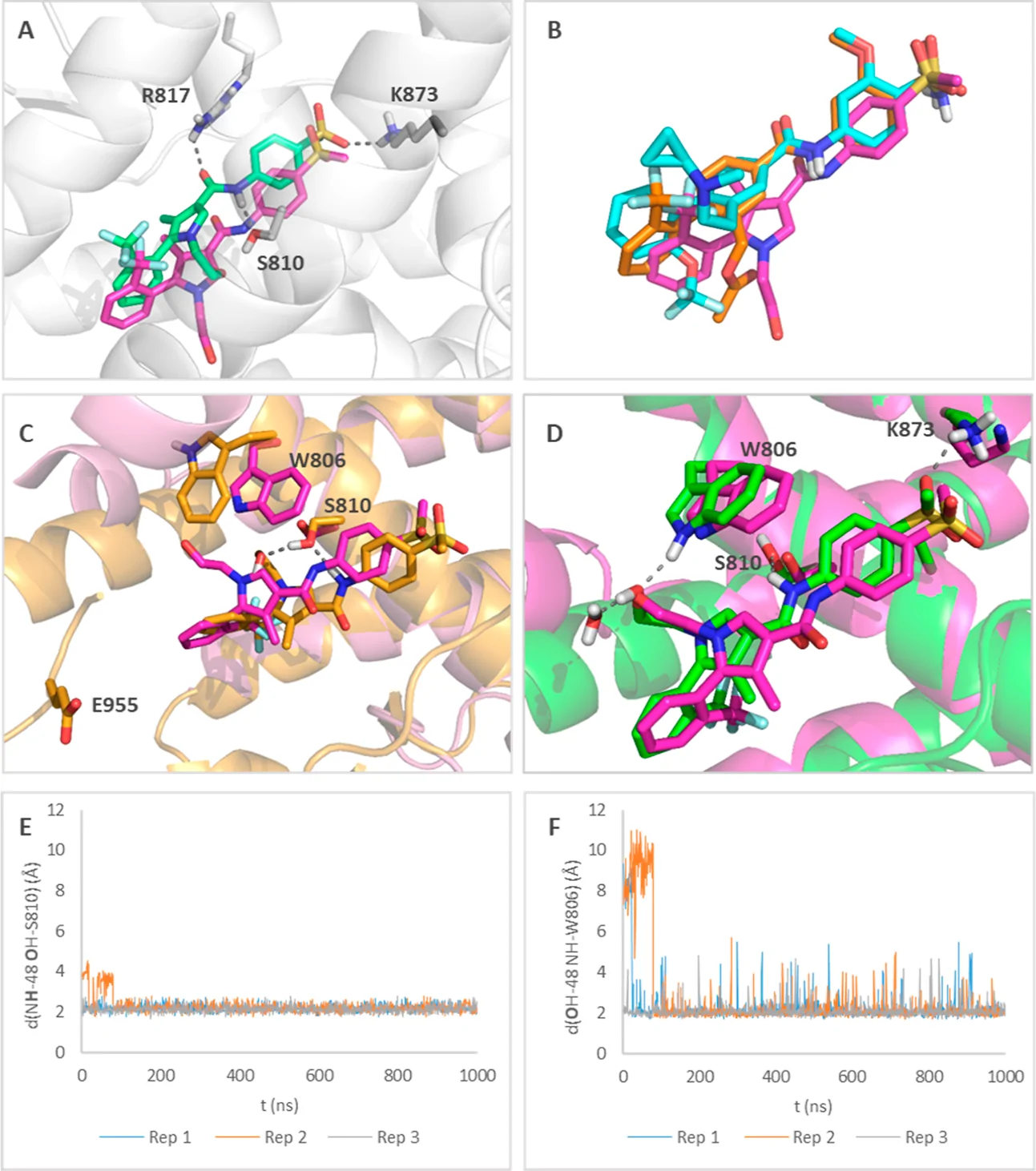
IFD and MD studies on esaxerenone (48). Superimposition of the X-ray structure of MR in complex with **48** (purple, PDB code 6L88) and representative: (A) IFD pose of derivative **48** (green). (B) IFD pose of derivatives **51** (orange) and **52** (blue). (C) IFD pose of **48** (orange) showing the disposition of Trp806. (D) Superimposition of a 1000 ns frame of **48** (green). (E) Distance between the amino proton of **48** and the hydroxyl oxygen of Ser810. (F) Distance between the hydroxyl oxygen of **48** and the amino proton of Trp806 side chain. Hydrogen bonds are depicted as gray dotted lines. For clarity, only polar hydrogens are shown.

Overall, the overlap between the esaxerenone (**48**) IFD pose and its X-ray structure (Figure 13A) validates our IFD studies, even for challenging ligands like **48** that exhibit a distinct binding mode. These findings are promising for future MR ligand discovery efforts using docking studies, as they demonstrate the ability to predict the binding modes of ligands that differ significantly from those previously characterized.

At this stage, it became of interest to determine whether a MD simulation, starting from a representative MR–esaxerenone (**48**) complex identified in our IFD studies, could result in the ligand shifting closer to its experimentally observed disposition. While MD studies are able to simulate helix movement, they require extensive simulation times. Therefore, helix H12, which undergoes a large displacement in the crystal structure, was removed before the MD studies. Monitoring the MR–**48** complex over three independent 1000 ns MD replicas showed a gradual convergence of the ligand’s disposition and orientation toward that observed in the X-ray structure (Figure 13D). MD replica consistency was confirmed by comparable structural and energetic descriptors (Supporting Information Figures S12 and S13). A hydrogen bond, between the amide amino proton of **48** and the side chain of Ser810, was observed for more than 88% of the simulation time (Figure 13D,E). Notably, these studies revealed a change in the Trp806 dihedral angle, which enabled the formation of an additional hydrogen bond between the hydroxyl group of **48** and the Trp806 side chain amino proton (Figure 13D,F), similar to what was observed in the MD simulations of finerenone (**23**) without helix H12 (see above). This flip in the Trp806 side chain was reported as necessary for esaxerenone binding to MR (PDB code 6L88). Besides, it brings this residue close to Ser810, a residue replaced by a Met in GR, AR, and PR, and it is considered a contributor to **48** MR selectivity.^42^ Although no hydrogen bonds with Trp806 were explicitly identified in this study, the Trp806 side chain is in close proximity to the hydroxyl oxygen of the pyrrole substituent of **48**.

Taken together, IFD studies showed that the sulfonyl group consistently occupies a similar position within the same MR binding pocket across structurally diverse compound families, including derivatives **8**, **46**, **49,** and **50** (Figure 12A,B). In this disposition, the sulfonyl group overlaps with the cis-amide of the benzoxazinone ring, present in compounds such as **6**, **26,** or **35** (Figure 11B). In contrast, other derivatives like **48**, **51,** or **52** position their sulfonyl groups in a distinct binding pocket, indicating a different binding mode for this family compared to the previous ones. It is worth noting that IFD studies were able to predict poses similar to those observed in the X-ray structures.

### 3.5 MR Ligands with a Central Pyrazoline Ring

Five-membered N-heterocyclic rings have emerged as key scaffolds in several MR ligands, many of which have already been discussed in the context of the benzoxazinone family. Additionally, a series of compounds featuring a central pyrazoline scaffold have been identified. This scaffold is substituted with three rings with at least two aromatic ones. Representative compounds include derivatives **53–55** (Table 6). More structurally restricted analogues were also explored, in which the pyrazoline ring is fused with one of the aromatic rings, resulting in a tricyclic structure as in derivatives **56** and **57** (Table 6).^64–66^ Among these restricted analogues, compound **56** (PF-3882845)^67^ entered phase I clinical studies but was ultimately discontinued.

**Table 6:**
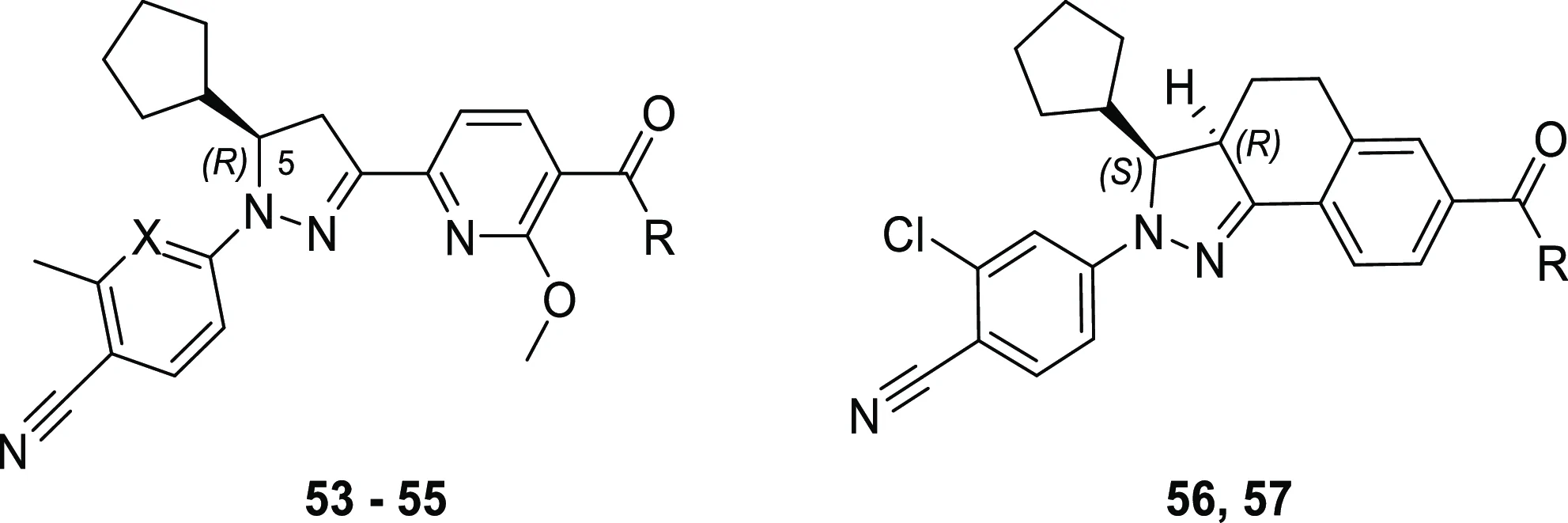
Binding Affinity of Selected Pyrazoline Derivatives

compd	substituent	IC_50_ (nM)				
	R	X	MR	PR	GR	AR
(*5R*) 53 ^66^	OH	CH	4.5	2530	>10000	>10000
(*5S*) 53 ^66^			4450			
54 ^66^	NH_2_	CH	2.7	494	2770	3250
55^66^	OH	N	7.6	1590	6800	9500
56 ^64^	OH	-	9	416	>10000	8960
57 ^65^	NH(CH_2_)_2_OH	-	3			

Our molecular docking studies of derivatives **53–57**, all with a central pyrazoline scaffold, revealed two predominant orientations within the MR binding site. In one orientation, the CN group is directed toward Arg817, while in the other, it is oriented toward Asn770. The former orientation was more frequently observed among the top-scoring poses and was the exclusive binding mode for the unconstrained derivative **54**. Visual inspection and interaction analysis further confirmed enhanced complementarity when the CN group was oriented toward Arg817. Therefore, we focused on this binding mode, which aligns well with the one proposed by Casimiro-Garcia.^66^

The analysis of the poses showed no significant differences between the 1,3,5-pyrazolines (**53**-**55**) and the tricyclic restricted analogues (**56**, **57**) (Figure 14A). When compared to steroid ligands such as spironolactone, the pyrazoline core and its aromatic substituents showed notable overlap with the steroid rings (Figure 14B). It is worth noting that, within the binding mode discussed above, derivatives **53–55** can adopt two alternative poses that are nearly superimposable. These poses differ mainly by a flip of the benzoic acid ring, which results in the orientation of the methoxy (OMe) group toward either helix 3 or helix 10 (containing Asn770 or Thr945, respectively) (Figure 14C). As expected, both disposition led to a similar hydrogen-bonding pattern. Specifically, the CN group of these derivatives form hydrogen bonds with Gln776, and in poses where OMe is orienting toward helix 10, an additional interaction with Arg817 is observed. Moreover, either the amide (**54**, **57**) or the carboxylic acid (**53**, **55**, **56**) participates in hydrogen bonds with Asn770 and/or Thr945 (Figure 14C,D). Interestingly, the cyclopentane moiety at position 5 of the pyrazoline ring adopts a similar orientation to the side chain at steroid position 7 of spironolactone (PDB code 3VHU), or the phenyl ring of ligand **5** (PDB code 3WFG), fitting into a hydrophobic pocket formed by Leu769, Leu814, Phe829, Met845, Met852, and Leu938. This pocket has been associated with MR selectivity,^21^ consistent with the selectivity observed for these derivatives. Interestingly, only 1,3,5-pyrazolines **53**–**55** with the 5*R* stereochemistry were able to optimally position the cyclopentyl ring within the hydrophobic pocket. The less favorable cyclopentyl ring fit, combined with the reduced complementarity of the (5*S*)-**53** derivative in the MR orthosteric site, may explain its 3-fold lower affinity compared to its 5*R* enantiomer (Table 6).

**Figure 14:**
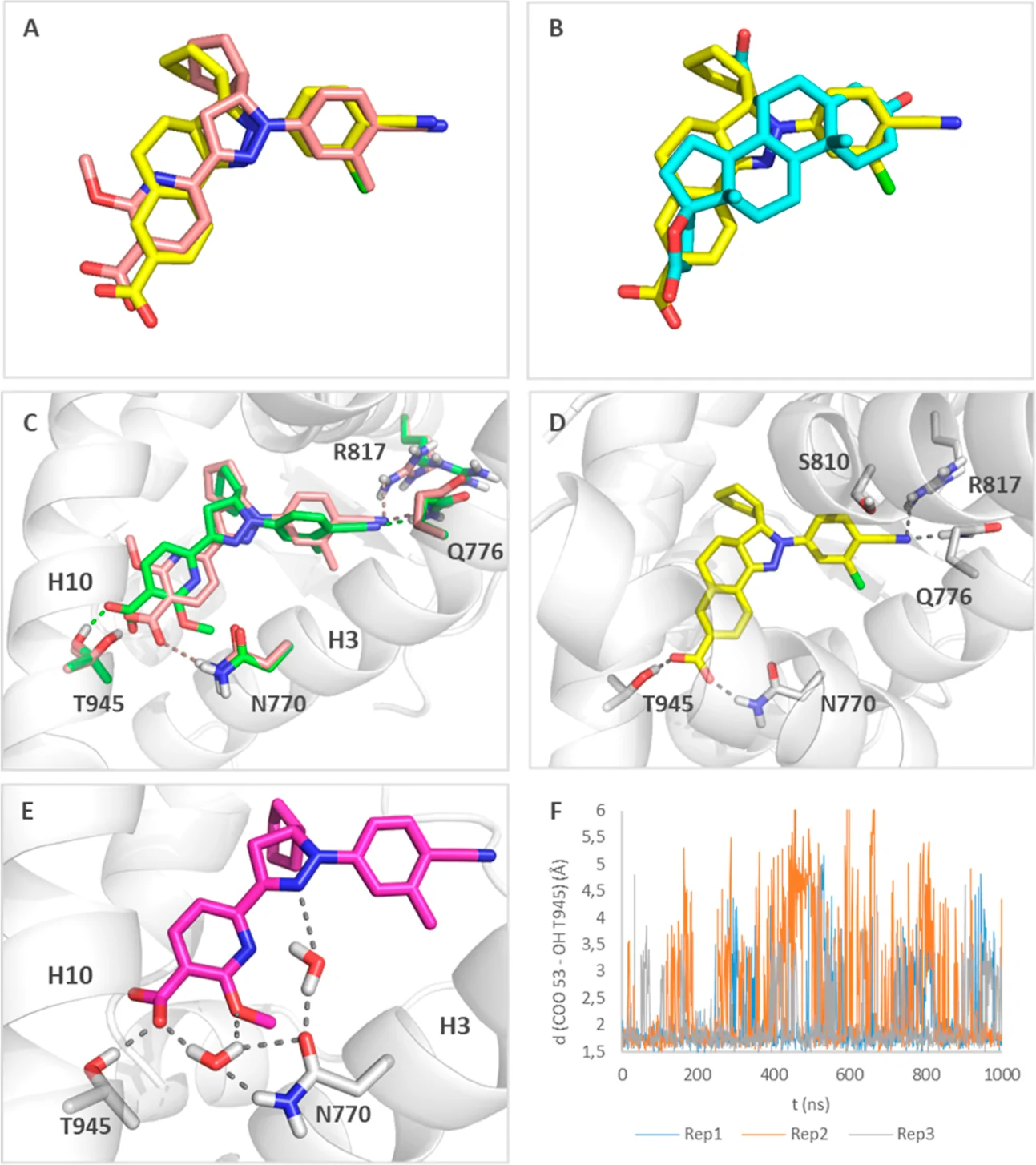
IFD studies on pyrazoline derivatives. (A) Superimposition of representative IFD poses of MR in complex with derivatives **53** (pink) and **56** (yellow). (B) Superimposition of representative IFD poses of MR in complex with **56** (yellow) and the MR-spironolactone X-ray structure (cyan, PDB code 3VHU). (C) Representative alternative IFD poses of **53** (green and pink). (D) Representative IFD pose of derivative **56** (yellow) bound to MR. (E) Representative 1000 ns frame of the MR—compound **53** (purple) complex from the MD simulations. (F) Time evolution of the distances between compound **53** carboxylic oxygen and the hydroxyl hydrogen of Thr945. Hydrogen bonds are depicted as gray dotted lines. For clarity, only polar hydrogens are shown.

To get further insights into the preferred binding conformation regarding OMe disposition, MD studies were carried out on the MR—compound **53** complexes. These simulations revealed that when OMe is initially oriented toward helix 10, two of the replicas exhibit a reduction in hydrogen bonds along the trajectory, whereas the third replica, which maintains an interaction with Thr945 throughout the 1000 ns simulation, undergoes a flip of the OMe chain, resulting in its reorientation toward helix 3. Consistently, MD simulations initiated from the IFD pose in which the OMe group is oriented toward helix 3 show greater stability (Supporting Information Figure S14). The only hydrogen-bond interaction maintained along the trajectory is that between the **53** carboxylic oxygen and Thr945 side chain, present in approximately 67–84% of the simulation time (Figure 14E,F). In addition, a water-mediated hydrogen bond involving Asn770 is observed in all three 1000 ns frames. Comparable structural and energetic profiles were observed across the three replicas (Supporting Information Figures S12 and S13)

Overall, the IFD studies provide valuable insights into the binding mode of the pyrazoline family with MR, highlighting the role of stereochemistry at position 5 in determining both binding affinity and receptor selectivity.

## 4 Conclusions

This study presents a comprehensive computational approach that clarifies the structural determinants for MR ligand affinity and selectivity and also reveals strategies to modulate coregulator recruitment—a mechanism worth exploring for MR ligands to overcome limitations of current MR antagonists, particularly in patients with renal comorbidities.

We implemented an integrated computational strategy combining IFD with long-time-scale MD simulations. Using the 1,4-DHP series as a benchmark, we demonstrate that IFD clearly outperforms a softened rigid docking approach in generating plausible binding poses. Moreover, the ability of IFD studies, complemented with MD refinement, to reproduce experimentally determined binding modes, including challenging cases such as esaxerenone, validates the computational framework used as a robust tool for characterizing MR ligand interactions and guiding future MR ligand discovery.

Our findings showed that high affinity can be achieved with a minimal scaffold composed of two aromatic rings connected by a three-bond linker, provided that there are polar groups capable of interacting with key residues in two distinct binding pockets, namely Asn770/Thr945 and Gln776/Arg817. MD studies consistently identify the interaction with Asn770 as the most persistent across trajectories, supporting its role as the primary anchoring residue for MR ligands and highlighting the importance of incorporating functional groups capable of engaging this site in MR ligand development. Notably, functional groups such as the cis-amide of benzoxazinone, along with urea and sulfonamide moieties, effectively clamp the Asn770 residue by forming two hydrogen bonds.

A critical challenge in clinical translation is achieving selectivity for MR. Our simulations reveal that MR selectivity is reinforced when ligands exploit at least two of three structural features unique to MR: hydrogen bonding with Ser810, proximity to Ala773, and occupation of an extended pocket that accommodates eplerenone’s C7 side chain. In this regard, the substituents identified to exploit this pocket include both aromatic and nonaromatic rings, highlighting this region as a versatile and attractive target for future MR ligand design.

Beyond binding, we propose a mechanistic link between coregulators’ recruitment and disruption of helix H12 packing mediated by ligand hydrogen bonding with Trp806. This interaction is consistently observed in the MD simulations of the two clinically approved nonsteroidal antagonist finerenone (**23**) and esaxerenone (**48**). These findings are significant, as they align with the hypothesis that disrupting MR coregulators recruitment could serve as a strategy to fine-tune downstream signaling, akin to the mechanism of selective estrogen receptor modulators (SERMs). In ER pharmacology, SERMs have demonstrated distinct patterns of coregulatory recruitment,^44,68^ providing a model that could be applied to MR modulation.

In summary, these studies provide reliable computational insights that enhance our understanding of the structural basis of MR ligand binding and selectivity over other steroid receptors. More importantly, they provide valuable perspectives on potential mechanisms for modulating coregulator recruitment, opening new avenues for the rational design of novel MR ligands with improved therapeutic profiles.

## Supplementary Material















## Data Availability

Input files, parameter sets, and output data required to reproduce the reported results are provided in Methods and in the Supporting Information. Schrödinger Suite release 2015 was used for molecular docking. This software is commercially available from Schrödinger, LLC (https://www.schrodinger.com/). Amber20 was used for dynamic simulations. This software is available at https://ambermd.org/.

## Notes

Dihedral distances (ZIP)
MR energy replicates (XLSX)
MR ligand IFD poses (ZIP)
MR ligand MD 1000 ns frames (ZIP)
MR ligand MD input files (ZIP)
MR RMSD replicates (XLSX)
Tables S1-S2 and Figures S1-S14 (PDF)

## Abbreviations

MR: mineralocorticoid receptor
AR: androgen receptor
PR: progesterone receptor
GR: glucocorticoid receptor
ER: estrogen receptor
MRAs: MR antagonists
LBD: ligand-binding domain
IFD: induced fit docking
MD: molecular dynamics
DHP: dihydropyridine
PDB: Protein Data Bank
Comd.: compound
IC_50_: half-maximal inhibitory concentration
SERM: selective estrogen receptor modulators.

## References

[1] Alexander S. P. H., Cidlowski J. A., Kelly E., Mathie A. A., Peters J. A., Veale E. L., Armstrong J. F., Faccenda E., Harding S. D., Davies J. A., Coons L., Fuller P. J., Korach K. S., Young M. J. (2023). The Concise Guide to PHARMACOLOGY 2023/24: Nuclear
Hormone Receptors. Br. J. Pharmacol..

[2] Grossmann C., Almeida-Prieto B., Nolze A., Alvarez de la Rosa D. (2022). Structural
and Molecular Determinants of Mineralocorticoid Receptor Signalling. Br. J. Pharmacol..

[3] Chapman K., Holmes M., Seckl J. (2013). 11beta-Hydroxysteroid Dehydrogenases:
Intracellular Gate-Keepers of Tissue Glucocorticoid Action. Physiol. Rev..

[4] Gomez-Sanchez E., Gomez-Sanchez C. E. (2014). The Multifaceted Mineralocorticoid
Receptor. Compr. Physiol..

[5] Jaisser F., Farman N. (2016). Emerging Roles of the Mineralocorticoid
Receptor in
Pathology: Toward New Paradigms in Clinical Pharmacology. Pharmacol. Rev..

[6] Gomez-Sanchez E. P. (2016). Third-Generation
Mineralocorticoid Receptor Antagonists: Why Do We Need a Fourth?. J. Cardiovasc. Pharmacol..

[7] Barrera-Chimal J., Bonnard B., Jaisser F. (2022). Roles of Mineralocorticoid
Receptors
in Cardiovascular and Cardiorenal Diseases. Annu. Rev. Physiol..

[8] Bauersachs J., López-Andrés N. (2022). Mineralocorticoid
Receptor in Cardiovascular
Diseases-Clinical Trials and Mechanistic Insights. Br. J. Pharmacol..

[9] Lother A., Jaisser F., Wenzel U. O. (2022). Emerging Fields for Therapeutic Targeting
of the Aldosterone-Mineralocorticoid Receptor Signaling Pathway. Br. J. Pharmacol..

[10] Paul S. N., Wingenfeld K., Otte C., Meijer O. C. (2022). Brain Mineralocorticoid
Receptor in Health and Disease: From Molecular Signalling to Cognitive
and Emotional Function. Br. J. Pharmacol..

[11] Ito S., Itoh H., Rakugi H., Okuda Y., Yoshimura M., Yamakawa S. (2020). Double-Blind Randomized
Phase 3 Study Comparing Esaxerenone
(CS-3150) and Eplerenone in Patients With Essential Hypertension (ESAX-HTN
Study). Hypertension.

[12] Pérez-Gordillo, F. L. ; Pérez de Vega, M. J. ; Gerona-Navarro, G. ; Rodríguez, Y. ; Alvarez de la Rosa, D. ; González-Muñiz, R. ; Martín-Martínez, M. Advances in the Development of Non-Steroidal Mineralocorticoid-Receptor Antagonists. In Aldosterone-Mineralocorticoid Receptor - Cell Biology to Translational Medicine; IntechOpen: Rijeka, 2019; p Ch. 16. 10.5772/intechopen.88417.

[13] Martín-Martínez M., Pérez-Gordillo F. L., Álvarez de La Rosa D., Rodríguez Y., Gerona-Navarro G., González-Muñiz R., Zhou M. M. (2017). Modulating Mineralocorticoid Receptor with Non-Steroidal
Antagonists. New Opportunities for the Development of Potent and Selective
Ligands without Off-Target Side Effects. J.
Med. Chem..

[14] Bledsoe R. K., Madauss K. P., Holt J. A., Apolito C. J., Lambert M. H., Pearce K. H., Stanley T. B., Stewart E. L., Trump R. P., Willson T. M. H., Williams S. P. (2005). A Ligand-Mediated Hydrogen Bond Network
Required for the Activation of the Mineralocorticoid Receptor. J. Biol. Chem..

[15] Fagart J., Huyet J., Pinon G. M., Rochel M., Mayer C., Rafestin-Oblin M. E. (2005). Crystal Structure of a Mutant Mineralocorticoid Receptor
Responsible for Hypertension. Nat. Struct. Mol.
Biol..

[16] Li Y., Suino K., Daugherty J., Xu H. E. (2005). Structural and Biochemical
Mechanisms for the Specificity of Hormone Binding and Coactivator
Assembly by Mineralocorticoid Receptor. Mol.
Cell.

[17] Fettweis G., Johnson T. A., Almeida-Prieto B., Weller-Pérez J., Presman D. M., Hager G. L., Alvarez
de la Rosa D. (2024). The Mineralocorticoid
Receptor Forms Higher Order Oligomers upon DNA Binding. Protein Sci..

[18] Sastry G.
M., Adzhigirey M., Day T., Annabhimoju R., Sherman W. (2013). Protein and Ligand Preparation: Parameters,
Protocols,
and Influence on Virtual Screening Enrichments. J. Comput. Aided. Mol. Des..

[19] Friesner R. A., Banks J. L., Murphy R. B., Halgren T. A., Klicic J. J., Mainz D. T., Repasky M. P., Knoll E. H., Shelley M., Perry J. K., Shaw D. E., Francis P., Shenkin P. S. (2004). Glide:
A New Approach for Rapid, Accurate Docking and Scoring. 1. Method
and Assessment of Docking Accuracy. J. Med.
Chem..

[20] Halgren T.
A., Murphy R. B., Friesner R. A., Beard H. S., Frye L. L., Pollard W. T., Banks J. L. G. (2004). A New Approach for Rapid, Accurate
Docking and Scoring. 2. Enrichment Factors in Database Screening. J. Med. Chem..

[21] Hasui T., Matsunaga N., Ora T., Ohyabu N., Nishigaki N., Imura Y., Igata Y., Matsui H., Motoyaji T., Tanaka T., Habuka N., Sogabe S., Ono M., Siedem C. S., Tang T. P., Gauthier C., De Meese L. A., Boyd S. A., Fukumoto S. (2011). Identification of Benzoxazin-3-One
Derivatives as Novel, Potent, and Selective Nonsteroidal Mineralocorticoid
Receptor Antagonists. J. Med. Chem..

[22] Hasui T., Ohra T., Ohyabu N., Asano K., Matsui H., Mizukami A., Habuka N., Sogabe S., Endo S., Siedem C. S., Tang T. P., Gauthier C., De Meese L. A., Boyd S. A., Fukumoto S. (2013). Design, Synthesis,
and Structure–Activity
Relationships of Dihydrofuran-2-One and Dihydropyrrol-2-One Derivatives
as Novel Benzoxazin-3-One-Based Mineralocorticoid Receptor Antagonists. Bioorg. Med. Chem..

[23] Hasui T., Ohyabu N., Ohra T., Fuji K., Sugimoto T., Fujimoto J., Asano K., Oosawa M., Shiotani S., Nishigaki N., Kusumoto K., Matsui H., Mizukami A., Habuka N., Sogabe S., Endo S., Ono M., Siedem C. S., Tang T. P., Gauthier C., De Meese L. A., Boyd S. A., Fukumoto S. (2014). Discovery of 6-[5-(4-Fluorophenyl)-3-Methyl-Pyrazol-4-Yl]-Benzoxazin-3-One
Derivatives as Novel Selective Nonsteroidal Mineralocorticoid Receptor
Antagonists. Bioorg. Med. Chem..

[24] Lotesta S. D., Marcus A. P., Zheng Y., Leftheris K., Noto P. B., Meng S., Kandpal G., Chen G., Zhou J., McKeever B., Bukhtiyarov Y., Zhao Y., Lala D. S., Singh S. B., McGeehan G. M. (2016). Identification
of Spirooxindole and Dibenzoxazepine Motifs as Potent Mineralocorticoid
Receptor Antagonists. Bioorg. Med. Chem..

[25] Nordqvist A., O’Mahony G., Fridén-Saxin M., Fredenwall M., Hogner A., Granberg K. L., Aagaard A., Bäckström S., Gunnarsson A., Kaminski T., Xue Y., Dellsén A., Hansson E., Hansson P., Ivarsson I., Karlsson U., Bamberg K., Hermansson M., Georgsson J., Lindmark B., Edman K., Friden-Saxin M., Fredenwall M., Hogner A., Granberg K. L., Aagaard A., Baeckstroem S., Gunnarsson A., Kaminski T., Xue Y., Dellsen A., Hansson E., Hansson P., Ivarsson I., Karlsson U., Bamberg K., Hermansson M., Georgsson J., Lindmark B., Edman K., Fridén-Saxin M., Fredenwall M., Hogner A., Granberg K. L., Aagaard A., Bäckström S., Gunnarsson A., Kaminski T., Xue Y., Dellsén A., Hansson E., Hansson P., Ivarsson I., Karlsson U., Bamberg K., Hermansson M., Georgsson J., Lindmark B., Edman K. (2017). Structure-Based Drug Design of Mineralocorticoid
Receptor Antagonists to Explore Oxosteroid Receptor Selectivity. ChemMedChem.

[26] Sherman W., Beard H. S., Farid R. (2006). Use of an Induced Fit Receptor Structure
in Virtual Screening. Chem. Biol. Drug Des..

[27] Sherman W., Day T., Jacobson M. P., Friesner R. A., Farid R. (2006). Novel Procedure for
Modeling Ligand/Receptor Induced Fit Effects. J. Med. Chem..

[28] Jacobson M. P., Friesner R. A., Xiang Z., Honig B. (2002). On the Role of the
Crystal Environment in Determining Protein Side-Chain Conformations. J. Mol. Biol..

[29] Jacobson M. P., Pincus D. L., Rapp C. S., Day T. J. F., Honig B., Shaw D. E., Friesner R. A. (2004). A Hierarchical Approach
to All-Atom
Protein Loop Prediction. Proteins.

[30] Case, D. A. ; Aktulga, H. M. ; Belfon, K. ; Ben-Shalom, I. Y. ; Berryman, J. T. ; Brozell, S. R. ; Cerutti, D. S. ; Cheatham, T. E. ; Cisneros, G. A. ; Cruzeiro, V. W. D. ; Darden, T. A. ; Duke, R. E. ; Giambasu, G. ; Gilson, M. K. ; Gohlke, H. ; Goetz, A. W. ; Harris, R. ; Izadi, S. ; Izmailov, S. A. ; Kasavajhala, K. ; Kaymak, M. C. ; King, E. ; Kovalenko, A. ; Kurtzman, T. ; Lee, T. S. ; LeGrand, S. ; Li, P. ; Lin, C. ; Liu, J. ; Luchko, T. ; Luo, R. ; Machado, M. ; Man, V. ; Manathunga, M. ; Merz, K. M. ; Miao, Y. ; Mikhailovskii, O. ; Monard, G. ; Nguyen, H. ; Onufriev, A. ; Pan, F. ; Pantano, S. ; Qi, R. ; Rahnamoun, A. ; Roe, D. R. ; Roitberg, A. ; Sagui, C. ; Schott-Verdugo, S. ; Shajan, A. ; Shen, J. ; Simmerling, C. L. ; Skrynnikov, N. R. ; Smith, J. ; Swails, J. ; Walker, R. C. ; Wang, J. ; Wang, J. ; Wei, H. ; Wolf, R. M. ; Wu, X. ; Xiong, Y. ; Xue, Y. ; York, D. M. ; Zhao, S. ; Kollman, P. A. AMBER; University of California: San Francisco, 2020.

[31] Jorgensen W. L., Chandrasekhar J., Madura J. D., Impey R. W., Klein M. L. (1983). Comparison
of Simple Potential Functions for Simulating Liquid Water. J. Chem. Phys..

[32] Liang J. J., Cao S., Hung A., El-Osta A., Karagiannis T. C., Young M. J. (2025). In Silico Investigation of Mineralocorticoid Receptor
Antagonists: Insights into Binding Mechanisms and Structural Dynamics. Molecules.

[33] Fagart J., Hillisch A., Huyet J., Bärfacker L., Fay M., Pleiss U., Pook E., Schäfer S., Rafestin-Oblin M.-E., Kolkhof P. (2010). A New Mode of Mineralocorticoid
Receptor
Antagonism by a Potent and Selective Nonsteroidal Molecule. J. Biol. Chem..

[34] Arhancet G. B., Woodard S. S., Iyanar K., Case B. L., Woerndle R., Dietz J. D., Garland D. J., Collins J. T., Payne M. A., Blinn J. R., Pomposiello S. I., Hu X., Heron M. I., Huang H.-C., Lee L. F. (2010). Discovery of Novel Cyanodihydropyridines
as Potent Mineralocorticoid Receptor Antagonists. J. Med. Chem..

[35] Arhancet G. B., Woodard S. S., Dietz J. D., Garland D. J., Wagner G. M., Iyanar K., Collins J. T., Blinn J. R., Numann R. E., Hu X., Huang H. C. (2010). Stereochemical Requirements for the Mineralocorticoid
Receptor Antagonist Activity of Dihydropyridines. J. Med. Chem..

[36] Bärfacker L., Kuhl A., Hillisch A., Grosser R., Figueroa-Pérez S., Heckroth H., Nitsche A., Ergüden J.-K., Gielen-Haertwig H., Schlemmer K.-H., Mittendorf J., Paulsen H., Platzek J., Kolkhof P. (2012). Discovery of BAY 94–8862:
A Nonsteroidal Antagonist of the Mineralocorticoid Receptor for the
Treatment of Cardiorenal Diseases. ChemMedChem.

[37] Pérez-Gordillo F. L., Serrano-Morillas N., Acosta-García L. M., Aranda M. T., Passeri D., Pellicciari R., Pérez de Vega M. J., González-Muñiz R., Alvarez de la Rosa D., Martín-Martínez M. (2023). Novel 1,4-Dihydropyridine
Derivatives
as Mineralocorticoid Receptor Antagonists. Int.
J. Mol. Sci..

[38] Fagart J., Wurtz J. M., Souque A., Hellal-Levy C., Moras D., Rafestin-Oblin M. E. (1998). Antagonism
in the Human Mineralocorticoid
Receptor. EMBO J..

[39] Auzou G., Fagart J., Souque A., Hellal-Levy C., Wurtz J. M., Moras D., Rafestin-Oblin M. E. (2000). A Single
Amino Acid Mutation of Ala-773 in the Mineralocorticoid Receptor Confers
Agonist Properties to 11beta-Substituted Spirolactones. Mol. Pharmacol..

[40] Geller D. S., Farhi A., Pinkerton N., Fradley M., Moritz M., Spitzer A., Meinke G., Tsai F. T., Sigler P. B., Lifton R. P. (2000). Activating Mineralocorticoid Receptor Mutation in Hypertension
Exacerbated by Pregnancy. Science.

[41] Hultman M. L., Krasnoperova N. V., Li S., Du S., Xia C., Dietz J. D., Lala D. S., Welsch D. J., Hu X. (2005). The Ligand-Dependent
Interaction of Mineralocorticoid Receptor with Coactivator and Corepressor
Peptides Suggests Multiple Activation Mechanisms. Mol. Endocrinol..

[42] Takahashi M., Ubukata O., Homma T., Asoh Y., Honzumi M., Hayashi N., Saito K., Tsuruoka H., Aoki K., Hanzawa H. (2020). Crystal Structure of the Mineralocorticoid Receptor
Ligand-Binding Domain in Complex with a Potent and Selective Nonsteroidal
Blocker, Esaxerenone (CS-3150). FEBS Lett..

[43] Amazit L., Le Billan F., Kolkhof P., Lamribet K., Viengchareun S., Fay M. R., Khan J. A., Hillisch A., Lombès M., Rafestin-Oblin M.-E.
E., Fagart J., Amazit F., Kolkhof P., Lamriber K., Viengchareum S., Fay M. R., Khan J. A., Hillisch A., Lombès M., Rafestin-Oblin M.-E., Fagart J. L. L. B., Le Billan F., Kolkhof P., Lamribet K., Viengchareun S., Fay M. R., Khan J. A., Hillisch A., Lombès M., Rafestin-Oblin M.-E.
E., Fagart J. (2015). Finerenone
Impedes Aldosterone-Dependent Nuclear Import of the Mineralocorticoid
Receptor and Prevents Genomic Recruitment of Steroid Receptor Coactivator-1. J. Biol. Chem..

[44] Koca D., Lother A. (2023). Molecular Pharmacology of Mineralocorticoid Receptor
Antagonists: The Role of Co-Regulators. Steroids.

[45] Bamberg K., Johansson U., Edman K., William-Olsson L., Myhre S., Gunnarsson A., Geschwindner S., Aagaard A., Björnson Granqvist A., Jaisser F., Huang Y., Granberg K. L., Jansson-Löfmark R., Hartleib-Geschwindner J., Aagaard A., Jaisser F. F., Huang Y., William-Olsson L., Myhre S., Gunnarsson A., Geschwindner S., Aagaard A., Granqvist A. B., Jaisser F. F., Huang Y., Granberg K. L., Jansson-Löfmark R., Hartleib-Geschwindner J. (2018). Preclinical Pharmacology of AZD9977:
A Novel Mineralocorticoid Receptor Modulator Separating Organ Protection
from Effects on Electrolyte Excretion. PLoS
One.

[46] Granberg K. L., Yuan Z.-Q. Q., Lindmark B., Edman K., Kajanus J., Hogner A., Malmgren M., O’Mahony G., Nordqvist A., Lindberg J., Tångefjord S., Kossenjans M., Löfberg C., Brånalt J., Liu D., Selmi N., Nikitidis G., Nordberg P., Hayen A., Aagaard A., Hansson E., Hermansson M., Ivarsson I., Jansson-Löfmark R., Karlsson U., Johansson U., William-Olsson L., Hartleib-Geschwindner J., Bamberg K., O’Mahony G., Nordqvist A., Lindberg J., Tångefjord S., Kossenjans M., Löfberg C., Brånalt J., Liu D., Selmi N., Nikitidis G., Nordberg P., Hayen A., Aagaard A., Hansson E., Hermansson M., Ivarsson I., Jansson-Löfmark R., Karlsson U., Johansson U., William-Olsson L., Hartleib-Geschwindner J., Bamberg K., O’Mahony G., Nordqvist A., Lindberg J., Tångefjord S., Kossenjans M., Löfberg C., Brånalt J., Liu D., Selmi N., Nikitidis G., Nordberg P., Hayen A., Aagaard A., Hansson E., Hermansson M., Ivarsson I., Jansson-Löfmark R., Karlsson U., Johansson U., William-Olsson L., Hartleib-Geschwindner J., Bamberg K. (2019). Identification of Mineralocorticoid Receptor Modulators
with Low Impact on Electrolyte Homeostasis but Maintained Organ Protection. J. Med. Chem..

[47] A Phase III, Randomised, Double-Blind Study to Evaluate the Effect of Balcinrenone/Dapagliflozin, Compared With Dapagliflozin, on the Risk of Heart Failure Events and Cardiovascular Death in Patients With Heart Failure and Impaired Kidney Function. (accessed February 6, 2024). https://clinicaltrials.gov/study/NCT06307652.

[48] Casimiro-Garcia, A. ; Futatsugi, K. ; Piotrowski, D. W. Morpholine Compounds as Mineralocorticoid Receptor Antagonists. WO2011/141848A1, 2011. http://wo2011/141848A1.

[49] Michellys, P. Y. ; Pei, W. ; Petrassi, H. M. ; Richmond, W. Compounds and Compositions as Modulators of Steroid Hormone Nuclear Receptors. WO2006/015259, 2006. http://wo2006/015259.

[50] Nariai T., Fujita K., Mori M., Katayama S., Hori S., Matsui K. (2011). SM-368229, a Novel
Selective and Potent Non-Steroidal
Mineralocorticoid Receptor Antagonist with Strong Urinary Na+ Excretion
Activity. J. Pharmacol. Sci..

[51] Boyer, S. ; Guo, X. ; Wu, D. ; Wu, F. Pyridyl Ureas as Mineralocorticoid Receptor Antagonists. WO2012/064631A1, 2012. http://wo2012/064631A1.

[52] Coghlan, M. J. ; Green, J. E. ; Grese, T. A. ; Jadhav, P. K. ; Matthews, D. P. ; Steinberg, M. I. ; Fales, K. R. ; Bell, M. G. Tricyclic Steroid Hormone Nuclear Receptor Modulators. WO2004/052847A2, 2004. http://wo2004/052847A2.

[53] Coghlan, M. J. ; Jadhav, P. K. ; Droste, J. J. ; Green, J. E. ; Matthews, D. P. Tricyclic Steroid Hormone Nuclear Receptor Modulators. WO2005/066153A1, 2005. http://wo2005/066153A1.

[54] Gavardinas, K. ; Green, J. E. ; Jadhav, P. K. ; Matthews, D. P. Tryciclic Steroid Hormone Nuclear Receptor Modulators. WO2005/066161A1, 2005. http://wo2005/066161A1.

[55] Gavardinas, K. ; Jadhav, P. K. 6H-Dibenzo­[B,E]­Oxepine Derived Nonsteroidal Mineralocorticoid Receptor Antagonist. WO2009/085584A1, 2009.

[56] Coates, D. A. ; Gavardinas, K. ; Jadhav, P. K. Mineralocorticoid Receptor Antagonist and Methods of Use. WO2010/104721A1, 2010. http://wo2010/104721A1.

[57] Iijima, T. ; Yamamoto, Y. ; Akatsuka, H. ; Kawaguchi, T. Benzoxazines and Related Nitrogen-Containing Heterobicyclic Compounds Useful as Mineralocorticoid Receptor Modulating Agents. WO2007/089034, 2007. http://wo2007/089034.

[58] Okanoue T., Sakamoto M., Harada K., Inagaki M., Totsuka N., Hashimoto G., Kumada H. (2021). Efficacy and Safety of Apararenone
(MT-3995) in Patients with Nonalcoholic Steatohepatitis: A Randomized
Controlled Study. Hepatol. Res..

[59] Wada T., Inagaki M., Yoshinari T., Terata R., Totsuka N., Gotou M., Hashimoto G. (2021). Apararenone
in Patients with Diabetic
Nephropathy: Results of a Randomized, Double-Blind, Placebo-Controlled
Phase 2 Dose–Response Study and Open-Label Extension Study. Clin. Exp. Nephrol..

[60] Apararenone - Mitsubishi Tanabe Pharma - AdisInsight. https://adisinsight.springer.com/drugs/800032686 (accessed 06, 17, 2022).

[61] Arai K., Homma T., Morikawa Y., Ubukata N., Tsuruoka H., Aoki K., Ishikawa H., Mizuno M., Sada T. (2015). Pharmacological
Profile of CS-3150, a Novel, Highly Potent and Selective Non-Steroidal
Mineralocorticoid Receptor Antagonist. Eur.
J. Pharmacol..

[62] Arai K., Tsuruoka H., Homma T. (2015). CS-3150, a
Novel Non-Steroidal Mineralocorticoid
Receptor Antagonist, Prevents Hypertension and Cardiorenal Injury
in Dahl Salt-Sensitive Hypertensive Rats. Eur.
J. Pharmacol..

[63] Katayama, S. ; Hori, S. ; Hasegawa, F. ; Suzuki, K. Biaryl Amide Derivative or Pharmaceutically Acceptable Salt Thereof. U.S. Patent WO2013/116227A1, 2013. http://us2013116227a1.

[64] Meyers M. J., Arhancet G. B., Hockerman S. L., Chen X., Long S. A., Mahoney M. W., Rico J. R., Garland D. J., Blinn J. R., Collins J. T., Yang S., Huang H.-C., McGee K. F., Wendling J. M., Dietz J. D., Payne M. A., Homer B. L., Heron M. I., Reitz D. B., Hu X. (2010). Discovery of (3S,3aR)-2-(3-Chloro-4-Cyanophenyl)-3-Cyclopentyl-3,3a,4,5-Tetrahydro-2H-Benzo­[g]­Indazole-7-Carboxylic
Acid (PF-3882845), an Orally Efficacious Mineralocorticoid Receptor
(MR) Antagonist for Hypertension and Nephropathy. J. Med. Chem..

[65] Meyers, M. J. ; Arhancet, G. B. ; Chen, X. ; Hockerman, S. L. ; Long, S. A. ; Mahoney, M. W. ; Reitz, D. B. ; Rico, J. G. ; Long Scott, A. ; Mahoney, M. W. ; Reitz, D. B. ; Rico, J. G. Pyrazoline Compounds As Mineralocorticoid Receptor Antagonists. WO2008053300A1, May 8, 2008.

[66] Casimiro-Garcia A., Piotrowski D. W., Ambler C., Arhancet G. B., Banker M. E., Banks T., Boustany-Kari C. M., Cai C., Chen X., Eudy R., Hepworth D., Hulford C. A., Jennings S. M., Loria P. M., Meyers M. J., Petersen D. N., Raheja N. K., Sammons M., She L., Song K., Vrieze D., Wei L. (2014). Identification of (R)-6-(1-(4-Cyano-3-Methylphenyl)-5-Cyclopentyl-4,5-Dihydro-1H-Pyrazol-3-Yl)-2-Me
Thoxynicotinic Acid, a Highly Potent and Selective Nonsteroidal Mineralocorticoid
Receptor Antagonist. J. Med. Chem..

[67] Orena S., Maurer T., She L., Eudy R., Bernardo V., Dash D., Loria P., Banker M. E., Tugnait M., Okerberg C., Qian J., Boustany-Kari C. M. (2013). PF-03882845,
a Non-Steroidal Mineralocorticoid Receptor Antagonist, Prevents Renal
Injury with Reduced Risk of Hyperkalemia in an Animal Model of Nephropathy. Front. Pharmacol..

[68] Patel H. K., Bihani T. (2018). Selective Estrogen
Receptor Modulators (SERMs) and
Selective Estrogen Receptor Degraders (SERDs) in Cancer Treatment. Pharmacol. Ther..

