# Supplementary material for: In Silico Decoding of Nonsteroidal Mineralocorticoid Receptor Antagonist Binding: Implications for Drug Design
Source: J Chem Inf Model. 2026 Aug 15;66(17):11140–62. doi: 10.1021/acs.jcim.6c00183 (PMC13580125; doi:10.1021/acs.jcim.6c00183)
Supplement: Supplementary file 1 [file ci6c00183_si_007.pdf]

In Silico decoding of non-steroidal mineralocorticoid receptor antagonist binding: Implications for drug design

Felipe Pérez-Gordillo[a], Laureano E. Carpio[b], Diego Álvarez de la Rosa[c], Mercedes Martín-Martínez[a]*

[a]Instituto de Química Médica (IQM-CSIC), Juan de la Cierva 6, 28006 Madrid, Spain
[b]MolDrug AI Systems SL, Parque Tecnológico de Valencia, 46980 Valencia, Spain
[c]Universidad de La Laguna, 38200 La Laguna, Spain
[*]mmartin@iqm.csic.es

Table of contents

Table S1. 2

Table S2. 2

Figure S1.. 3

Figure S2.. 4

Figure S3.. 5

Figure S4.. 6

Figure S5.. 7

Figure S6.. 8

Figure S7.. 9

Figure S8.. 11

Figure S9.. 11

Figure S10.. 12

Figure S11.. 12

Figure S12.. 13

Figure S13.. 14

Figure S14.. 15

Table S1. PDB codes of the structures that had led to the binding poses discuss in the manuscript.

Compound	Template
**5**	5HCV
**6**	4PF3
**10**	2A3I
**12**	2A3I
**13**	2A3I
**20**	2A3I
**21**	2A3I
**22**	2A3I
**23**	2A3I
**26**	4PF3
**35**	2A3I, 3WFG
**36**	4PF3
**37**	5HCV

Compound	Template
**39**	2A3I, 3WFG
**40**	5HCV
**42**	2A3I
**46**	3WFF
**48**	2A3I
**50**	5L7E
**51**	2A3I
**52**	2A3I
**53**	5HCV
**53**	5HCV
**56**	5HCV
**57**	5HCV

Table S2. Induced-fit docking scores (IFDScore) for the top-ranked pose of the selected compounds bound to AR (1T5Z). Dihydrotestosterone (DHT) was included as reference.

Compound	IFDScore
**DHT**	-585.53
**6**	-587.29
**4R-10**	-584.48
**4R-15**	-583.11

Compound	IFDScore
**23**	-589.16
**26**	-579.93
**36**	-582.95
**49**	-585.51

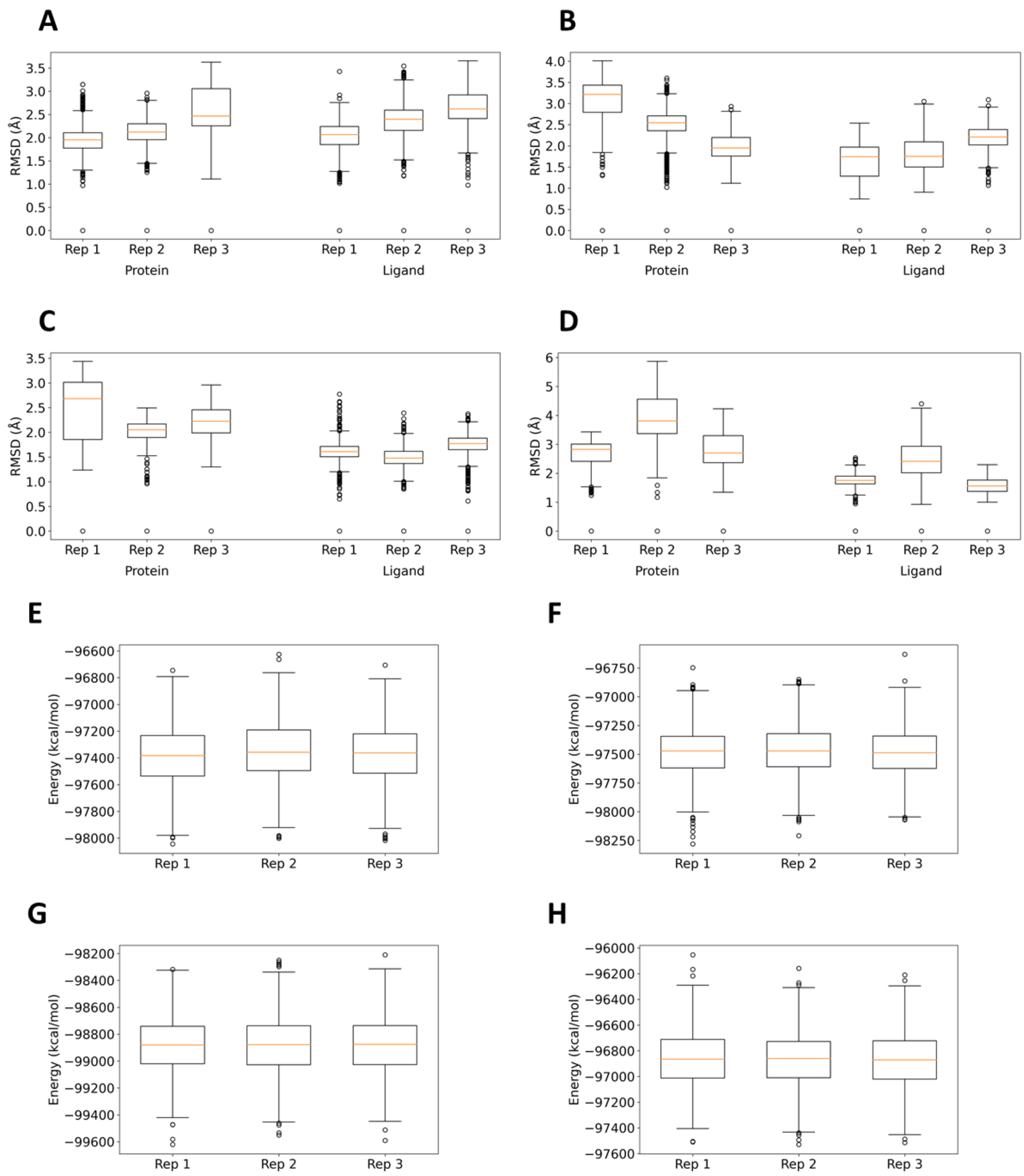

Figure S1. Box-and-whisker plots of protein and ligand RMSD distributions obtained from molecular dynamics replicas: (A) **12**, (B) **20**, (C) Finerenone **23** (D) Finerenone **23** MR without H12 system. Box-and-whisker plots of total system energy distributions obtained from molecular dynamics replicas: (E) **12**, (F) **20**, (G) Finerenone **23** (H) Finerenone **23** MR without H12 system. For each system, three independent replicas are shown for the protein and the ligand. RMSD and Energy values were computed over the simulations. Boxes represent the interquartile range, the central line indicates the median, whiskers extend to 1.5×Interquartile Range, and points denote outliers.

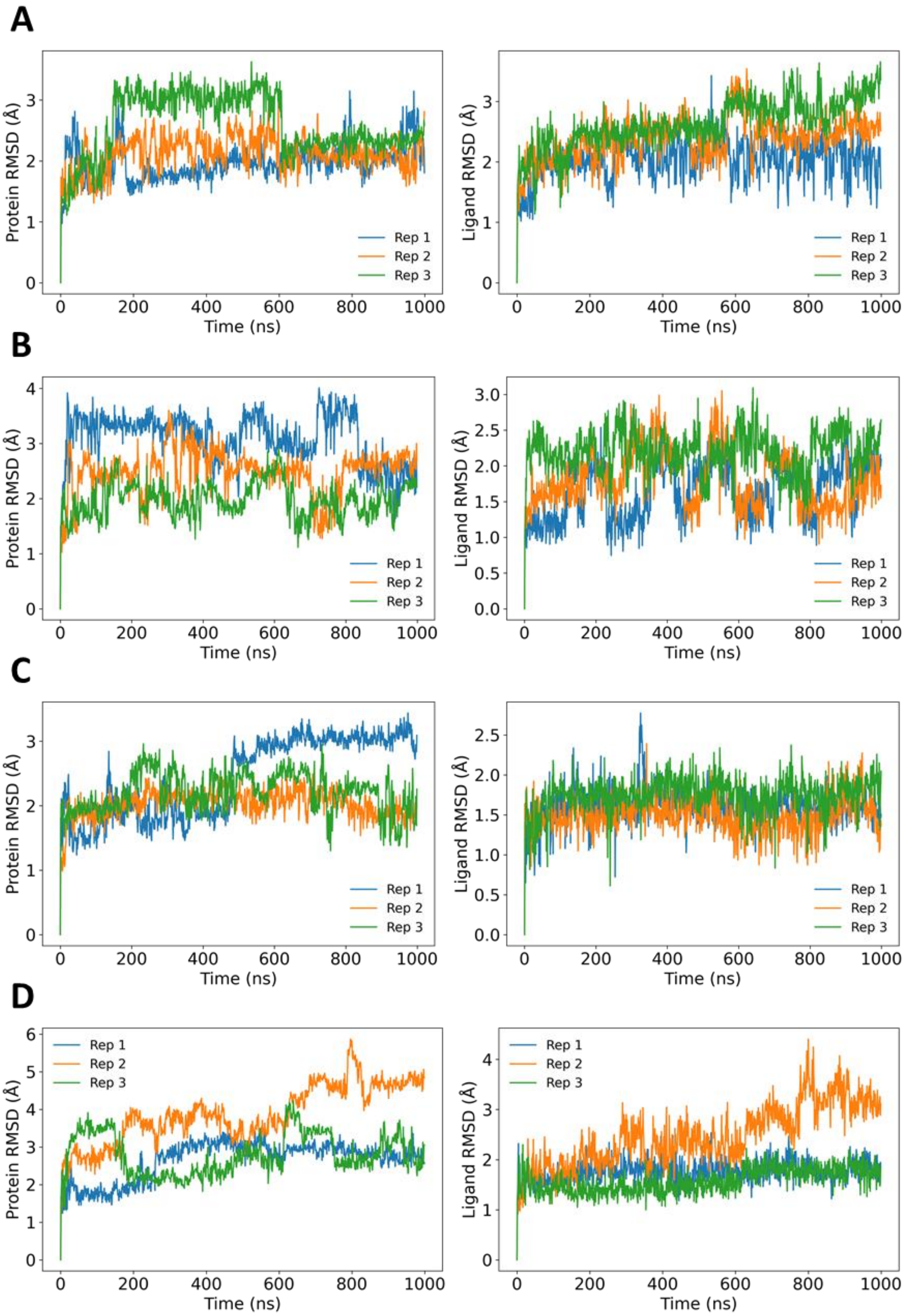

Figure S2. Time evolution of protein and ligand RMSD along independent molecular dynamics replicas. Panels correspond to: (A) **12**, (B) **20**, (C) Finerenone **23** (D) Finerenone **23** MR without H12 system.

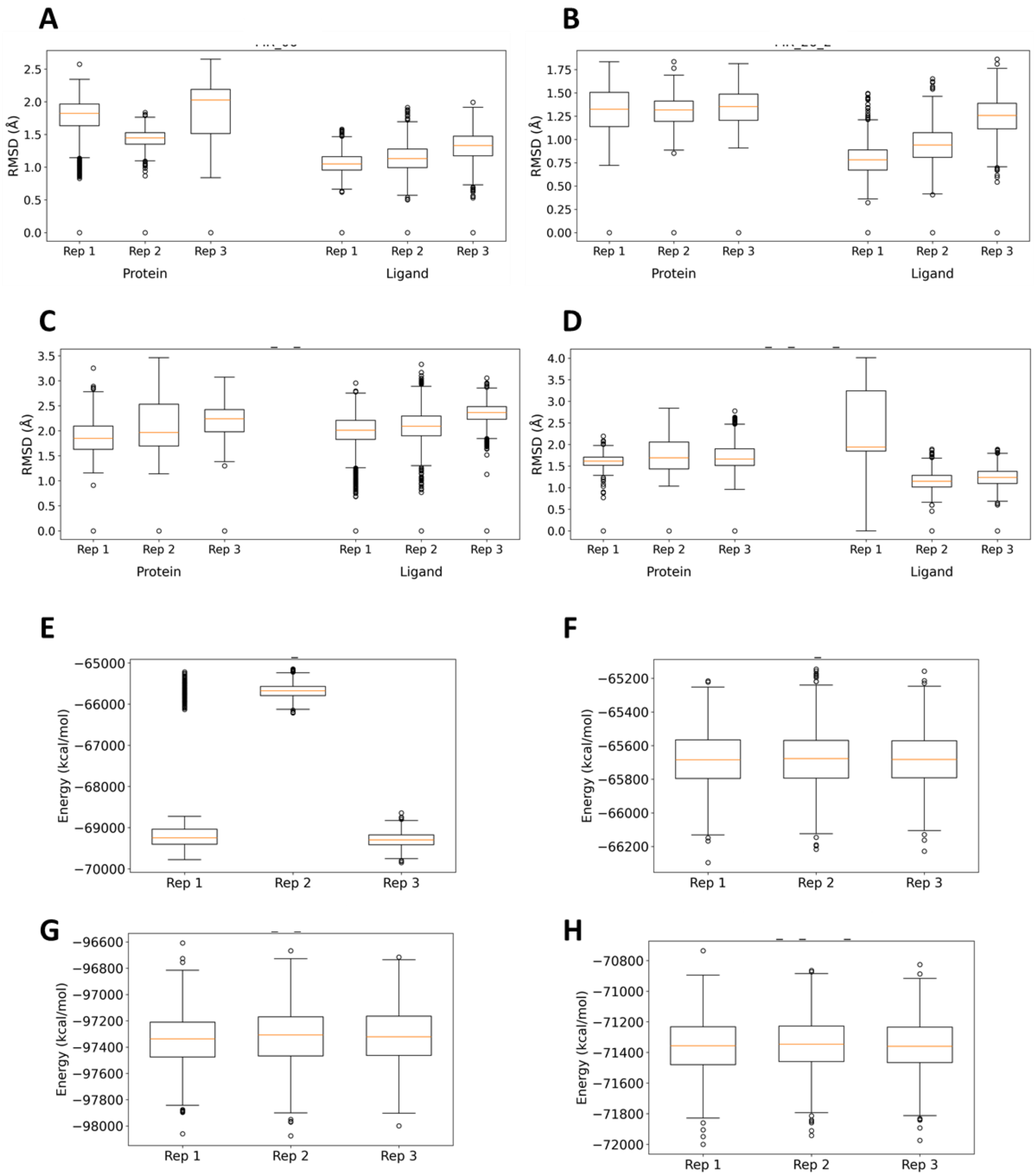

Figure S3. Box-and-whisker plots of protein and ligand RMSD distributions obtained from molecular dynamics replicas: (A) **6**, (B) **26**, (C) **35**, starting from the 2A3I-derived pose, (D) **35**, starting from the 3WFG-derived pose. Box-and-whisker plots of total system energy distributions obtained from molecular dynamics replicas: (E) **6**, (F) **26**, (G) **35**, starting from the 2A3I-derived pose, (H) **35**, starting from the 3WFG-derived pose. For each system, three independent replicas (Rep1–Rep3) are shown. RSMD and Energy values were computed over the simulations. Boxes represent the interquartile range, the central line indicates the median, whiskers extend to 1.5× Interquartile Range, and points denote outliers.

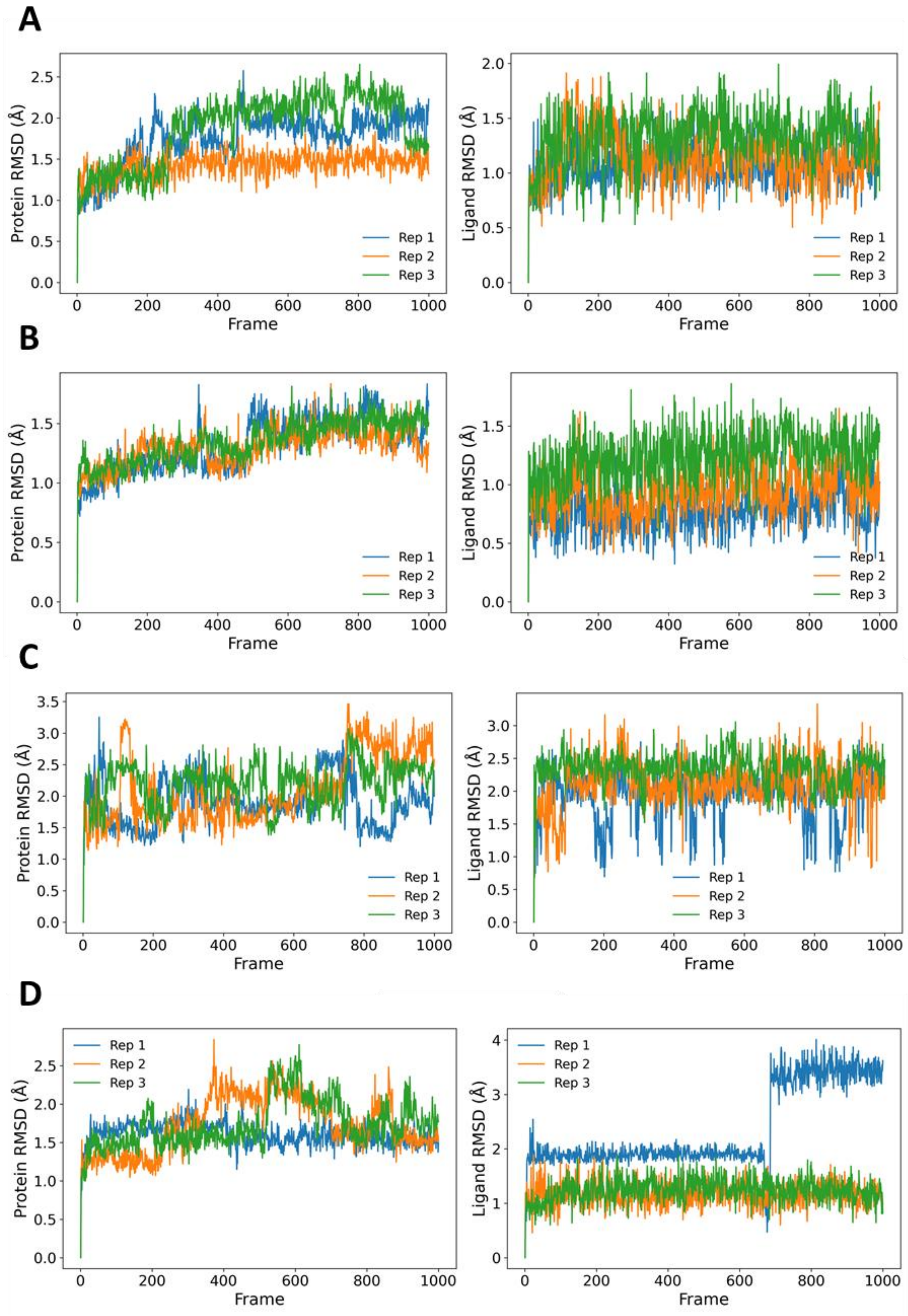

Figure S4. Time evolution of protein and ligand RMSD along independent molecular dynamics replicas. Panels correspond to: (A) **6**, (B) **26**, (C) **35**, starting from the 2A3I-derived pose, (D) **35**, starting from the 3WFG-derived pose.

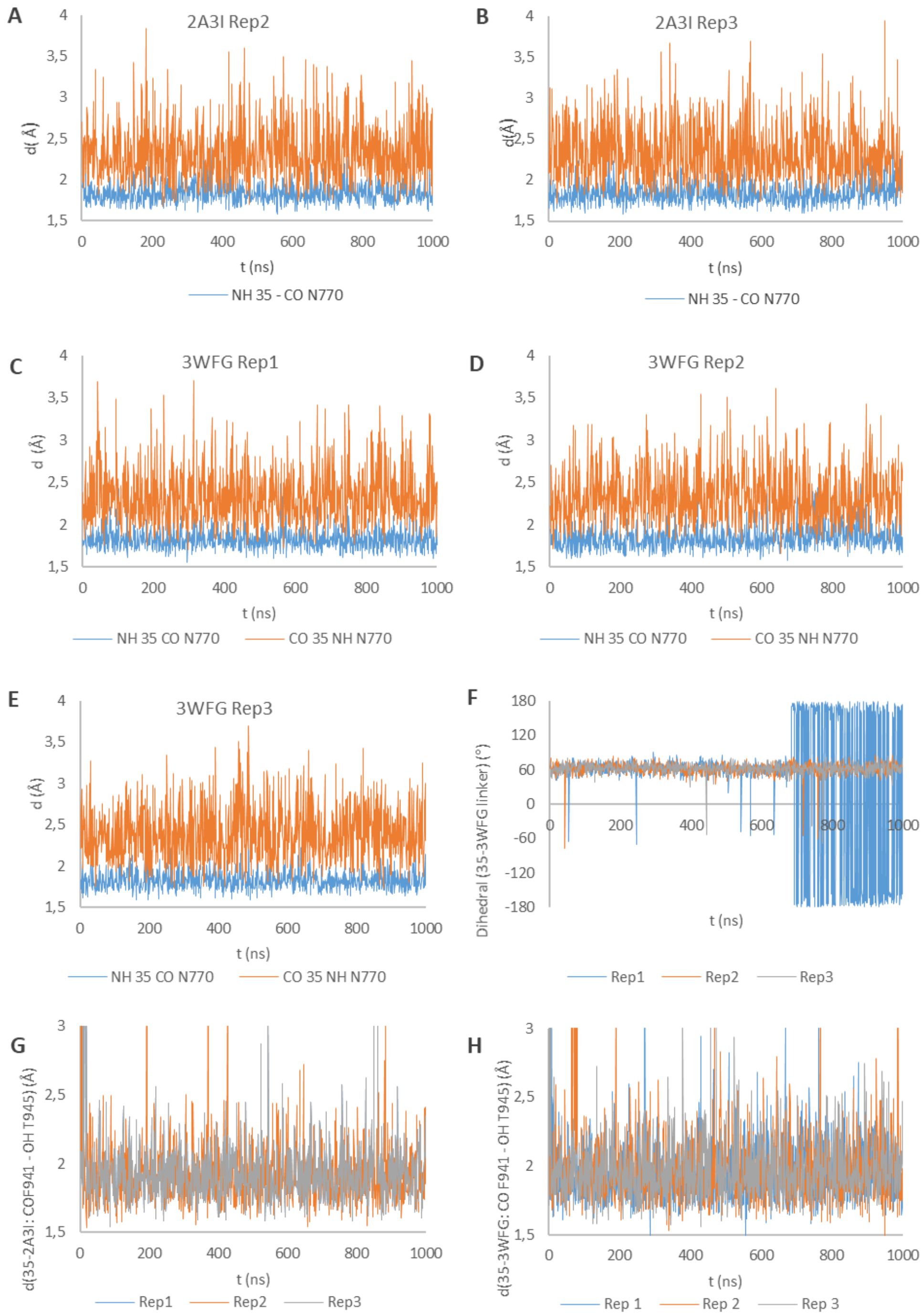

Figure S5. Time evolution of key structural paramenters during the MD simulations of the MR – **35** complex. **(A) (B)** Distances between the cis-amide moiety of compound **35** and Asp770 side chain during the MD initiated from the 2A3I-derived pose (two replicas). **(C-E)** Distances between the cis-amide moiety of compound **35** and Asp770 sidechain during the MD initiated from the 3WFG-derived (three replicas). **(F)** Dihedral angle of **35** linker, starting from the 3WFG-derived pose, along the 1000 ns MD simulation. (**G,H**) Distances between the carbonil oxigen of F941 and the hydroxy hydrogen of Thr945 in the MD studies of the MR – **35** complex initiated from the 2A3I-derived pose (G) or 3WFG-derived pose (H).

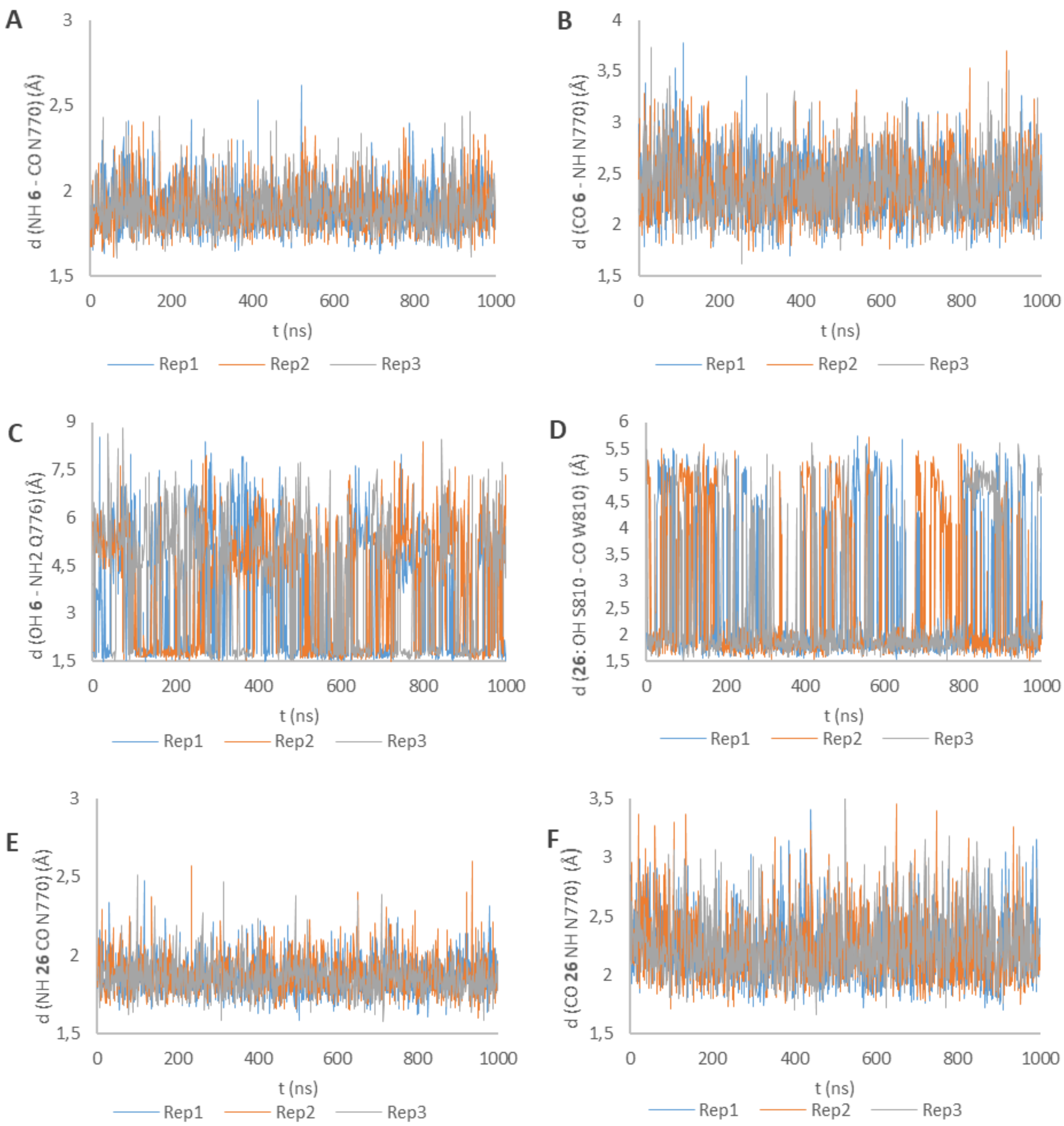

Figure S6. Time evolution of key structural paramenters during the MD simulations of the MR -**6** complex: (**A**) distance between the NH proton of **6** cis-amide moiety and Asn770 side chain CO oxygen (three replicas). (**B**) distance between **6** CO cis-amide oxygen and Asn770 side chain NH_2 proton (three replicas). (**C**) distance between OH hydrogen of **6** cis-amide and Gln776 side chain NH_2 proton (three replicas). Time evolution of key structural paramenters during the MD simulations of the MR -**26** complex: (**D**) distance between OH hydrogen of Ser810 and Trp806 backbone CO oxygen (three replicas). (**E**) distance between the NH proton of 2**6** cis-amide moiety and Asn770 side chain CO oxygen (three replicas). (**F**) distance between 2**6** CO cis-amide oxygen and Asn770 side chain NH_2 proton (three replicas).

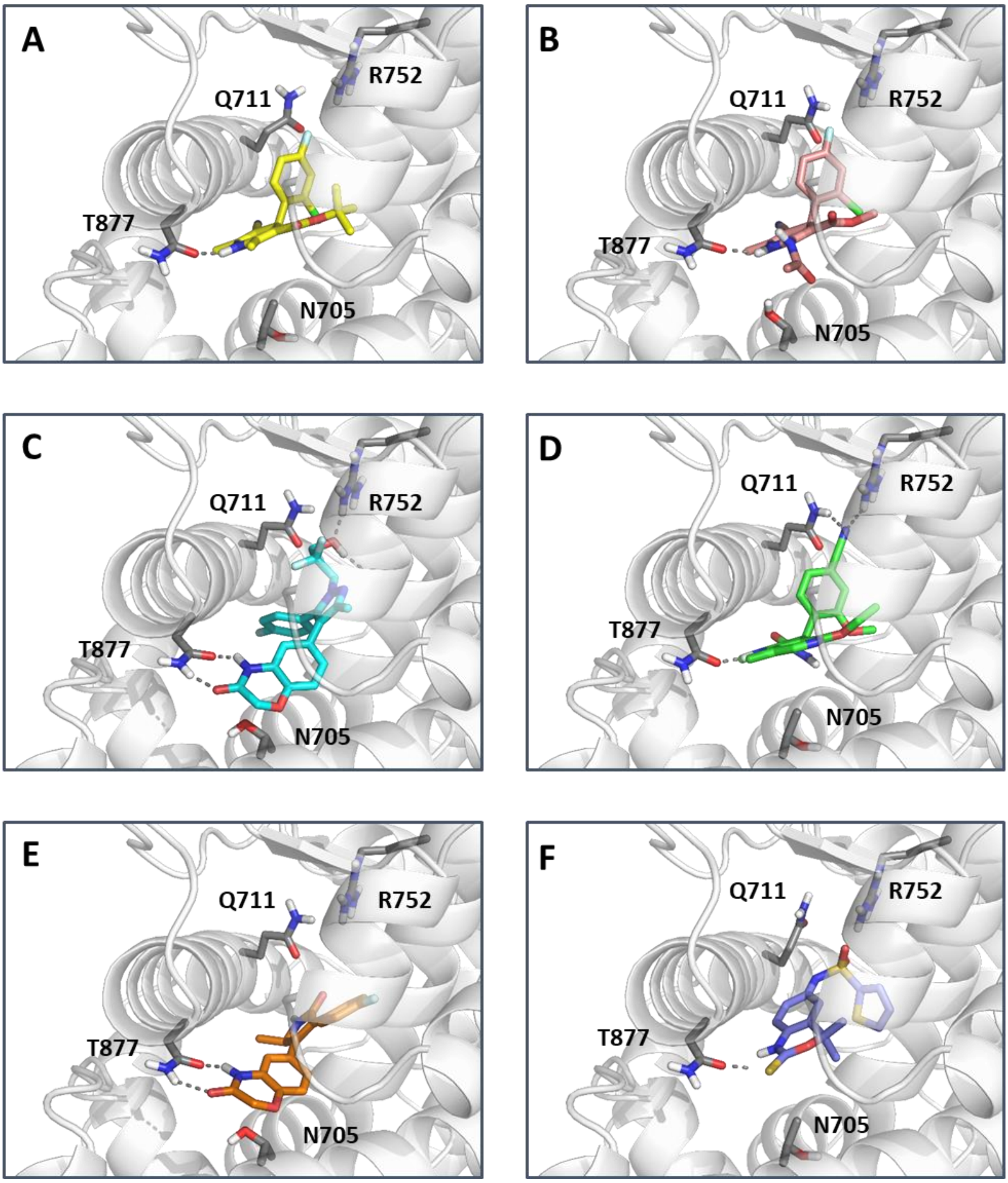

Figure S7. Representative IFD poses of AR (PDB code 1T5Z) in complex with compounds: (**A**) **6**, (**B**) **10**, (**C**) **15,** (**D**) **23**, (**E**) **26** and (**F**) **36**. IFD studies performed using DHPs 4R stereoisomer. Hydrogen bonds are depicted as gray dotted lines. For clarity only polar hydrogens are shown.

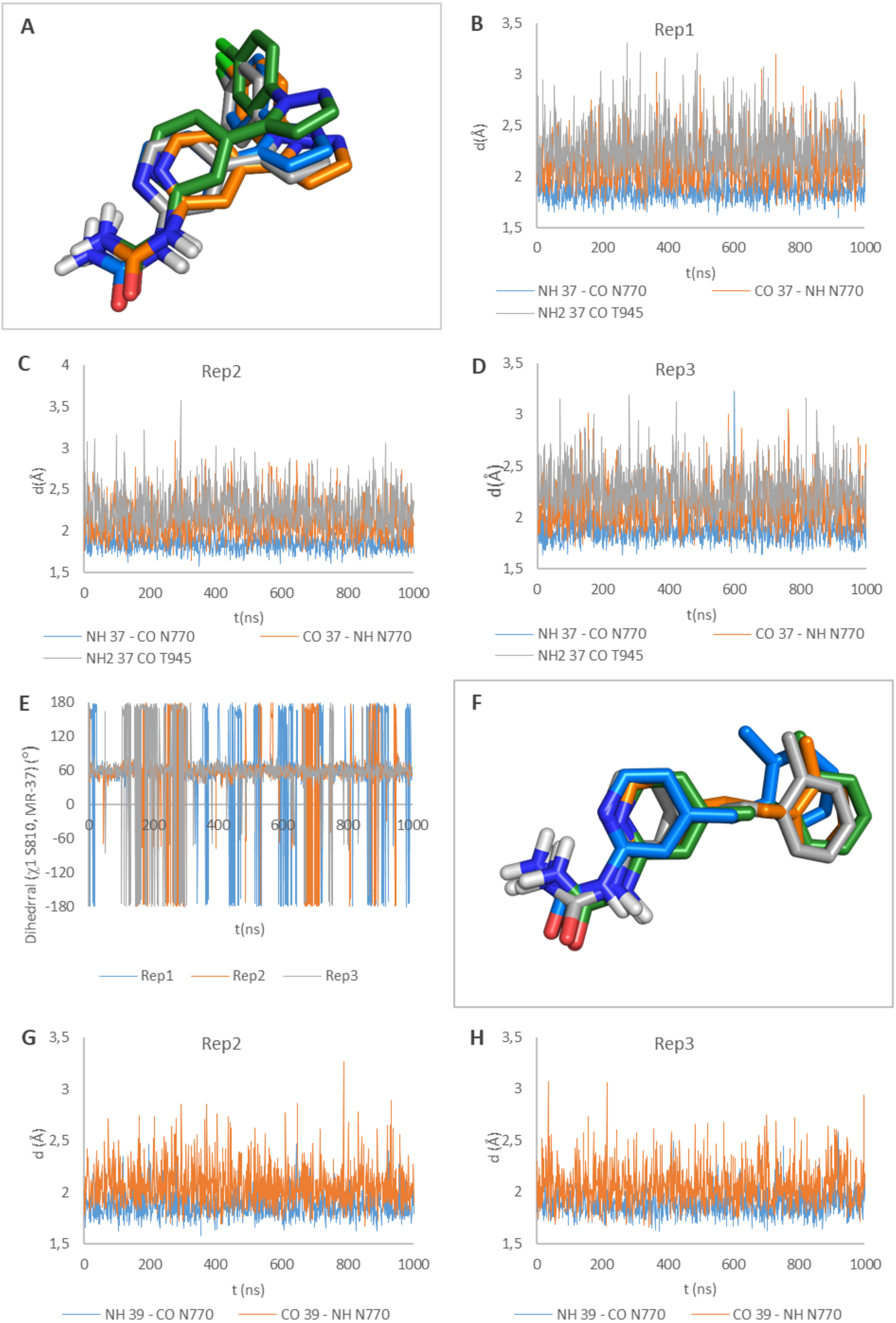
A
B
Rep1
d(Å)
t(ns)
NH 37 - CO N770
CO 37 - NH N770
NH2 37 CO T945
C
Rep2
d(Å)
t(ns)
NH 37 - CO N770
CO 37 - NH N770
NH2 37 CO T945
D
Rep3
d(Å)
t(ns)
NH 37 - CO N770
CO 37 - NH N770
NH2 37 CO T945
E
Dihedrral (χ1 S810, MR-37) (°)
t(ns)
Rep1
Rep2
Rep3
F
G
Rep2
d (Å)
t (ns)
NH 39 - CO N770
CO 39 - NH N770
H
Rep3
d (Å)
t (ns)
NH 39 - CO N770
CO 39 - NH N770

Figure S8. (A) Superimposition of the initial IFD pose (green) and the 1000 ns frames of MR – **37** complexes (three replicas, blue, orange, gray). (**B-D**) Time evolution of key structural paramenters during the MD simulations of the MR – **37** complex: distance between the NH proton of **37** cis-amide moiety and Asn770 side chain CO oxygen, **37** CO cis-amide oxygen and Asn770 side chain NH_2 proton, and **37** NH_2 proton and hydroxy hydrogen of T945. Each plot represents a replica. (**E**) χ1 dihedral angle of Ser810 (**F**) Superimposition of the initial IFD pose (green) and the 1000 ns frames of MR – compound **39** complexes (three replicas, blue, orange, gray). (**G, H**) Time evolution of key distances during the MD simulations of the MR – **39** complex for two replicas: between the NH proton of **39** cis-amide moiety and Asn770 side chain CO oxygen, between **39** CO cis-amide oxygen and Asn770 side chain NH_2 proton.

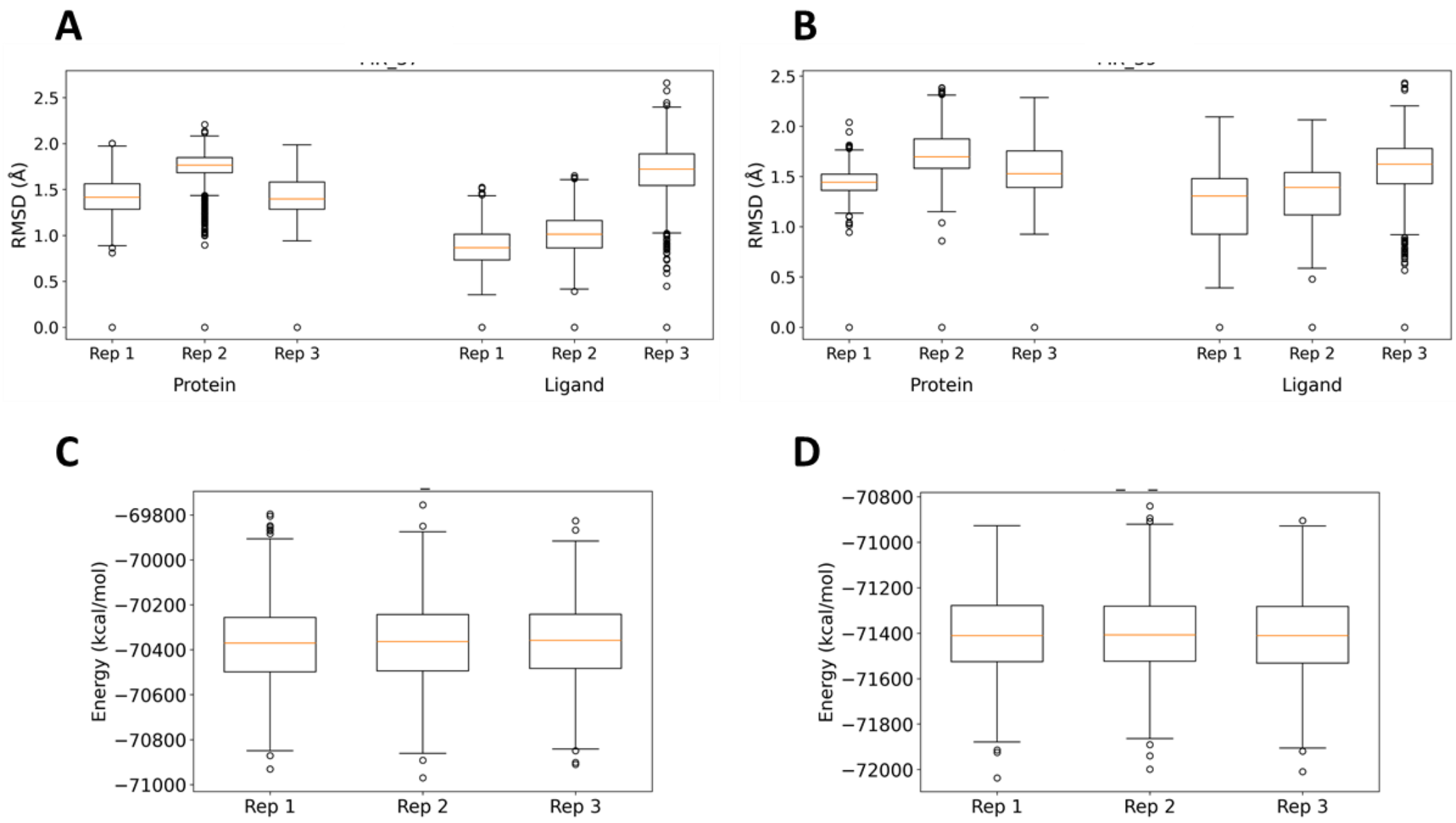

Figure S9. Box-and-whisker plots of protein and ligand RMSD distributions obtained from molecular dynamics replicas: (A) **37**, (B) **39**. Box-and-whisker plots of total system energy distributions obtained from molecular dynamics replicas: (C) **37**, (D) **39**. For each system, three independent replicas are shown for the protein and the ligand. RMSD and Energy values were computed over the simulations. Boxes represent the interquartile range, the central line indicates the median, whiskers extend to 1.5×Interquartile Range, and points denote outliers.

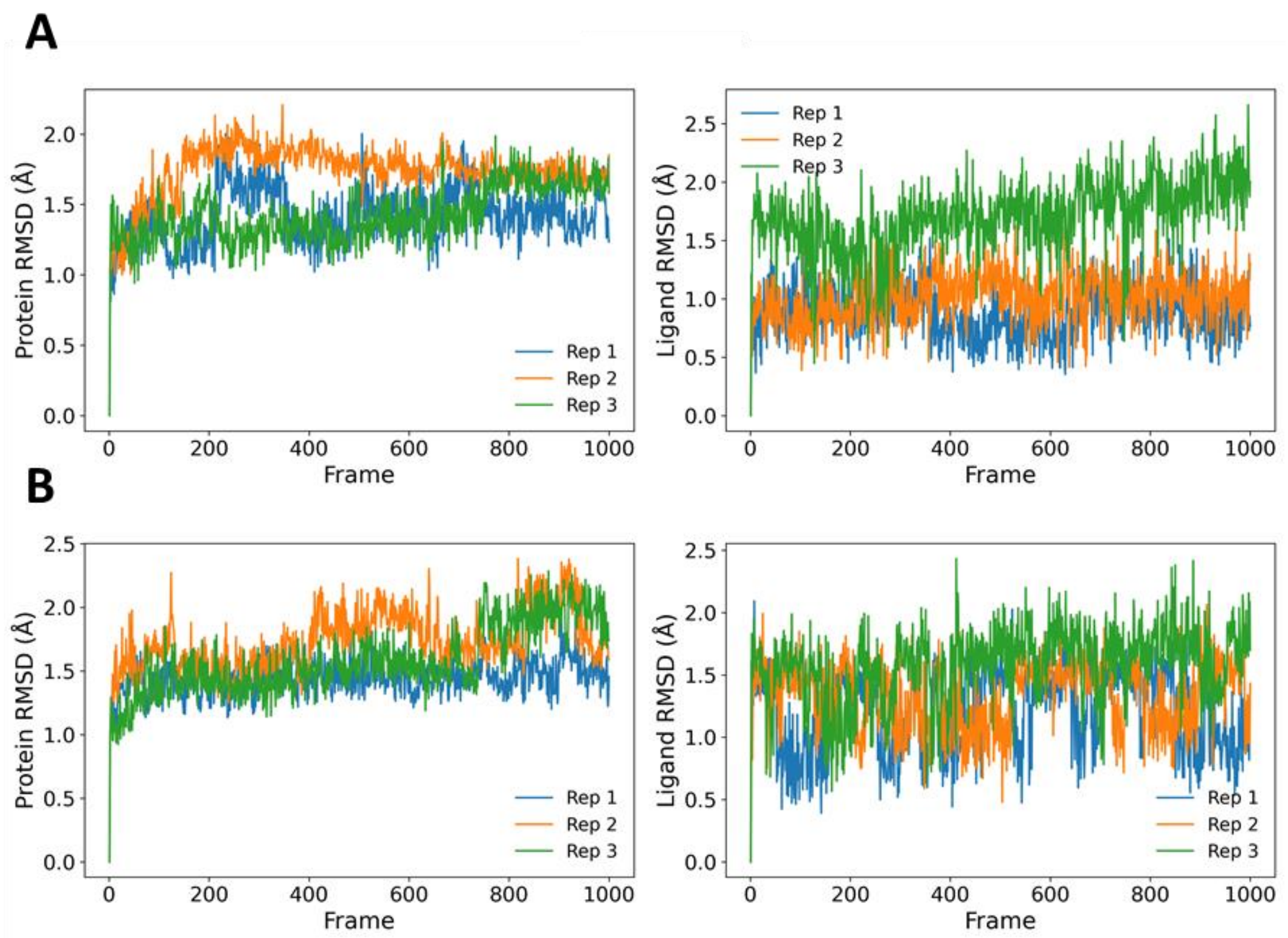

Figure S10. Time evolution of protein and ligand RMSD along independent molecular dynamics replicas. Panels correspond to: (A) **37**, (B) **39**.

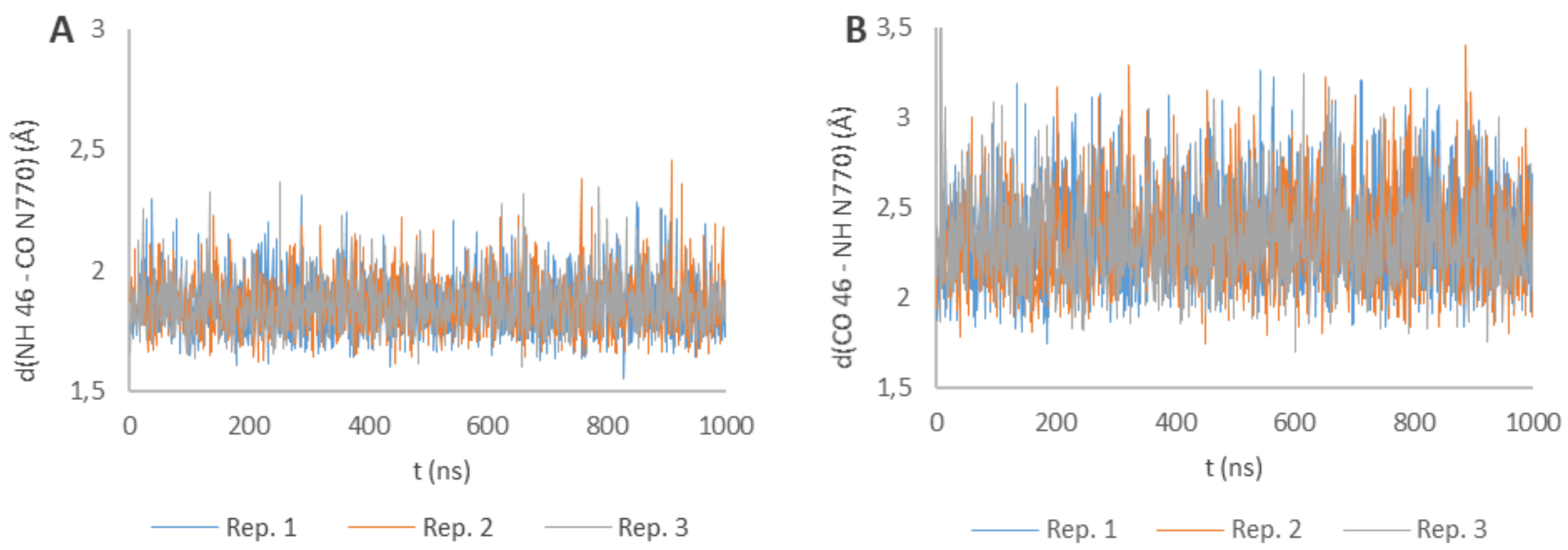

Figure S11. Time evolution of key structural paramenters during the MD simulations of the MR - **46** complex: (**A**) distance between the NH proton of **46** cis-amide moiety and Asn770 side chain CO oxygen (three replicas). (**B**) distance between **46** CO cis-amide oxygen and Asn770 side chain NH_2 proton (three replicas).

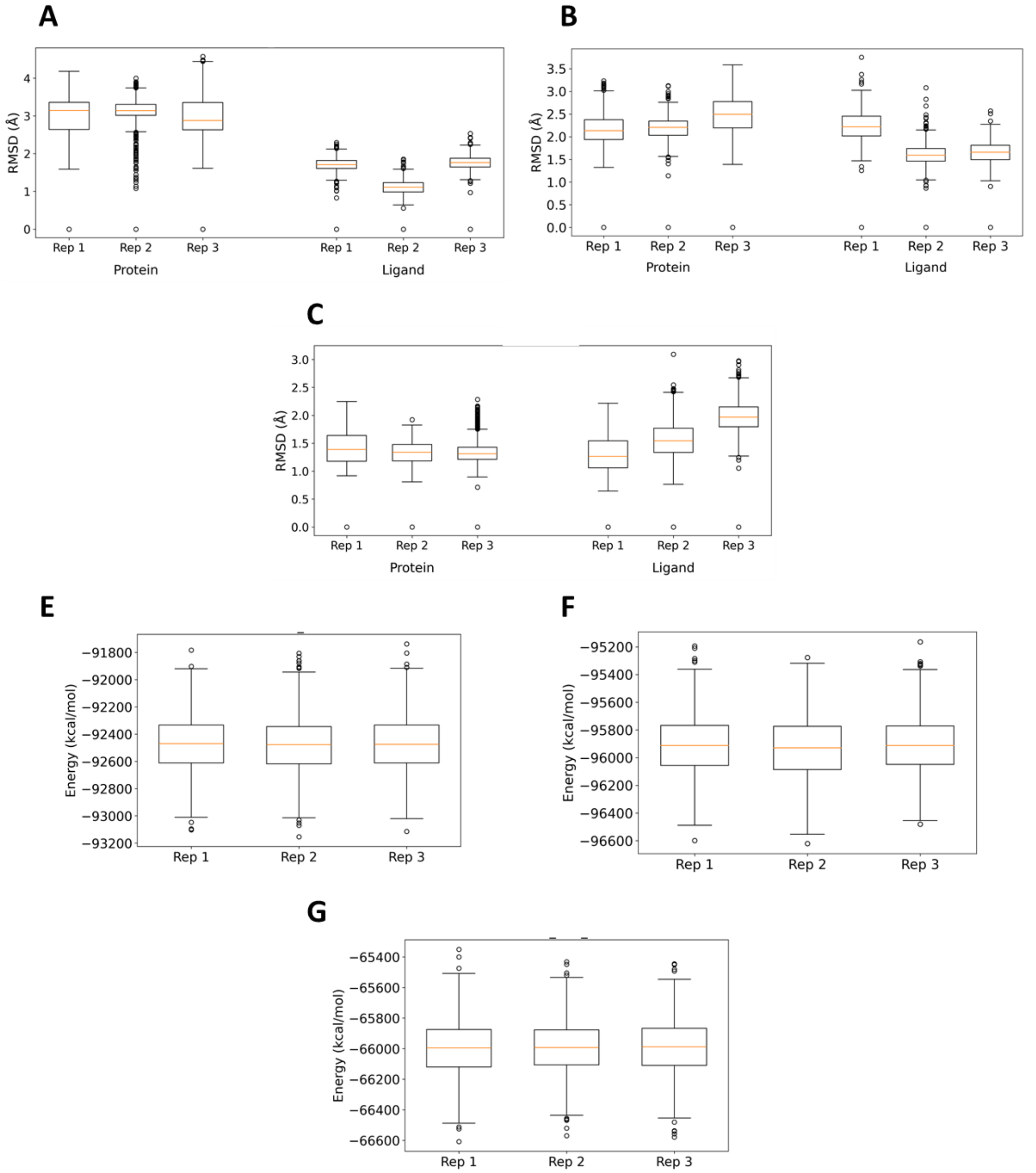

Figure S12. Box-and-whisker plots of protein and ligand RMSD distributions obtained from molecular dynamics replicas: (A) **46**, (B) **48**, (C) **53**. Box-and-whisker plots of total system energy distributions obtained from molecular dynamics replicas: (D) **46**, (E) **48**, (F) **53**. For each system, three independent replicas are shown for the protein and the ligand. RMSD and Energy values were computed over the simulations. Boxes represent the interquartile range, the central line indicates the median, whiskers extend to 1.5×Interquartile Range, and points denote outliers.

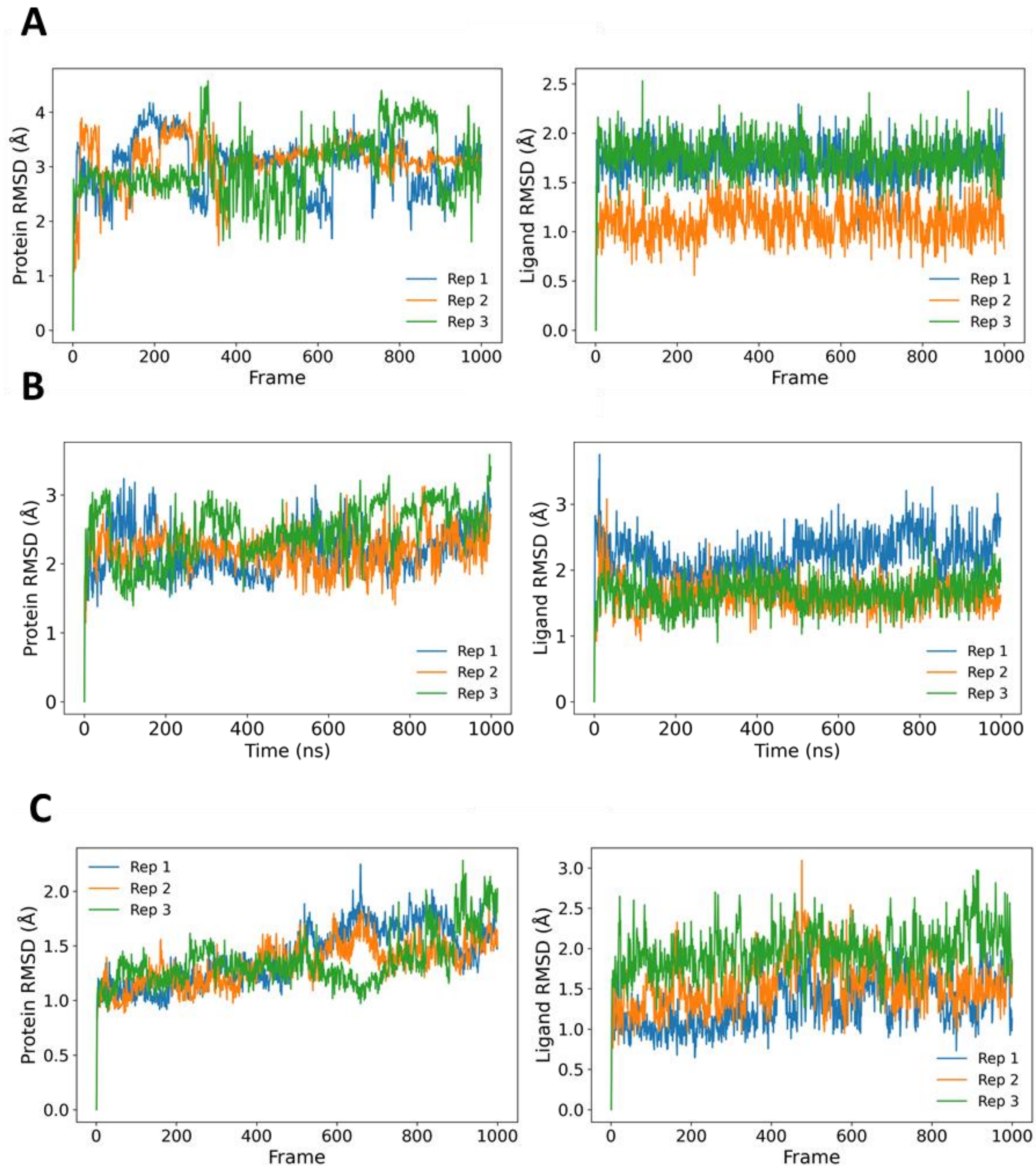

Figure S13. Time evolution of protein and ligand RMSD along independent molecular dynamics replicas. Panels correspond to: (A) **46**, (B) **48**, (C) **53**.

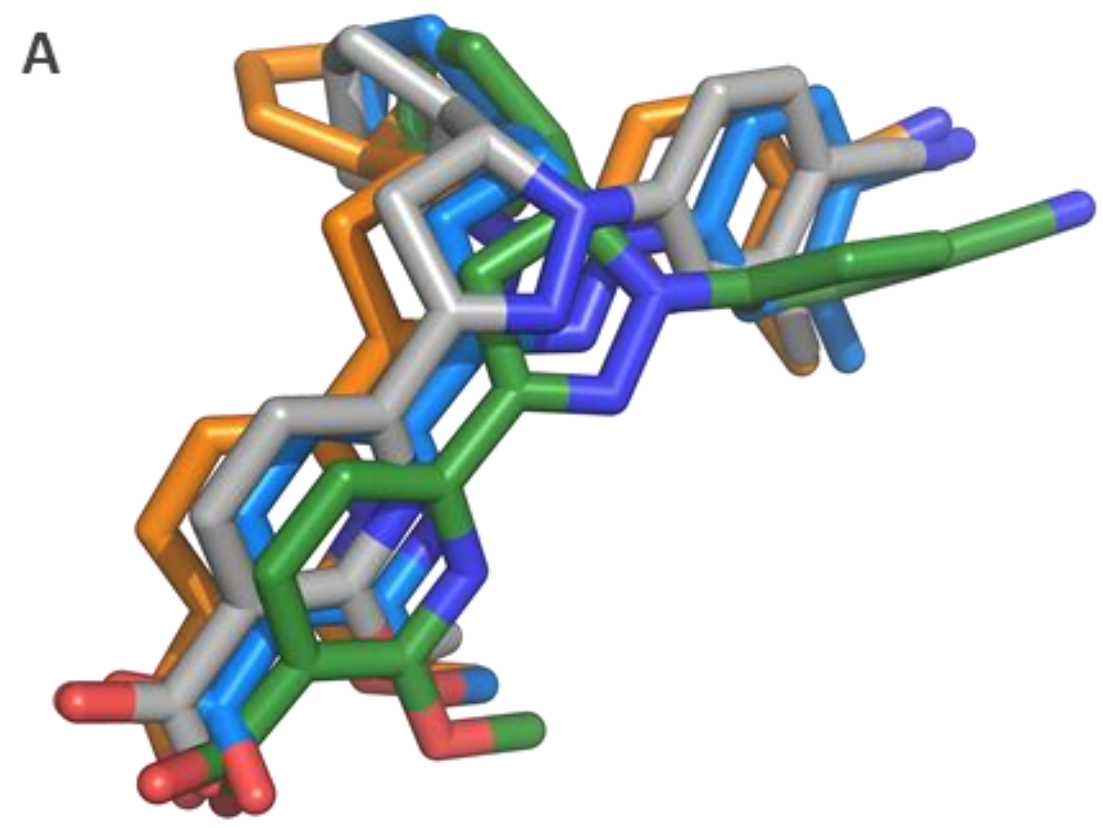

Figure S14. (A) Superimposition of the starting IFD pose (green) and the 1000 ns frames of MR – compound **53** complex (three replicas, blue, orange and gray).
